# Design, Synthesis, Pharmacological Activities, Structure–Activity Relationship, and In Silico Studies of Novel 5-Substituted-2-(morpholinoimino)-thiazolidin-4-ones

**DOI:** 10.1021/acsomega.3c05928

**Published:** 2023-10-04

**Authors:** Yusuf Sıcak, Bedriye Seda Kurşun Aktar, Gizem Tatar Yılmaz, Fatma Aydoğmuş Öztürk, Mehmet Öztürk, Tuğba Taşkın Tok, Emine Elçin Oruç Emre

**Affiliations:** † Department of Medicinal and Aromatic Plants, Köyceğiz Vocational School, Muğla Sıtkı Koçman University, Köyceğiz, Muğla 48800, Turkey; ‡ Department of Hair Care and Beauty Services, Yeşilyurt Vocational School, Malatya Turgut Özal University, Malatya 44210, Turkey; § Department of Biostatistics and Medical Informatics, Faculty of Medicine, Karadeniz Technical University, Trabzon 61080, Turkey; ∥ Department of Chemistry, Faculty of Arts and Sciences, Gaziantep University, Gaziantep 27310, Turkey; ⊥ Department of Chemistry, Faculty of Sciences, Muğla Sıtkı Koçman University, Muğla 48121, Turkey

## Abstract

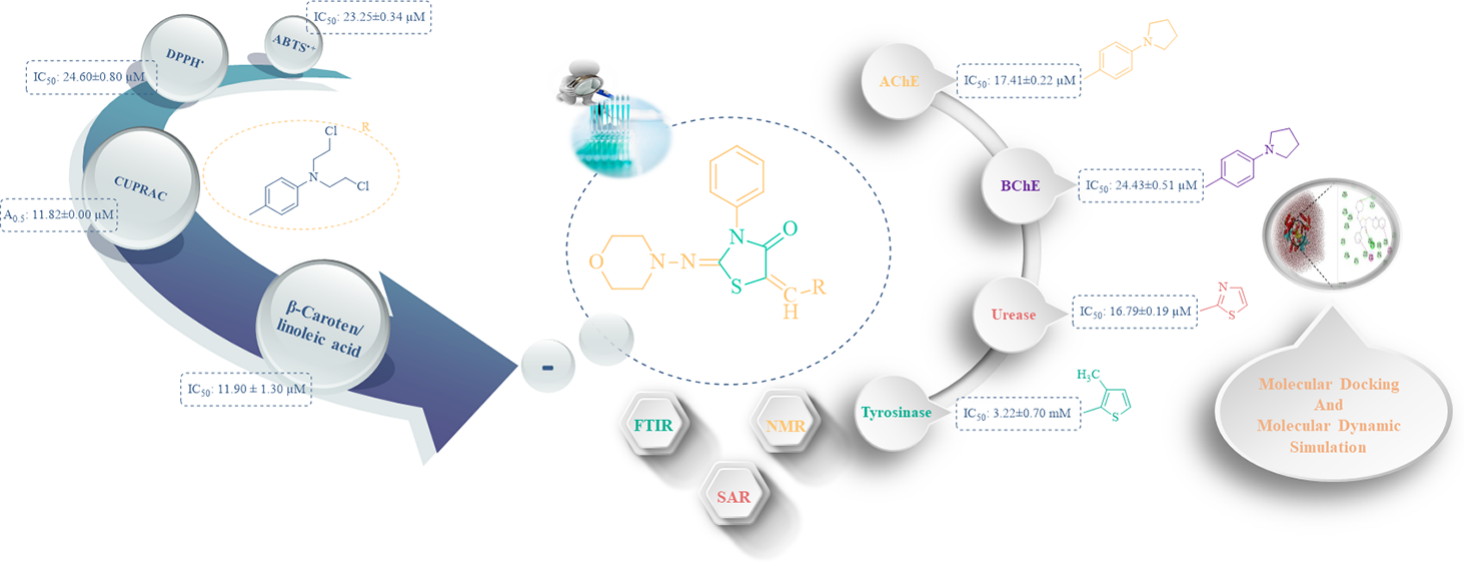

This study is aimed to synthesize morpholine- and thiazolidine-based novel 5-(substituted)benzylidene)-2-(morpholinoimino)-3-phenylthiazolidin-4-ones (**3**–**26**) and characterized by molecular spectroscopy. The synthesized compounds were subjected to antioxidant activity with anticholinesterase, tyrosinase, and urease inhibition activities and evaluated the structure–activity relationship (SAR) of enzyme inhibition activities. Compound **11** was found to be the most active antioxidant. In anticholinesterase inhibition, compound **12** (IC_50_: 17.41 ± 0.22 μM) was the most active against AChE, while compounds **3**–**26** ( except **3**, **8**, and **17**) showed notable activity against BChE. Compounds **17** (IC_50_: 3.22 ± 0.70 mM), **15** (IC_50_: 5.19 ± 0.03 mM), **24** (IC_50_: 7.21 ± 0.27 mM), **23** (IC_50_: 8.05 ± 0.11 mM), **14** (IC_50_: 8.10 ± 0.22 mM), **25** (IC_50_: 8.40 ± 0.64 mM), **26** (IC_50_: 8.76 ± 0.90 mM), and **22** (IC_50_: 9.13 ± 0.55 mM) produced higher tyrosinase inhibition activity. In urease inhibition activity, compounds **20** (IC_50_: 16.79 ± 0.19 μM), **19** (IC_50_: 18.25 ± 0.50 μM), **18** (IC_50_: 20.24 ± 0.77 μM), **26** (IC_50_: 21.51 ± 0.44 μM), **25** (IC_50_: 21.70 ± 0.06 μM), and **24** (IC_50_: 22.49 ± 0.11 μM) demonstrated excellent activities. Besides, the molecular docking study was applied to better understand the inhibitory mechanism between (**1–26**) compounds and enzymes at the molecular level. According to the results of this study, the synthesized compounds exhibited a better binding affinity toward these enzymes compared to the positive control. Further, molecular mechanics Poisson–Boltzmann surface area (MM/PBSA) binding free energy and molecular dynamics (MD) simulation analyses were performed for AChE with compound **26**, which showed high inhibitory activity in silico and in vitro studies. In conclusion, novel morpholine and thiazolidine-based derivative compounds may be pharmacologically effective agents for AChE, BChE, tyrosinase, and urease enzymes.

## 1 Introduction

Reactive oxygen species (ROS) such as superoxide anion (**·**O_2_^–^), hydroxy radical (OH**·**), hydrogen peroxide (H_2_O_2_), nitrogen oxide (NO), peroxynitrite (ONOO^–^), and hypochlorous acid (HOCl) are produced in the living body because of various biological processes.^1−3^ ROS cause serious health problems including cancer, Alzheimer, inflammation, cataracts, atherosclerosis, and reperfusion by attacking several macromolecules (i.e., proteins, enzymes, and DNA).^4^ Antioxidants eliminate free radicals from the body.^5,6^ Although many natural and synthetic antioxidants are used for treatments, there are several reports of side effects.^7,8^

Alzheimer’s disease (AD) starts with a loss of memory for a short time. Subsequently, although it is not fatal, it finally may lead to complete cognitive impairment. However, viral or bacterial infections are neurodegenerative disorders that can lead to the death of the patient.^9^ Alzheimer’s disease is an important public health problem of the age. It is the third biggest cause of death after cardiovascular and cancer diseases in certain countries.^10^ According to Alzheimer’s Disease International, there were more than 50 million patients in 2019 that will be almost three times in 2050.^11^ There is a cholinergic hypothesis for the treatment of AD that supposes the presynaptic reduction of acetylcholine (ACh) due to damage to cholinergic neurons in specific regions of the brain.^12,13^ According to this hypothesis, it increases ACh concentration by inhibition of acetylcholinesterase (AChE), which is responsible for the presynaptic hydrolysis of Ach.^14,15^ Various drugs such as galantamine, donepezil, memantine, Exelon, Razadyne, Aricept, Ebixa, Namzaric, and Cognex are known to heal AD symptoms, although there is no radical solution available.^16^ AChE inhibitors have been reported to cause nausea, gastrointestinal disorders, diarrhea, muscle weakness, and weight loss.^17^

An exposure of the living body to UV radiation can cause skin carcinoma. Normally, the skin responses to the UV radiation by melanogenesis to produce melanin that not only bares UV radiation but also scavenges harmful free radicals produced during the irradiation.^18−20^ Melanin overaccumulation or synthesis can cause hyperpigmentation, lentigine, Parkinson’s neurodegeneration, and malignant melanoma.^21−25^ Although there are many enzymatic reactions in melanin synthesis, tyrosinase and tyrosinase-related proteins (TYRP1 and TYRP2) are the major actors.^26^ Health safety, allergic reactions, low bioavailability, cytotoxicity, stability, and selectivity limit the use of tyrosinase inhibitors.^27−29^ This limitation raises the need to discover new tyrosinase inhibitors.

Urease is another important enzyme that is responsible for adapting the supply and growth of dangerous pathogens by changing the pH of the stomach solution. On the other hand, it plays a role in pharmacological disorders such as urease, urinary and gastrointestinal system infections, stomach ulcers, and duodenal ulcers.^30−32^ Urease (urea amidohydrolase E.C.3.5.1.5.) hydrolyses the urea to ammonia and carbamate.^33,34^ The main function of urease is to provide nitrogen in the form of ammonia, which is necessary for the growth of living organisms.^33,35^ Ureases that produce excessive ammonia can cause hepatic coma, hepatic encephalopathy, urolithiasis, and crusting of the urinary catheter.^35−37^ Since triggering the urease activity of *Helicobacter pylori* in the stomach is effective in the pathogenesis of stomach and peptic ulcers, urease inhibitors have the potential to be used as antiulcer agents.^33,35,38^

According to the pharmacophoric integration approach, two or more pharmacophoric moieties of different bioactive molecules are combined in a single scaffold unit to afford hybrids with improved affinity and efficacy.^23^ Also, this approach can result in compounds with improved selectivity, dual modes of action, and reduced undesired side effects. Herein, we report the design, synthesis, and biological activities of a new series of thiazolidines with different aryl substituents. These compounds were also tested against pharmacologically important targets such as AChE, BChE, tyrosinase, and urease enzymes and free radical scavenging activities. Moreover, the biological activities of the synthesized compounds for target enzymes were evaluated by in silico studies. Thus, this study has contributed to targeted drug discovery and development by providing a better understanding of the inhibition mechanism between these compounds and target enzymes at the molecular level.

## 2 Experimental Section

### Materials and Methods

All chemicals and solvents were analytical grade, purchased from Acros, Alfa Aesar, Sigma-Aldrich, and Merck. Chemical reactions were monitored using thin-layer chromatography (TLC, Merck 60 F_254_). Melting points were determined by SMP20 melting point apparatus and were uncorrected. FTIR spectra were recorded on PerkinElmer Frontier spectrometer by attenuated total reflectance (ATR) apparatus (Waltham, Massachusetts, USA). ^1^H and ^13^C NMR spectra were recorded on Agilent Technologies with 400 and 600 MHz NMR (Agilent, USA). Elemental analyses (CHNS) were performed on a Thermo Scientific Flash 2000 elemental analyzer (Finnigana MAT, USA). Antioxidant and enzyme inhibitory activities were carried out on a 96-well microplate reader, SpectraMax 340PC^384^, Molecular Devices (USA). Spectroscopic data of compounds **1**–**26** are given in the Supporting Information.

### Antioxidant Activities

DMSO solutions of four different concentrations (12.5, 25, 50, and 100 μM) of the synthesized compound **1**–**26** were prepared. DMSO was used as a control, while BHA and α-TOC were used as antioxidant standards. Results are given as 50% inhibition versus concentration (IC_50_) for ABTS^+·^ scavenging activity,^39^ β-carotene-linoleic acid,^40^ and DPPH^·^ assay,^41^ while in the CUPRAC assay,^42^ the results were expressed as 0.500 absorbance vs concentration (A_0.5_).

### Enzyme Inhibition Activities

DMSO solutions of the synthesized compounds (**1–26**) were prepared at four different concentrations, that is, 12.5, 25, 50, and 100 μM for anticholinesterase and urease activity and 12.5, 25, 50, and 100 mM for the tyrosinase inhibitory assay. Anticholinesterase activity of all was performed according to the Ellman’s method using a 96-well microplate reader. Acetylcholinesterase (AChE) from electric eel and butyrylcholinesterase (BChE) from horse serum were used. Acetylthiocholine iodide and butyrylthiocholine chloride were utilized as substrates. DTNB (5,50-dithiobis(2-nitrobenzoic) acid was used as a coloring agent to measure the anticholinesterase activity.^43^ Measurements were obtained in triplicate.

Tyrosinase^44,45^ and urease^46^ inhibitory activities were performed according to the literature using kojic acid with l-mimosine and thiourea were used as standards, respectively. DMSO was used as a control, while results are given as 50% concentration (IC_50_).

### In Silico Studies

#### Structure Preparation of Enzymes and Compounds

Molecular docking requires three-dimensional (3D) structure knowledge of the target enzyme and ligand in order to examine the basic molecular interactions involved in the enzyme-ligand binding mechanism in detail at the molecular level. In this direction, 3D crystal structures of target enzymes of AChE, BChE, tyrosinase, and urease were reached through the protein data bank Web site (http://www.rcsb.org/pdb) (PDB ID: 4EY6, 6QAA, 2Y9X, and 3LA4, respectively). Water and ion molecules were removed from these crystal structures, and appropriate hydrogen atoms were added under physiological pH conditions (pH = 7) using APBS-PDB 2PQR software.^47^ At the same time, the 3D structures of 26 compounds were drawn and geometry and energy were optimized at the DFT/B3LYP/6-31G* level by using Gaussian 09 (G09) software.^48^

#### Molecular Docking Simulations

Following the preparations of enzyme and compound structures were completed, molecular docking was applied to predict the interaction mechanisms of the compounds in the binding site of the target enzymes. Polar hydrogens and Gasteiger atomic charges were assigned to compounds with target enzymes and saved in pdbqt file format. The binding site (active site) of target enzymes was determined according to the location of the binding site of the crystallized ligands with the AGFR1.2 program.^49^ After the required input files were created according to these procedures, molecular docking simulation was performed by AutoDock 4.2^50^ with the Lamanckian genetic algorithm and 100 run steps for each rigid target enzyme and a flexible compound. Within this analysis, the binding free energy (Δ*G*) and inhibition constant (*K*_i_) values between the effector compounds that provide the most appropriate conformational fit to the 3D structure of the target enzymes were estimated.

#### Molecular Dynamic Simulations

Molecular dynamics (MD) simulation was carried out for AChE with compound 26, which was determined as the most effective (best lowest docking score) according to the molecular docking study. For this analysis, topology parameters were prepared with the CGenFF Server^51^ for the ligand and the CHARMM36 all-atom force field^52^ with the TIP3P water model for the protein structure. Then, the system was recognized with a dodecahedron box under a periodic boundary condition, and sodium and chlorine ions were placed in the box to make it electrically neutral.

After the system was prepared, MD simulation was performed in three stages: (i) energy minimization, (ii) equilibration, and (iii) production. In the first stage, a short energy minimization (1000 steps) was applied with the steepest descent method to adjust the steric clashes and inappropriate geometry in the system. Next, the system was equilibration using a 100 ps isochoric–isothermal and isothermal–isobaric (NVT and NPT respectively) ensemble. In the final production phase, 100 ns MD was simulated using a 2 fs time step. The root-mean-square deviation (RMSD), root-mean-square fluctuation (RMSF), and rotation radius (*R*_g_) were examined the complex structure during MD simulations.

#### Molecular Mechanics Poisson–Boltzmann Surface Area (MM-PBSA) Binding Free Energy Calculation Analysis

The binding energies of the protein–ligand complex were estimated using the MM-PBSA method. The molecular mechanical Poisson–Boltzmann surface area (MM-PBSA) approach is a commonly used method to provide efficient and accurate free energy calculations of protein–ligand complexes for drug discovery.^53^ In this study, MM-PBSA analysis was applied with the g mmpbsa program integrated for GROMACS.^54^

The binding free energy (Δ*G*_binding_) of the protein–ligand complex was calculated using the following equations:
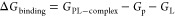

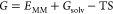

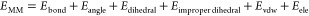

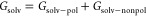
where, Δ*G*_binding_*, G*_PL-complex_, *G*_P_, *G*_L_ represent the binding free energies of ligand, protein–ligand complex, sole protein, and ligand in the solvent, respectively. The free energy (*G*) consists of four terms, which are molecular mechanics (MM) potential energy (*E*_MM_) in vacuum, free energy of solvation (*G*_solv_), and TS (absolute temperature and S is the entropy). *E*_MM_ consists of bond, angle, dihedral and improper dihedral, van der Waals, and electrostatic interaction. *G*_solv_ term has two components: *G*_solv-pol_ and *G*_solv-nonpol_ terms as they represent the polar and nonpolar solute–solvent interactions, respectively.

### Statistical Analysis

All biological activity data were taken in three parallel measurements for four different concentrations of each synthesis sample. The results of the biological activity analyses are presented as IC_50_ values. Data were recorded as mean ± SEM (standard error of the mean) *p* < 0.01.

### Analysis of Physical and Spectroscopic Data of Synthesized Compounds (**1–26**)

#### Synthesis of 1-Morpholino-3-phenylthiyourea (**1**)

4-Aminomorpholine (1 mmol) was dissolved in 5 mL of methanol, and phenylisothiocyanate (1 mmol) was added into this. The reaction mixture was stirred at room temperature for 3 h on a magnetic stirrer. The reaction was quenched with pure water and left for 30 min. Precipitates were filtered and washed with diethyl ether^55^ that produced pure product with no side products. The product was obtained as white amorphous with 80% yield that melted at 220.7–221.0 °C. FTIR νmax (cm^–1^): 3295, 3255 (N–H asymmetric and symmetric stretching band); 3047, 2975 (C–H asymmetric and symmetric stretching band belong to aromatic ring); 2863, 2843 (aliphatic C–H asymmetric and symmetric stretching band belong to aliphatic); 1496 (C=C stretching band); 1396 (C=S stretching band); 1257 (C–O–C stretching band); 1104 (C–N–C stretching band); 1066 (C–N stretching band); 774, 764 (monosubstituted phenyl ring); 479 (N–C–N bending band). ^1^H NMR (400 MHz, DMSO-*d*_6_): δ 2.80 (t, 4H, H_2_), 3.70 (t, 4H, H_1_), 7.15 (t, 1H, H_7_), 7.33 (t, 2H, H_6_), 7.59 (d, *J* = 8 Hz, 2H, H_5_), 9.29 (s, 1H, H_4_), 9.71 (s, 1H, H_3_). ^13^C NMR (100 MHz, DMSO-*d*_6_): δ 54.90 (C_2_), 66.25 (C_1_), 125.21, 125.32, 128.41, 139.45 (Ar-*C’s*), 177.97 (C_3_). Anal. calc. for (C_11_H_15_N_3_OS): C, 55.67; H, 6.37; N, 17.71; S, 13.51; found: C, 56.12; H, 6.35; N, 17.70; S, 13.48.^56^

#### Synthesis of 2-(Morpholinoimino)-3-phenylthiazolidin-4-one (**2**)

After 1 equiv of 1-morpholino-3-phenylthiourea **1** was dissolved with 10 equiv of anhydrous sodium acetate in 7 mL of ethanol under reflux, 2 equiv of ethyl bromoacetate was added to this mixture. After the reaction was completed in 6 h, the solvent was evaporated in vacuum. The white solid obtained extracted by ethyl acetate (15 mL × 3) from distilled water. The organic layer was dried over anhydrous MgSO_4_. Solvent was evaporated in vacuum, and the obtained solid was washed with diethyl ether.^56^ The white amorphs were obtained as pure with 73% yield. mp 228–228.8 °C. FTIR νmax (cm^–1^): 2969, 2953 (C–H asymmetrical and symmetrical stretching band of aromatic ring); 2917, 2892 (aliphatic C–H asymmetrical and symmetrical stretching band); 1720 (C= stretching band); 1604 (C=N stretching band); 1487 (C=C stretching band); 1242 (C–O–C stretching band); 1102 (C–N–C stretching band); 1069 (C–N stretching band). ^1^H NMR (400 MHz, DMSO-*d*_6_): δ 2.59 (t, 4H, H_2_) 3.65 (t, 4H, H_1_), 3.97 (s, 2H, H_8_), 7.31 (d, *J* = 8.0 Hz, 2H, H_5_), 7.49 (t, 1H, H_7_), 7.72 (dd, *J*_*1*_ = 8.0, *J*_*2*_ = 4.0 Hz, 2H, H_6_). ^13^C NMR (100 MHz) (DMSO-*d*_6_/TMS) δ ppm: 32.27 (C_9_), 55.56 (C_2_), 66.05 (C_1_), 128.50, 128.86, 129.45, 135.51 (Ar–C’s), 163.59 (C_3_), 172.81 (C_8_). Anal. calc. for (C_12_H_15_N_3_O_2_S): C, 56.30; H, 5.45; N, 15.15; S: 11.56; found: C, 56.17; H, 5.47; N, 14.98; S, 11.44.

#### Synthesis of 5-(4-Substituted-benzylidene)-2-(morpholinoimino)-3-phenylthiazolidine-4-one (**3–26**)

Twenty millimoles of 2-(morpholinoimino)-3-phenylthiazolidin-4-one (**2**) was dissolved in 5 mL of EtOH having 10 drops of piperidine. Twenty millimoles of the substituted aldehyde was dissolved in 5 mL of EtOH and dropped to the reaction mixture. The reaction was refluxed at 120 °C for 24 h. The reaction was quenched and left for 30 min. The obtained precipitates were washed using ethanol that provided a pure product.^57^ Analytical and spectroscopic data of various derivatives were given in the following lines separately.

#### 5-(Benzylidene)-2-(morpholinoimino)-3-phenylthiazolidine-4-one (**3**)

White solid. Yield: 81%; m.p. 223.7–224.0°C. FTIR νmax (cm^–1^): 2971–2954 (C–H asymmetrical and symmetrical stretching band of aromatic ring); 2918, 2892 (aliphatic C–H asymmetrical and symmetrical stretching band); 1720 (C=O stretching band); 1607 (C=N stretching band); 1498, 1457 (C=C stretching band); 1243 (C–O–C stretching band); 1104 (C–N–C stretching band); 1071 (C–N stretching band). ^1^H NMR (600 MHz, DMSO-*d*_6_) δ ppm: 2.66 (t, 4H, H_2_) 3.69 (t, 4H, H_1_), 7.43–7.47 (m, 4H, H_5_, H_7_, and H_12_), 7.51 (t, 2H, H_6_), 7.56 (t, 2H, H_11_), 7.66 (d, *J* = 7.2 Hz, 2H, H_10_), 7.72 (s, 1H, H_9_). ^13^C NMR (150 MHz, DMSO-*d*_6_) δ ppm: 55.79 (C_2_), 65.91 (C_1_), 121.25, 122.92, 128.56, 129.11, 129.52, 129.77, 130.29, 130.42, 134.15, 135.20 (Ar–C’s, C_9_, and C_10_), 159.08 (C_3_), 166.82 (C_8_). Anal. calc. for (C_21_H_20_N_2_O_2_S): C, 69.20; H, 5.53; N, 7.69; S, 8.80; found: C, 69.16; H, 5.50; N, 7.62; S, 8.78.

#### 5-(4-Bromobenzylidene)-2-(morpholinoimino)-3-phenylthiazolidine-4-one (**4**)

Lemon yellow solid. Yield: 70%; m.p. 240.3–241.1°C. FTIR νmax (cm^–1^): 2970 (C–H asymmetric stretching band of aromatic ring); 2918, 2893 (aliphatic C–H asymmetrical and symmetrical stretching band); 1716 (C=O stretching band); 1605 (C=N stretching band); 1498, 1486, 1456 (C=C stretching band); 1243 (C–O–C stretching band); 1104 (C–N–C stretching band); 1071 (C–N stretching band); 1007 (aromatic C–Br stretching band). ^1^H NMR (400 MHz, DMSO-*d*_6_/TMS): δ ppm: 2.70 (t, 4H, H_2_), 3.72 (t, 4H, H_1_), 7.49–7.46 (m, 3H, H_5_ and H_7_), 7.57–7.53 (m, 2H, H_6_), 7.83 (s, 1H, H_9_), 7.92 (d, *J* = 8.8 Hz, 2H, H_11_), 8.38 (d, *J* = 8.8 Hz, 2H, H_10_). ^13^C NMR (100 MHz, DMSO-*d*_6_/TMS): δ ppm: 55.85 (C_2_), 66.25 (C_1_), 124.81, 127.61, 127.82, 128.52, 129.27, 129.59, 131.34, 135.08, 140.60, 147.51 (Ar–C’s, C_9_, and C_10_), 158.72 (C_3_), 166.51 (C_8_). Anal. calc. for (C_20_H_18_BrN_3_O_2_S): C, 54.06; H, 4.08; N, 9.46; S, 7.22; found: C, 54.00; H, 4.02; N, 9.38; S, 7.16.

#### 5-(4-Hydroxybenzaldehyde)-2-(morpholinoimino)-3 phenylthiazolidine-4-one (**5**)

Lemon yellow solid. Yield: 73%; m.p. 244.6–244.8°C. FTIR νmax (cm^–1^): 3669 (OH stretching band); 2970, 2953 C–H asymmetrical and symmetrical stretching band belong to aromatic ring); 2918, 2864 (aliphatic C–H asymmetrical and symmetrical stretching band); 1720 (C=O stretching band); 1606 (C=N stretching band); 1497, 1456 (C=C stretching band); 1242 (C–O–C stretching band); 1103 (C–N–C stretching band); 1070 (C–N stretching band). ^1^H NMR (400 MHz, DMSO-*d*_6_/TMS): δ ppm: 2.68 (t, 4H, H_2_), 3.72 (t, 4H, H_1_), 6.96 (d, *J* = 8.4 Hz, 2H, H_11_), 7.47–7.42 (m, 3H, H_5_, and H_7_), 7.54–7.51 (m, 4H, H_6_, and H_10_), 7.65 (s, 1H, H_9_), 10.21 (s, 1H, −H_12_). ^13^C NMR (100 MHz) (DMSO-*d*_6_/TMS) δ ppm: 55.82 (C_2_), 65.96 (C_1_), 116.77, 118.47, 125.14, 128.57, 128.98, 129.47, 131.02, 132.71, 135.40, 159.20 (Ar–C, C_9_, and C_10_), 159.84 (C_3_), 167.08 (C_8_). Anal. calc. for (C_20_H_18_N_3_O_3_S): C, 62.97; H, 5.02; N, 11.02; S, 8.41; found: C, 62.91; H, 5.02; N, 11.00; S, 8.36.

#### 5-(4-(Methoxy)benzylidene)-2-(morpholinoimino)-3-phenylthiazolidin-4-one (**6**)

Lemon yellow solid. Yield: 72%; m.p. 245.9–250.1°C. FTIR νmax (cm^–1^): 2957, 2938 (C–H asymmetrical and symmetrical stretching band belong to aromatic ring); 2847, 2827 (aliphatic C–H asymmetrical and symmetrical stretching band); 1711 (C=O stretching band); 1619 (C=N stretching band); 1492 (C=C stretching band); 1246 (C–O–C stretching band); 1107 (C–N–C stretching band); 1072 (C–N stretching band). ^1^H NMR (400 MHz) (DMSO-*d*_6_/TMS) δ ppm: 2.68 (t, 4H, H_2_), 3.72 (t, 4H, H_1_), 3.85 (s, 3H, H_13_), 7.15 (d, *J* = 8.8 Hz, 2H, H_11_), 7.42–7.49 (m, 3H, H_5_, and H_7_), 7.53 (t, *J* = 7.6 Hz, 2H, H_6_), 7.64 (d, *J* = 8.8 Hz, 2H, H_10_), 7.70 (s, 1H, H_9_). ^13^C NMR (100 MHz) (DMSO-*d*_6_/TMS) δ ppm: 55.81 (C_15_), 55.91 (C_2_), 65.98 (C_1_), 115.36, 119.74, 126.68, 128.59, 129.05, 129.51, 130.51, 132.45, 135.32, 160.97 (Ar–C’s, C_9_, and C_10_), 159.20 (C_3_), 167.08 (C_8_). Anal. calc. for (C_21_H_21_N_3_O_3_S): C, 63.78; H, 5.35; N, 10.63; S, 8.11; found: C, 63.69; H, 5.32; N, 10.58; S, 8.06.

#### 5-(3,4-Dimethoxy-benzylidene)-2-(morpholinoimino)-3-phenylthiazolidin-4-one (**7**)

Lemon yellow solid. Yield: 80%; m.p. 241.0–241.9°C. FTIR νmax (cm^–1^): 2970, 2952 (C–H asymmetrical and symmetrical stretching band belong to aromatic ring); 2918, 2892 (aliphatic C–H asymmetrical and symmetrical stretching band); 1719 (C=O stretching band); 1605 (C=N stretching band); 1488, 1456 (C=C stretching band); 1249 (C–O–C stretching band); 1102 (C–N–C stretching band); 1069 (C–N stretching band). ^1^H NMR (400 MHz) (DMSO-*d*_6_/TMS) δ ppm: 2.68 (t, 4H, H_2_), 3.71 (t, 4H, H_1_), 3.84 (s, 3H, H_13_), 3.85 (s, 3H, H_14_), 7.19 (d, *J* = 4.0 Hz 1H, H_11_), 7.26 (dd, *J*_*1,2*_ = 4.0 Hz, 1H, H_12_), 7.29 (d, *J* = 4.0 Hz, 1H, H_10_), 7.43–7.49 (m, 3H, H_5_, and H_7_), 7.53 (t, 2H, H_6_), 7.70 (s, 1H, H_9_). ^13^C NMR (100 MHz) (DMSO-*d*_6_/TMS) δ ppm: 55.80 (C_2_), 56.17 (C_13_), 56.21 (C_14_), 66.00 (C_1_), 112.83, 114.73, 120.18, 123.45, 127.05, 128.53, 129.00, 129.47, 130.90, 135.35, 149.48, 150.91 (Ar–C’s, C_9_, and C_10_), 159.32 (C_3_), 167.03 (C_8_). Anal. calc. for (C_23_H_25_N_3_O_5_S): C, 60.64; H, 5.53; N, 9.22; S, 7.04; found: C, 60.58; H, 5.42; N, 9.17; S, 7.00.

#### 5-(3,4,5-(Trimethoxy)benzylidene)-2-(morpholinoimino)-3-phenylthiazolidin-4-one (**8**)

Lemon yellow solid. Yield: 40%; m.p. 243.1–243.7°C. FTIR νmax (cm^–1^): 2961, 2933 (C–H asymmetrical and symmetrical stretching band belong to aromatic ring); 2879 (aliphatic C–H stretching band); 1709 (C=O stretching band); 1612 (C=N stretching band); 1499, 1454, 1445 (C=C stretching band); 1245 (C–O–C stretching band); 1105 (C–N–C stretching band); 1074 (C–N stretching band). ^1^H NMR (400 MHz) (DMSO-*d*_6_/TMS) δ ppm: 3.39 (t, 4H, H_2_) 3.79 (t, 4H, H_1_), 3.82 (s, 9H, H_11_, and H_12_), 6.84 (s, 2H, H_10_), 7.10–7.13 (m, 1H, H_7_), 7.31 (s, 1H, H_9_), 7.35 (d, *J* = 7.4 Hz, 2H, H_5_), 7.41–7.43 (t, 2H, H_6_). ^13^C NMR (100 MHz) (DMSO-*d*_6_/TMS) δ ppm: 55.87 (C_2_), 56.11 (C_14_), 61.59 (C_15_), 66.18 (C_1_), 111.39, 112.83, 120.24, 121.32, 123.55, 127.05, 128.63, 129.47, 130.96, 135.41, 141.77, 149.56, 151.01 (Ar–C, C_9_, and C_10_), 159.32 (C_3_), 167.03 (C_10_). Anal. calc. for (C_22_H_23_N_3_O_4_S): C, 62.10; H, 5.45; N, 9.88; S, 7.54; found: C, 62.07; H, 5.41; N, 9.77; S, 7.46.

#### 5-(4-Nitrobenzylidene)-2-(morpholinoimino)-3-phenylthiazolidin-4-one (**9**)

Yellow solid. Yield: 51%; m.p. 248.4–249.1°C. FTIR νmax (cm^–1^): 3031, 2966 (C–H asymmetrical and symmetrical stretching band belong to aromatic ring); 2942, 2879 (aliphatic C–H asymmetrical and symmetrical stretching band); 1714 (C=O stretching band); 1621 (C=N stretching band); 1490, 1452 (C=C stretching band); 1341 (NO_2_ symmetrical stretching band); 1243 (C–O–C stretching band); 1104 (C–N–C stretching band); 1066 (C–N stretching band ^1^H NMR (400 MHz) (DMSO-*d*_6_/TMS) δ ppm: 2.70 (t, 4H, H_2_), 3.72 (t, 4H, H_1_), 7.49–7.46 (t, *J* = 6.4 Hz, 3H, H_5_, and H_7_), 7.55 (t, 2H, H_6_), 7.84 (s, 1H, H_9_), 7.92 (d, *J* = 8.8 Hz, 2H, H_10_), 8.39 (d, *J* = 8.8 Hz, 2H, H_11_). ^13^C NMR (100 MHz) (DMSO-*d*_6_/TMS) δ ppm: 55.85 (C_2_), 65.89 (C_1_), 124.80, 127.61, 127.81, 128.51, 129.25, 129.57, 131.32, 135.07, 140.59, 147.52 (Ar–C’s, C_9_, and C_10_), 158.70 (C_3_), 166.49 (C_8_). Anal. calc. for (C_20_H_18_N_4_O_4_S): C, 58.53; H, 4.42; N, 13.65; S, 7.81; found: C, 58.44; H, 4.31; N, 13.52; S, 7.70.

#### 5-(4-Dimethylaminobenzyl)-2-(morpholinoimino)-3-phenylthiazolidine-4-one (**10**)

Yellow solid. Yield: 81%; m.p. 247.5–247.9°C. FTIR νmax (cm^–1^): 2966 (C–H asymmetrical and symmetrical stretching band belong to aromatic ring); 2888, 2840 (aliphatic C–H asymmetrical and symmetrical stretching band); 1703 (C=O stretching band); 1607 (C=N stretching band); 1489, 1459, 1442 (C=C stretching band); 1251 (C–O–C stretching band); 1111 (C–N–C stretching band); 1066 (C–N stretching band). ^1^H NMR (400 MHz) (DMSO-*d*_6_/TMS) δ ppm: 2.68 (t, 4H, H_2_), 3.03 (s, 6H, H_12_), 3.72 (t, 4H, H_1_), 6.86 (d, *J* = 8.8 Hz, 2H, H_11_), 7.40–7.47 (m, 3H, H_5_, and H_7_), 7.50–7.54 (m, 4H, H_6_, and H_10_), 7.62 (s, 1H, H_9_). ^13^C NMR (100 MHz) (DMSO-*d*_6_/TMS) δ ppm: 40.67 (C_15_), 55.81 (C_2_), 66.03 (C_1_), 112.59, 115.48, 121.20, 128.59, 128.87, 129.44, 131.61, 132.43, 135.53, 151.53, (Ar–C, C_9_, and C_10_), 159.40 (C_3_), 167.19 (C_8_). Anal. calc. for (C_22_H_24_N_4_O_2_S): C, 64.68; H, 5.92; N, 13.71; S, 7.85; found: C, 64.53; H, 4.81; N, 13.68; S, 7.76.

#### 5-(4-(Bis(2-chloroethylamino)benzylidene)-2-(morfolinoimino)-3-phenylthiazolidin-4-one (**11**)

Yellow solid. Yield: 73%; m.p. 251.4–251.7°C. FTIR νmax (cm^–1^): 2966, 2911 (C–H asymmetrical and symmetrical stretching band belong to aromatic ring); 2832 (aliphatic C–H asymmetrical and symmetrical stretching band); 1694 (C=O stretching band); 1581 (C=N stretching band); 1489, 1462, 1444 (C=C stretching band); 1252 (C–O–C stretching band); 1104 (C–N–C stretching band); 1074 (C–N stretching band); 719 (C–Cl). ^1^H NMR (400 MHz) (DMSO-*d*_6_/TMS) δ ppm: 2.68 (t, 4H, H_2_) 3.72 (t, 4H, H_1_), 3.79 (t, 4H, H_13_), 3.84 (t, 4H, H_12_), 6.95 (d, *J* = 8.8 Hz, 2H, H_11_), 7.41–7.47 (m, 3H, H_5_, and H_7_), 7.49–7.58 (m, 4H, H_6_, and H_10_), 7.63 (s, 1H, H_9_). ^13^C NMR (100 MHz) (DMSO-*d*_6_/TMS) δ ppm: 41.45 (C_16_), 52.29 (C_15_), 55.82 (C_2_), 66.04 (C_1_), 112.79, 116.65, 122.45, 128.57, 128.91, 129.45, 131.08, 132.69, 135.46, 148.34 (Ar–C’s, C_9_, and C_10_), 159.37 (C_3_), 167.16 (C_8_). Anal. calc. for (C_24_H_25_Cl_2_N_4_O_2_S): C, 57.03; H, 5.18; N, 11.08; S, 6.34; found: C, 57.00; H, 5.13; N, 10.95; S, 6.66.

#### 2-(Morpholinoimino)-3-phenyl-5-(4-(pyrrolidin-1-yl)benzylidene)thiazolidine-4-one (**12**)

Yellow solid. Yield: 74%; m.p. 254.7–255.0°C. FTIR νmax (cm^–1^): 3009, 2918 (C–H asymmetrical and symmetrical stretching band belong to aromatic ring); 2957, 2836 (aliphatic C–H asymmetrical and symmetrical stretching band); 1700 (C=O stretching band); 1607 (C=N stretching band); 1498, 1482, 1452 (C=C stretching band); 1250 (C–O–C stretching band); 1110 (C–N–C stretching band); 1067 (C–N stretching band). ^1^H NMR (400 MHz) (DMSO-*d*_6_/TMS) δ ppm: 1.98 (t, 4H, H_15_), 2.68 (t, 4H, H_2_), 3.34 (t, 4H, H_16_), 3.72 (t, 4H, H_1_), 6.71 (d, *J* = 8.8 Hz, 2H, H_11_), 7.40–7.54 (m, 7H, H_5_, H_6_, H_7_, and H_10_), 7.62 (s, 1H, H_9_). ^13^C NMR (100 MHz) (DMSO-*d*_6_/TMS) δ ppm: 25.43 (C_16_), 47.72 (C_15_), 55.81 (C_2_), 66.04 (C_1_), 112.59, 114.59, 120.63, 128.62, 128.88, 129.46, 131.88, 132.67, 135.53, 148.95 (Ar–C, C_9_, and C_10_), 159.48 (C_3_), 167.24 (C_8_). Anal. calc. for (C_24_H_26_N_4_O_2_S): C, 66.33; H, 6.03; N, 12.89; S, 7.38; found: C, 66.28; H, 6.00; N, 12.75; S, 7.22.

#### 2-(Morpholinoimino)-3-phenyl-5-(4-(piperidin-1-yl)benzylidene)thiazolidine-4-one (**13**)

Yellow solid. Yield: 80%; m.p. 251.6–252.2°C. FTIR νmax (cm^–1^): 2949, 2919 (C–H asymmetrical and symmetrical stretching band belong to aromatic ring); 2893, 2854 (aliphatic C–H asymmetrical and symmetrical stretching band); 1703 (C=O stretching band); 1605 (C=N stretching band); 1512, 1494, 1451 (C=C stretching band); 1243 (C–O–C stretching band); 1109 (C–N–C stretching band); 1067 (C–N stretching band). ^1^H NMR (400 MHz) (DMSO-*d*_6_/TMS) δ ppm: 1.56–1.73 (m, 6H, H_13_, and H_14_), 3.12–3.18 (m, 4H, H_12_), 3.36–3.49 (m, 4H, H_2_), 3.78–3.80 (m, 4H, H_1_), 6.75 (d, *J* = 7.2 Hz, 2H, H_10_), 7.10–7.13 (m, 1H, H_7_), 7.28 (s, 1H, H_9_). 7.10–7.13 (m, 6H, H_5_, H_6_, and H_11_). ^13^C NMR (100 MHz) (DMSO-*d*_6_/TMS) δ ppm: 23.23 (C_17_), 25.43 (C_16_), 47.73 (C_15_), 55.81 (C_2_), 66.04 (C_1_), 112.51, 118.63, 120.57, 125.17, 128.64, 129.48, 131.64, 132.67, 135.24, 150.20 (Ar–C, C_9_, and C_10_), 158.89 (C_3_), 166.59 (C_8_). Anal. calc. for (C_25_H_28_N_4_O_2_S): C, 66.94; H, 6.29; N, 12.49; S, 7.15; found: C, 65.80; H, 6.16; N, 12.41; S, 7.13.

#### 2-(Morfolinoimino)-3-phenyl-5-(4-(morpholin-1-yl)benzylidene)thiazolidine-4-one (**14**)

Orange solid. Yield: 72%; m.p. 251.6–252.2°C. FTIR νmax (cm^–1^): 2968, 2892 (C–H asymmetrical and symmetrical stretching band belong to aromatic ring); 2853, 2833 (aliphatic C–H asymmetrical and symmetrical stretching band); 1705 (C=O stretching band); 1587 (C=N stretching band); 1512, 1493, 1447 (C=C stretching band); 1263 (C–O–C stretching band); 1108 (C–N–C stretching band); 1068 (C–N stretching band). ^1^H NMR (400 MHz) (DMSO-*d*_6_/TMS) δ ppm: 2.68 (t, 4H, H_2_), 3.29 (t, 4H, H_12_), 3.72 (t, 4H, H_1_), 3.76 (t, 4H, H_13_), 7.11 (d, *J* = 8.8 Hz, 2H, H_11_), 7.42–7.47 (m, 3H, H_5_, and H_7_), 7.51–7.56 (m, 4H, H_6_, and H_10_), 7.64 (s, 1H, H_14_). ^13^C NMR (100 MHz) (DMSO-*d*_6_/TMS) δ ppm: 47.41 (C_2_), 55.81 (C_15_), 66.01 (C_1_), 66.34 (C_16_), 114.85, 117.46, 123.88, 128.61, 128.98, 129.49, 131.02, 132.20, 135.41, 152.10 (Ar–C, C_9_, and C_10_), 159.30 (C_3_), 167.13 (C_8_). Anal. calc. for (C_24_H_26_N_4_O_3_S): C, 63.98; H, 5.82; N, 12.44; S, 7.12; found: C, 64.85; H, 5.77; N, 12.39; S, 7.09.

#### 5-(Furan-2-ylmethylene)-2-(morpholinoimino)-3-phenylthiazolidin-4-one (**15**)

Brown solid. Yield: 70%; m.p. 222.8–223.5°C. FTIR νmax (cm^–1^): 2963, 2892 (C–H asymmetrical and symmetrical stretching band belong to aromatic ring); 2850, 2830 (aliphatic C–H asymmetrical and symmetrical stretching band); 1708 (C=O stretching band); 1604 (C=N stretching band); 1490, 1469, 1456 (C=C stretching band); 1250 (C–O–C stretching band); 1108 (C–N–C stretching band); 1075 (C–N stretching band). ^1^H NMR (400 MHz) (DMSO-*d*_6_/TMS) δ ppm: 2.67 (t, 4H, H_2_), 3.71 (t, 4H, H_1_), 6.76 (dd, *J*_*1*_ = 3.2, *J*_*2*_*=*1.6 Hz, 1H, H_11_), 7.06 (d, *J* = 3.2 Hz, 1H, H_10_), 7.42–7.59 (m, 6H, H_5_, H_6_, H_7_, and H_12_), 8.10 (s, 1H, H_9_). ^13^C NMR (100 MHz) (DMSO-*d*_6_/TMS) δ ppm: 55.81 (C_2_), 65.97 (C_1_), 113.78 (C_13_), 117.26 (C_12_), 117.84, (C_11_), 119.90, 128.54, 129.02, 129.46 (Ar–C), 135.27 (C_10_), 147.15 (C_14_), 150.43 (C_11_), 159.66 (C_3_), 166.51 (C_8_). Anal. calc. for (C_18_H_17_N_3_O_3_S): C, 60.83; H, 4.82; N, 11.82; S, 9.02; found: C, 60.80; H, 4.71; N, 11.54; S, 9.00.

#### 2-(Morpholinoimino)-3-phenyl-5-(thiophen-3-ylmethylene) thiazolidine-4-one (**16**)

Beige solid. Yield: 56%; m.p. 221.2–222.0°C. FTIR νmax (cm^–1^): 2972 (C–H stretching band of aromatic ring); 2909 (aliphatic C–H stretching band); 1705 (C=O stretching band); 1618 (C=N stretching band); 1493, 1450 (C= stretching band); 1233 (C–O–C stretching band); 1109 (C–N–C stretching band); 1055 (C–N stretching band). ^1^H NMR (400 MHz) (DMSO-*d*_6_/TMS) δ ppm: 2.39 (s, 3H, H_12_), 2.69 (t, 4H, H_2_), 3.72 (t, 4H, H_1_), 7.16 (d, *J* = 5.0 Hz, 1H, H_10_), 7.44–7.55 (m, 5H, H_6_, H_7_, and H_8_), 7.87 (s, 1H, H_9_), 7.93 (d, *J* = 5.0 Hz, 1H, H_11_). ^13^C NMR (100 MHz) (DMSO-*d*_6_/TMS) δ ppm: 14.52 (C_15_), 55.81 (C_2_), 66.01 (C_1_), 119.67 (C_9_), 121.78, 128.50, 129.48, 131.90 (Ar–C), 129.06 (C_14_), 131.39 (C_13_), 132.15 (C_11_), 135.34 (C_10_), 142.92 (C_12_), 158.93 (C_3_), 166.67 (C_8_). Anal. calc. for (C_18_H_17_N_3_O_2_S_2_): C, 58.20; H, 4.61; N, 11.31; S, 17.26; found: C, 58.11; H, 4.52; N, 11.38; S, 17.15.

#### 5-((3-Methylthiophene-2-yl)methylene)-2-(morpholinoimino)-3-phenylthiazolidin-4-one (**17**)

Light brown solid. Yield: 48%; m.p. 223.5–224.1°C. FTIR νmax (cm^–1^): 3077, 2973 (C–H asymmetrical and symmetrical stretching band belong to aromatic ring); 2890, 2842 (aliphatic C–H asymmetrical and symmetrical stretching band); 1702 (C=O stretching band); 1613 (C=N stretching band); 1498, 1489, 1455 (C=C stretching band); 1224 (C–O–C stretching band); 1106 (C–N–C stretching band); 1075 (C–N stretching band). ^1^H NMR (400 MHz) (DMSO-*d*_6_/TMS) δ ppm: 2.39 (s, 3H, H_12_), 2.69 (t, 4H, H_2_), 3.71 (t, 4H, H_1_), 7.16 (d, *J* = 5.0 Hz, 1H, H_10_), 7.43–7.48 (m, 3H, H_5_, and H_7_), 7.53 (t, 2H, H_6_), 7.87 (s, 1H, H_9_), 7.93 (d, *J* = 5.0 Hz, 1H, H_11_). ^13^C NMR (100 MHz) (DMSO-*d*_6_/TMS) δ ppm: 14.52 (C_15_), 55.81 (C_2_), 66.01 (C_1_), 119.67 (C_9_), 121.78, 128.50, 129.48, 131.90 (Ar–C), 129.06 (C_14_), 131.39 (C_13_), 132.15 (C_11_), 135.34 (C_10_), 142.92 (C_12_), 158.93 (C_3_), 166.67 (C_8_). Anal. calc. for (C_19_H_19_N_3_O_2_S_2_ C, 59.20; H, 4.97; N, 10.90; S, 16.64; found: C, 59.15; H, 4.82; N, 10.88; S, 16.56.

#### 5-((1*H*-Pyrrol-2-yl)methylene)-2-(morpholinoimino)-3-phenylthiazolidin-4-one (**18**)

Pistachio green solid. Yield: 69%; m.p. 229.8–230.4°C. FTIR νmax (cm^–1^): 3314 (N–H stretching band); 2964, 2910 (C–H asymmetrical and symmetrical stretching band belong to aromatic ring); 2859, 2838 (aliphatic C–H asymmetrical and symmetrical stretching band); 1685 (C=O stretching band); 1614 (C=N stretching band); 1486, 1455, 1426 (C=C stretching band); 1241 (C–O–C stretching band); 1109 (C–N–C stretching band); 1040 (C–N stretching band). ^1^H NMR (400 MHz) (DMSO-*d*_6_/TMS) δ ppm: 2.69 (t, 4H, H_2_), 3.73 (t, 4H, H_1_), 6.41 (d, 1H, H_16_), 6.59 (d, *J* = 12.4 Hz, 1H, H_15_), 7.19 (d, *J* = 14.4 Hz, 1H, H_14_), 7.43–7.55 (m, 5H, H_7_, H_8_, and H_9_), 7.67 (d, *J* = 15.4 Hz, 1H, H_12_), 11.63 (d, *J* = 12.0 Hz, 1H, H_17_). ^13^C NMR (100 MHz) (DMSO-*d*_6_/TMS) δ ppm: 55.79 (C_2_), 66.03 (C_1_), 112.17 (C_16_), 113.78 (C_15_), 114.80, (C_11_), 121.04 (C_14_), 124.00, 127.83, 128.60, 128.90 (Ar–C), 129.44 (C_12_), 135.45 (C_13_), 159.42 (C_4_), 167.01 (C_10_). Anal. calc. for (C_18_H_18_N_4_O_2_S): C, 61.00; H, 5.12; N, 15.81; S, 9.05; found: C, 61.00; H, 5.09; N, 15.78; S, 9.01.

#### 5-(1-Methyl-1*H*-pyrrol-2-yl)methylene)-2-(morpholinoimino)-3-phenylthiazolidin-4-one (**19**)

Yellow solid. Yield: 90%; m.p. 232.5–233.9°C. FTIR νmax (cm^–1^): 2976, 2950 (C–H asymmetrical and symmetrical stretching band belong to aromatic ring); 2877, 2862 (aliphatic C–H asymmetrical and symmetrical stretching band); 1702 (C=O stretching band); 1610 (C=N stretching band); 1492, 1481, 1455 (C=C stretching band); 1267 (C–O–C stretching band); 1110 (C–N–C stretching band); 1056 (C–N stretching band). ^1^H NMR (400 MHz) (DMSO-*d*_6_/TMS) δ ppm: 2.67 (t, 4H, H_2_), 3.72 (t, 4H, H_1_), 3.76 (s, 3H, H_13_), 6.34 (brs, 1H, H_11_), 6.58 (d, *J* = 4.0 Hz, 1H, H_10_), 7.16 (brs, 1H, H_12_), 7.41–7.47 (m, 3H, H_5_, and H_7_), 7.52 (t, 2H, H_6_), 7.60 (s, 1H, H_9_). ^13^C NMR (100 MHz) (DMSO-*d*_6_/TMS) δ ppm: 34.26 (C_15_), 55.65 (C_2_), 66.03 (C_1_), 110.35 (C_13_), 115.14 (C_12_), 115.94, (C_9_), 118.32 (C_14_), 128.20, 128.34, 128.57, 129.13 (Ar–C and C_10_), 135.27 (C_11_), 159.43 (C_3_), 166.88 (C_8_). Anal. calc. for (C_19_H_20_N_4_O_2_S): C, 61.94; H, 5.47; N, 15.21; S, 8.70; found: C, 61.85; H, 5.42; N, 15.20; S, 8.66

#### 2-(Morpholinoimino)-3-phenyl-5-(thiazol-2-ylmethyl)thiazolidin-4-one (**20**)

Yellow solid. Yield: 58%; m.p. 252.7–252.9°C. FTIR νmax (cm^–1^): 3066, 3040 (C–H asymmetrical and symmetrical stretching band belong to aromatic ring); 2974, 2948 (aliphatic C–H asymmetrical and symmetrical stretching band); 1714 (C = O stretching band); 1614 (C=N stretching band); 1494, 1475, 1455 (C=C stretching band); 1234 (C–O–C stretching band); 1111 (C–N–C stretching band); 1074 (C–N stretching band). ^1^H NMR (400 MHz) (DMSO-*d*_6_/TMS) δ ppm: 2.68 (t, 4H, H_2_), 3.72 (t, 4H, H_1_), 7.44–7.48 (m, 3H, H_5_ and H_7_), 7.58 (t, 2H, H_6_), 7.95 (s, 1H, H_9_), 8.02 (d, *J* = 3.2 Hz, 1H, H_11_), 8.23 (d, *J* = 3.2 Hz, 1H, H_10_). ^13^C NMR (100 MHz) (DMSO-*d*_6_/TMS) δ ppm: 55.11 (C_2_), 65.94 (C_1_), 119.70 (C_9_), 124.40, 127.36, 128.55, 129.08, 129.47 (Ar–C), 135.11 (C_11_), 145.58 (C_12_), 160.73 (C_3_), 161.99 (C_13_), 166.29 (C_8_). Anal. calc. for (C_17_H_16_N_4_O_2_S_2_): C, 54.82; H, 4.33; N, 15.04; S, 17.22; found: C, 54.70; H, 4.22; N, 15.00; S, 17.16.

#### 2-(Morpholinoimino)-3-phenyl-5-(pyridin-2-ylmethylene)thiazolidin-4-one (**21**)

Light brown solid. Yield: 48%; m.p. 253.6–253.8°C. FTIR νmax (cm^–1^): 2965, 2892 (C–H asymmetrical and symmetrical stretching band belong to aromatic ring); 2849, 2829 (aliphatic C–H asymmetrical and symmetrical stretching band); 1705 (C=O stretching band); 1606 (C=N stretching band); 1491, 1468, 1456 (C=C stretching band); 1245 (C–O–C stretching band); 1107 (C–N–C stretching band); 1074 (C–N stretching band). ^1^H NMR (400 MHz) (DMSO-*d*_6_/TMS) δ ppm: 2.67 (t, 4H, H_2_), 3.72 (t, 4H, H_1_), 7.40–7.49 (m, 4H, H_5_, H_7_, and H_12_), 7.53 (t, 2H, H_6_), 7.77 (s, 1H, H_9_), 7.84 (d, *J* = 7.8 Hz, 1H, H_10_), 7.91–7.95 (m, 1H, H_11_), 8.84 (d, *J* = 4.4 Hz, 1H, H_13_). ^13^C NMR (100 MHz) (DMSO-*d*_6_/TMS) δ ppm: 55.11 (C_2_), 65.94 (C_1_), 123.77 (C_14_), 127.01 (C_12_), 127.37 (C_9_), 129.44, 128.97, 128.62, 127.76 (Ar–C), 135.21 (C_10_), 137.79 (C_13_), 149.88 (C_15_), 152.83 (C_11_), 161.67 (C_3_), 166.76 (C_8_). Anal. calc. for (C_19_H_18_N_4_O_2_S): C, 62.28; H, 4.95; N, 15.29; S, 8.75; found: C, 62.25; H, 4.82; N, 15.18; S, 8.66.

#### 2-(Morpholinoimino)-3-phenyl-5-(pyridin-3-ylmethylene)thiazolidin-4-one (**22**)

Lemon yellow solid. Yield: 47%, m.p. 259.1–260.4°C. FTIR νmax (cm^–1^): 2975, 2892 (C–H asymmetrical and symmetrical stretching band belong to aromatic ring); 2859, 2836 (aliphatic C–H asymmetrical and symmetrical stretching band); 1708 (C=O stretching band); 1594 (C=N stretching band); 1491, 1456, 1411 (C=C stretching band); 1242 (C–O–C stretching band); 1101 (C–N–C stretching band); 1067 (C–N stretching band). ^1^H NMR (400 MHz) (DMSO-*d*_6_/TMS) δ ppm: 2.69 (t, 4H, H_2_), 3.72 (t, 4H, H_1_), 7.45–7.49 (m, 3H, H_5_, and H_7_), 7.54 (t, 2H, H_6_), 7.60 (s, 1H, H_9_), 7.77 (s, 1H, H_9_), 8.05 (d, *J* = 8.0 Hz, 1H, H_11_), 8.63 (dd, *J*_1_ = 4.8, *J*_2_ = 1.4 Hz, 1H, H_13_), 8.89 (d, *J* = 2.0 Hz, 1H, H_10_). ^13^C NMR (100 MHz) (DMSO-*d*_6_/TMS) δ ppm: 55.83 (C_2_), 65.92 (C_1_), 124.68 (C_9_), 125.41 (C_14_), 127.04, 128.51, 129.19, 129.55 (Ar–C), 130.36 (C_13_), 135.16 (C_10_), 136.55 (C_11_), 150.45 (C_15_), 151.59 (C_11_), 158.80 (C_3_), 166.52 (C_8_). Anal. calc. for (C_19_H_18_N_4_O_2_S): C, 62.28; H, 4.95; N, 15.29; S, 8.75; found: C, 62.27; H, 4.88; N, 15.28; S, 8.76.

#### 2-(Morpholinoimino)-3-phenyl-5-(pyridin-4-ylmethylene)thiazolidin-4-one (**23**)

Yellow solid. Yield: 52%; m.p. 264.7–264.9°C. FTIR νmax (cm^–1^): 3035, 2973 (C–H asymmetrical and symmetrical stretching band belong to aromatic ring); 2882, 2858 (aliphatic C–H asymmetrical and symmetrical stretching band); 1710 (C=O stretching band); 1625 (C=N stretching band); 1488, 1453 (C=C stretching band); 1234 (C–O–C stretching band); 1108 (C–N–C stretching band); 1069 (C–N stretching band). ^1^H NMR (400 MHz) (DMSO- *d*_6_/TMS) δ ppm: 2.70 (t, 4H, H_2_), 3.72 (t, 4H, H_1_), 7.46–7.49 (m, 2H, H_5_, and H_7_), 7.54 (t, 2H, H_6_), 7.60 (d, *J* = 6.0 Hz, 2H, H_10_), 7.70 (s, 1H, H_9_), 8.76 (d, *J* = 6.0 Hz, 2H, H_11_). ^13^C NMR (150 MHz) (DMSO-*d*_6_/TMS) δ ppm: 55.85 (C_2_), 65.89 (C_1_), 123.88 (C_9_), 127.38 (C_12_), 128.24, 128.49, 129.24, 129.56 (Ar–C), 135.09 (C_10_), 141.22 (C_11_), 151.07 (C_13_), 158.66 (C_3_), 166.42 (C_8_). Anal. calc. for (C_19_H_18_N_4_O_2_S): C, 62.28; H, 4.95; N, 15.29; S, 8.75; found: C, 62.24; H, 4.83; N, 15.21; S, 8.70.

### 5-((1*H*-Indol-3-yl)methylene)-2-(morpholinoimino)-3-phenylthiazolidin-4-one (**24**)

Yellow matter solid. Yield: 91%; m.p. 260.3–260.9°C. FTIR νmax (cm^–1^): 3269 (N–H stretching band); 2974 (C–H stretching band belong to aromatic ring); 2918, 2857 (aliphatic C–H asymmetrical and symmetrical stretching band); 1677 (C=O stretching band); 1616 (C=N stretching band); 1515, 1494, 1458 (C=C stretching band); 1232 (C–O–C stretching band); 1112 (C–N–C stretching band); 1078 (C–N stretching band). ^1^H NMR (400 MHz) (DMSO-*d*_6_/TMS) δ ppm: 2.70 (t, 4H, H_2_), 3.74 (t, 4H, H_1_), 7.19 (t, 1H, H_13_), 7.27 (t, 1H, H_14_), 7.43–7.49 (m, 3H, H_5_, and H_7_), 7.51–7.56 (m, 3H, H_6_, and H_12_), 7.81 (s, 1H, H_9_), 7.87 (d, *J* = 7.8 Hz, 1H, H_15_), 8.02 (s, 1H, H_10_), 12.00 (s, 1H, H_11_). ^13^C NMR (150 MHz) (DMSO-*d*_6_/TMS) δ ppm: 55.82 (C_2_), 66.03 (C_1_), 111.22, 112.83, 115.75, 118.79, 121.29, 122.86, 123.41, 127.28, 128.64, 128.77, 128.89, 129.46 (Ar–C, indole-C, and C_9_), 135.56 (C_10_), 136.59 (C_13_), 159.17 (C_3_), 166.89 (C_8_). Anal. calc. for (C_22_H_20_N_4_O_2_S): C, 65.33; H, 4.98; N, 13.85; S, 7.93; found: C, 65.28; H, 4.82; N, 13.78; S, 7.90.

### 5-((1*H*-Indole-5-yl)methylene)-2-(morpholinoimino)-3-phenylthiazolidin-4-one (**25**)

Yellow solid. Yield: 91%; m.p: 262.5–263.4°C. FTIR νmax (cm^–1^): 3397 (N–H stretching band); 2976, 2914 (C–H asymmetrical and symmetrical stretching band belong to aromatic ring); 2856, 2841 (aliphatic C–H asymmetrical and symmetrical stretching band); 1691 (C=O stretching band); 1615 (C=N stretching band); 1497, 1490, 1465 (C=C stretching band); 1241 (C–O–C stretching band); 1107 (C–N–C stretching band); 1071 (C–N stretching band). ^1^H NMR (400 MHz) (DMSO-*d*_6_/TMS) δ ppm: 2.69 (t, 4H, H_2_), 3.73 (t, 4H, H_1_), 6.61 (brs, 1H, H_14_), 7.42–7.60 (m, 8H, H_5_, H_6_, H_7_, H_10_, H_11_, and H_13_), 7.85 (s, 1H, H_9_), 7.91 (s, 1H, H_15_), 11.45 (s, 1H, H_12_). ^13^C NMR (150 MHz) (DMSO-*d*_6_/TMS) δ ppm: 55.84 (C_2_), 65.98 (C_1_), 102.72, 112.89, 118.16, 123.70, 123.95, 125.20, 127.73, 128.63, 128.98, 129.49, 133.08, 137.07 (Ar–C, indole-C, and C_9_), 135.44 (C_10_), 159.37 (C_3_), 167.15 (C_8_). Anal. calc. for (C_22_H_20_N_4_O_2_S): C, 65.33; H, 4.98; N, 13.85; S, 7.93; found: C, 65.31; H, 4.94; N, 13.81; S, 7.90.

### 2-(Morpholinoimino)-3-phenyl-5-(quinolin-2-ylmethylene)thiazolidin-4-one (**26**)

Mustard solid. Yield: 66%; m.p: 270.1–271.2°C. FTIR νmax (cm^–1^): 2972, 2907 (C–H asymmetrical and symmetrical stretching band belong to aromatic ring); 2883, 2840 (aliphatic C–H asymmetrical and symmetrical stretching band); 1707 (C=O stretching band); 1611 (C=N stretching band); 1498, 1490, 1451 (C=C stretching band); 1238 (C–O–C stretching band); 1103 (C–N–C stretching band); 1067 (C–N stretching band). ^1^H NMR (400 MHz) (DMSO-*d*_6_/TMS) δ ppm: 2.71 (t, 4H, H_2_), 3.78 (t, 4H, H_1_), 7.46–7.49 (m, 3H, H_5_, and H_7_), 7.55 (t, 2H, H_6_), 7.68 (t, 1H, H_13_), 7.87–7.95 (m, 3H, H_9_, H_10_, and H_14_), 8.03 (d, *J* = 8.0 Hz, 1H, H_12_), 8.15 (d, *J* = 8.4 Hz, 1H, H_15_), 8.47 (d, *J* = 8.4 Hz, 1H, H_11_). ^13^C NMR (100 MHz) (DMSO-*d*_6_/TMS) δ ppm: 55.99 (C_2_), 66.12 (C_1_), 124.68, 126.31, 127.18, 127.97, 128.53, 128.62, 128.88, 129.00, 129.45, 129.79, 131.09, 135.14, 137.58, 147.52, 162.43 (Ar–C’s, C’s of quinolone ring, C_9_, and C_10_), 153.41 (C_3_), 166.72 (C_8_). Anal. calc. for (C_23_H_20_N_4_O_2_S): C, 66.33; H, 4.84; N, 13.45; S, 7.70; found: C, 54.00; H, 4.02; N, 9.38; S, 7.16.

## 3 Results and Discussion

**Scheme 1:**
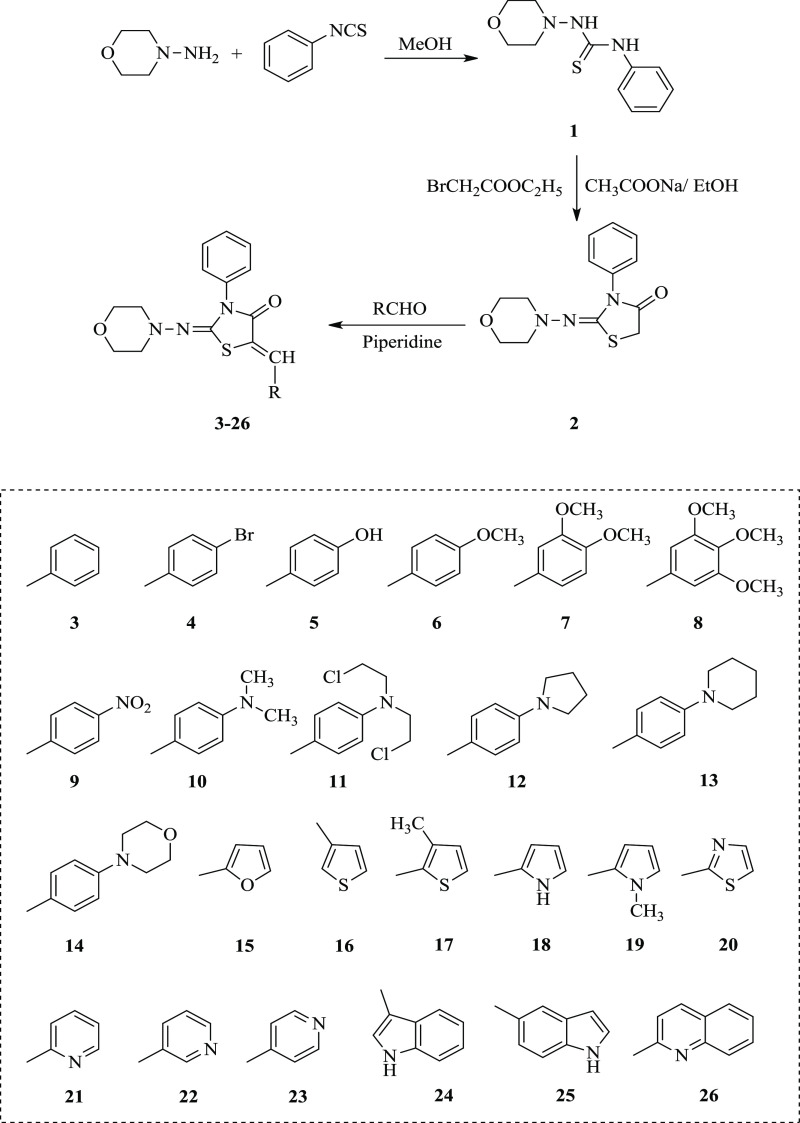
Synthetic Pathway of 5-Substituted-2-(substitute)imino-thiazolidin-4-one Derivatives (**3–26**)

This study is the first research in which the in vitro antioxidant, anticholinesterase, tyrosinase, and urease activities of synthesized compounds **1**–**26** were studied. Scheme 1 shows the synthetic pathway and substituent groups (**3–26**) carried.

The synthesized compounds were characterized by FTIR, ^1^H NMR, and ^13^C NMR. In the FTIR spectra, the expected bands of the products were observed in their respective regions. The asymmetrical and symmetrical stretching bands of ν(N–H) and ν(C=S) for thiourea **1** were observed as single at 3295, 3384, 1369, and 737 cm^–1^. In addition, the C–N stretching band was found at 1066 cm^–1^. The NH stretching bands of thiourea appeared at 3172–3456 cm^–1^,^58^ while C = S stretching bands of thiourea appeared at 1398–1488 cm^–1^ as reported earlier.^59^ The presence of N–H, C=S, and C–N stretching bands, which were characteristic bands of the synthesized thiourea **1**, showed the successful synthesis. The N–H and C=S stretching bands in the starting material disappeared after the cyclized compound **2** formed where another band related to C=O appeared at 1720 cm^–1^. The C=N stretching band of the thiazolidine was observed at 1604 cm^–1^. The C=N and C=O stretching bands belonging to the thiazolidine-4-one ring obtained from the cyclization of thiourea have been reported at 1589–1615 and 1687–1706 cm^–1^, respectively.^60^ A similar report also showed the C=N and C=O stretching bands of the 2-iminothiazolidin-4-one derivatives at 1610 and 1642 cm^–1^, respectively.^61^ The C=O, C=N, and C–N stretching bands of Knoevenagel condensation products have been reported at 1677–1720, 1581–1625, and 1040–1078 cm^–1^, respectively. The C=O and C=N stretching bands of the 2-iminothiazolidin-4-ones have been reported in the range of 1635–1798 and 1498–1625 cm^–1^, respectively.^62^ The C–N stretching bands of the 2-iminothiazolidin-4-ones were observed at 1043 cm^–1^.^63^

The corresponding protons of the products were checked in the ^1^H NMR spectra, where they appeared at their expected chemical shifts. In 1-morpholino-3-phenylthiourea (**1**), the protons attached to the nitrogen atom of thiourea (N**H**-CS-N**H**) resonated at 9.29 and 9.71 ppm. Similar −N**H** protons of thioureas have been reported between 9.75 and 10.8 ppm.^59^ In 2-(morpholinoimino)-3-phenylthiazolidin-4-one (**2**), the −N**H** proton belonging to compound **1** disappeared while the appearance of the −C**H**_2_– protons in the thiazolidine at 3.97 ppm proved the successful synthesis of the compound. The −C**H**_2_– peaks of the thiazolidine have been reported between 3.62 and 3.81 ppm.^64^ Vinylic protons (−C**H**=C−) of 5-substituted-2-(substituted)imino-thiazolidin-4-one derivatives **3**–**26** after Knoevenagel condensation of **2** with various aldehydes generally resonated between 7.28 and 8.10 ppm. As expected, the methylene protons (−C**H**_2_−) disappeared in the thiazolidine-4-one scaffold of compound **2**. The proton of the −C**H**=C– group resulting by Knoevenagel condensation has been reported around 7.42–8.12 ppm.^62^ The data above shows the successful preparation of the target compounds.

^13^C NMR analyses were also performed to verify the carbon skeleton of the synthesized compounds. The **C**=S carbons in the starting material **1** resonated at δ 177.97 ppm that disappeared in **2** while the −**C**H_2_– and **C**=O carbons of thiazolidin-4-one observed at 32.27 and 172.81 ppm, respectively. In addition, the resonance of all the carbons in the molecular structure appeared at their expected chemical shifts as reported in the literature.^65^ The carbons of 5-substituted-2-(substituted)imino-thiazolidin-4-ones (**3**–**26**) resonated around their expected chemical shifts.

**Figure 1:**
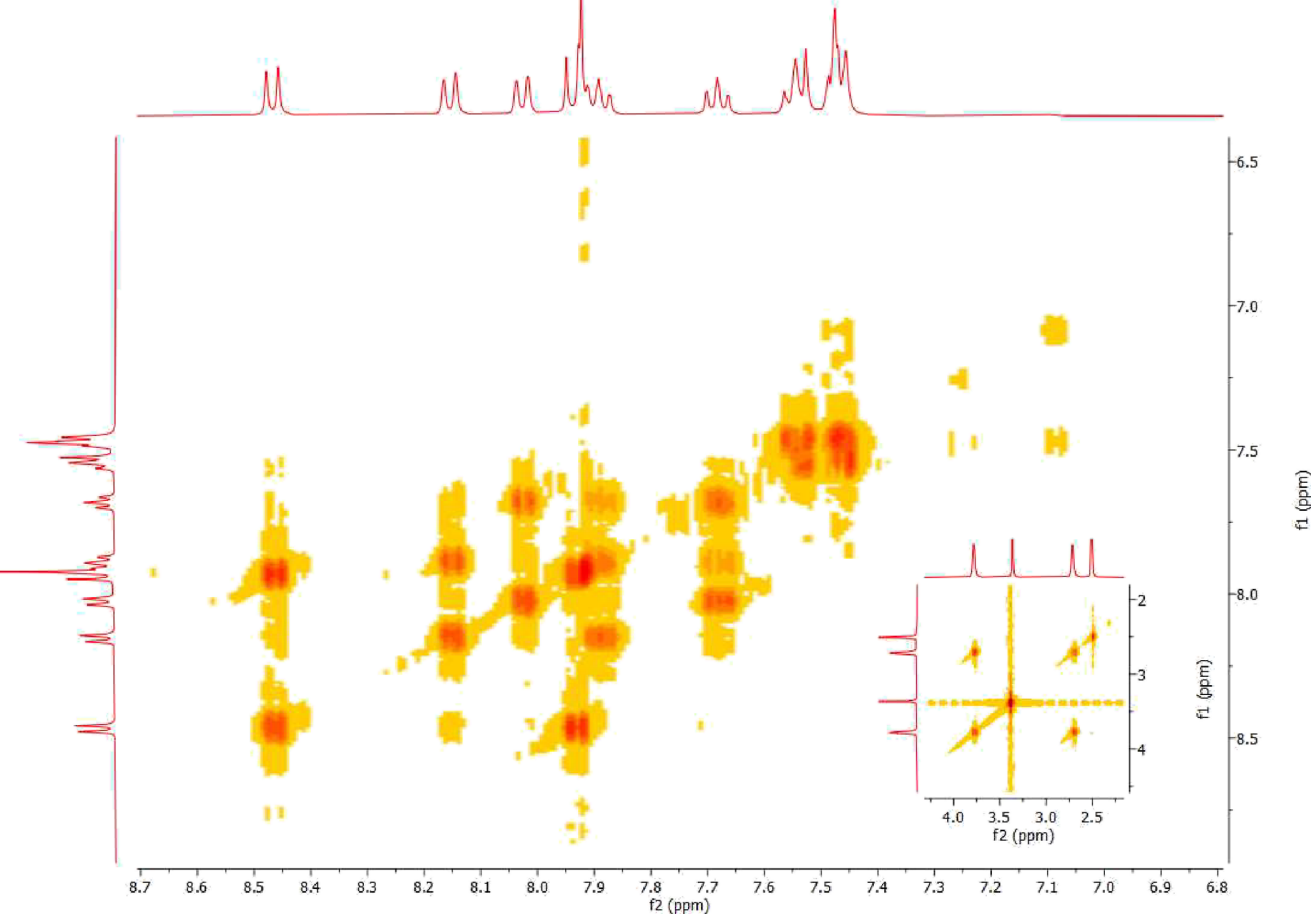
COSY spectrum of compound **26**.

To further elaborate the chemical structures of the synthesized compounds, COSY was performed to note the H–H correlation. Three spin systems were observed in the COSY spectrum (H_1_–H_2_, H_5_–H_6_, H_6_–H_7_, H_10_-H_11_, H_12_–H_13_, H_13_–H_14_, H_14_–H_15_) of **26**, which was chosen as a model compound for the 2D NMR spectrum (Figure 1).

**Figure 2:**
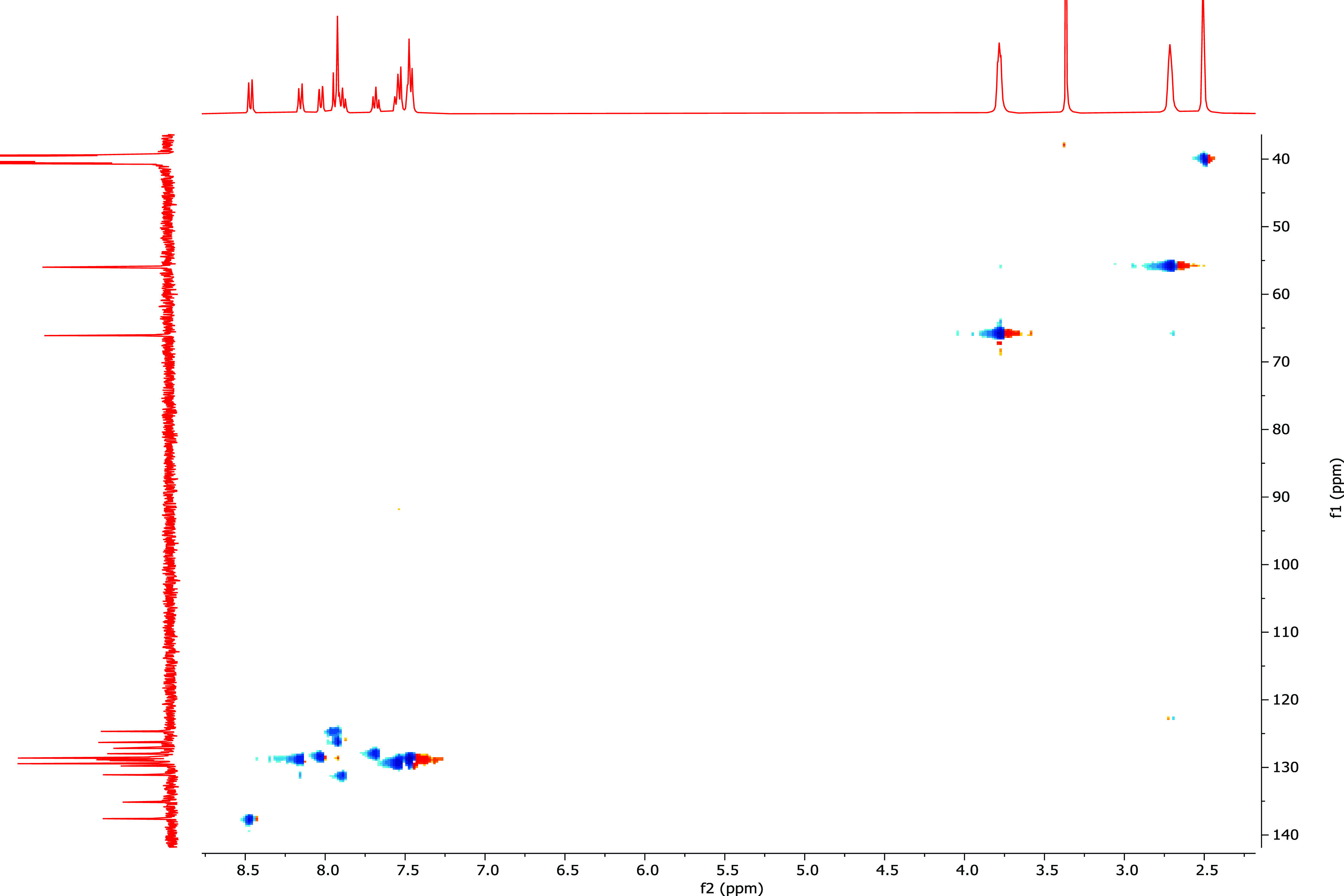
HSQC spectrum of compound **26**.

Heteronuclear single quantum correlation (HSQC) was performed to verify the presence of expected protons on corresponding carbons. In the HSQC spectrum of **26,** H_2_–C2 resonated at 2.71 and 55.99 ppm, respectively. Similarly, H_1_–C1 appeared at 3.78 and 66.12, H_6_–C_6_ appeared at 7.55 and 139.45, H_13_–C_16_ appeared at 7.68 and 127.18, H_12_–C_15_ appeared at 8.03 and 127.97, H_15_–C_18_ appeared at 8.15 and 128.62, and H_11_–C_13_ appeared at 8.47 and 137.58 ppm (Figure 2).

### Pharmacology

To proceed toward the aim of this study, the synthesized compounds were subjected to various biological activities described in the following lines.

### Antioxidant Activities

The IC_50_ values of the antioxidant activity of compounds **1**–**26** are given in Table 1. Compounds **11** (IC_50_: 11.90 ± 1.30 μM), **12** (IC_50_: 16.25 ± 0.66 μM), **13** (IC_50_: 20.20 ± 0.14 μM), and **26** (IC_50_: 22.45 ± 0.30 μM) were found most active in β-carotene-linoleic acid activity. In DPPH radical scavenging activity, all compounds (**3**–**26**) were more highly active than BHT. Compounds **11** (IC_50_: 24.60 ± 0.80 μM), **12** (IC_50_: 26.08 ± 0.44 μM), **13** (IC_50_: 29.00 ± 0.29 μM), and **26** (IC_50_: 29.70 ± 0.90 μM) were the most active in DPPH^·^ scavenging activity. In ABTS cation radical scavenging activity, **11** (IC_50_: 23.25 ± 0.34 μM), **4** (IC_50_: 23.71 ± 0.66 μM), **12** (IC_50_: 24.65 ± 0.25 μM), and **9** (IC_50_: 25.16 ± 0.37 μM) showed high activity. In the CUPRAC activity test, all compounds (**3**–**26**) were more highly active the than α-TOC standard. Compounds **10**, **16**, **12**, **4**, **9**, **13**, and **5** were determined to be notable Cu(II) reducing power.

**Table 1:** Antioxidant Activities of Compounds **1–26**^a^

**compound**	**β-carotene-linoleic acid assay IC**_**50**_**(μM)**	**DPPH^·^ assay IC**_**50**_**(μM)**	**ABTS^·+^ assay IC**_**50**_**(μM)**	**CUPRAC assay A**_**0.5**_**(μM)**
**1**	79.37 ± 0.38	253.43 ± 0.60	48.87 ± 0.78	45.18 ± 0.01
**2**	40.43 ± 0.11	101.88 ± 0.61	31.66 ± 0.13	36.35 ± 0.00
>**3**	39.78 ± 0.67	52.13 ± 0.29	31.59 ± 0.04	35.89 ± 0.01
**4**	34.70 ± 0.15	40.25 ± 0.77	23.71 ± 0.66	21.18 ± 0.00
**5**	36.21 ± 0.46	42.81 ± 0.53	28.74 ± 0.19	24.48 ± 0.02
**6**	37.07 ± 0.22	43.46 ± 0.14	30.60 ± 0.05	32.68 ± 0.01
**7**	38.24 ± 0.13	43.91 ± 0.29	31.55 ± 0.22	34.50 ± 0.01
**8**	36.45 ± 0.39	44.06 ± 0.21	32.95 ± 0.28	35.13 ± 0.03
**9**	30.44 ± 0.17	32.29 ± 0.55	25.16 ± 0.37	21.87 ± 0.01
**10**	34.23 ± 0.20	37.43 ± 0.29	30.07 ± 0.69	28.65 ± 0.00
**11**	11.90 ± 1.30	24.60 ± 0.80	23.25 ± 0.34	11.82 ± 0.00
**12**	16.25 ± 0.66	26.08 ± 0.44	24.65 ± 0.25	20.77 ± 0.03
**13**	20.20 ± 0.14	29.00 ± 0.29	27.16 ± 0.06	23.66 ± 0.02
**14**	26.31 ± 0.03	33.86 ± 0.11	30.26 ± 0.36	27.43 ± 0.00
**15**	29.73 ± 0.34	35.05 ± 0.29	32.23 ± 0.87	31.24 ± 0.00
**16**	31.40 ± 1.01	36.21 ± 0.17	35.72 ± 1.61	16.94 ± 0.01
**17**	36.01 ± 0.58	36.00 ± 1.00	33.23 ± 0.65	27.65 ± 0.01
**18**	33.62 ± 0.04	35.07 ± 0.03	31.04 ± 0.17	26.60 ± 0.02
**19**	30.82 ± 0.47	34.45 ± 0.05	29.88 ± 0.20	25.67 ± 0.00
**20**	32.79 ± 0.33	35.16 ± 0.60	31.29 ± 0.77	36.95 ± 0.03
**21**	31.51 ± 0.48	35.01 ± 0.01	30.73 ± 0.56	30.77 ± 0.00
**22**	31.62 ± 0.79	34.77 ± 0.22	30.11 ± 0.28	30.42 ± 0.01
**23**	34.60 ± 0.50	36.14 ± 0.16	32.72 ± 0.19	31.12 ± 0.00
**24**	27.73 ± 0.23	33.24 ± 0.21	29.99 ± 1.01	30.04 ± 0.02
**25**	33.61 ± 0.73	38.19 ± 0.88	34.05 ± 0.27	39.50 ± 0.01
**26**	22.45 ± 0.30	29.70 ± 0.90	26.51 ± 0.63	25.91 ± 0.02
α-TOC^b^	4.50 ± 0.09	12.26 ± 0.07	4.87 ± 0.45	40.48 ± 0.02
BHT^b^	2.34 ± 0.09	54.97 ± 0.99	2.91 ± 0.55	3.80 ± 0.02

a Values expressed are the mean ± SEM of three parallel measurements (*p* < 0.05).

b Reference compounds, BHT: butylated hydroxytoluene.

### Enzyme Inhibition Activities

The IC_50_ values of enzyme inhibition activities for compounds **1**–**26** are given in Table 2.

**Table 2:** Enzyme Inhibition Activities of Compounds **1–26**^a^

	**anticholinesterase activity**					
**compound**	**AChE IC**_**50**_**(μM)**	**BChE IC**_**50**_**(μM)**	**SI for AChE^b^**	**SI for BChE**^b^	**tyrosinase activity IC**_**50**_**(mM)**	**urease activity IC**_**50**_**(μM)**
**1**	129.95 ± 0.18	118.46 ± 0.34	0.91	1.10	35.13 ± 0.07	82.54 ± 0.29
**2**	44.30 ± 0.75	66.44 ± 0.41	1.50	0.67	30.25 ± 0.61	65.43 ± 0.67
**3**	34.22 ± 0.63	48.72 ± 0.18	1.42	0.70	29.44 ± 0.49	56.80 ± 0.15
**4**	27.65 ± 0.51	39.60 ± 0.74	1.43	0.70	28.41 ± 0.90	56.11 ± 0.22
**5**	26.99 ± 0.48	37.11 ± 0.57	1.38	0.73	28.04 ± 0.35	55.64 ± 0.81
**6**	28.95 ± 0.64	36.81 ± 0.19	1.27	0.79	27.99 ± 0.17	50.78 ± 0.08
**7**	30.45 ± 0.47	40.88 ± 0.16	1.34	0.75	27.72 ± 0.24	54.34 ± 0.62
**8**	33.61 ± 0.33	47.27 ± 0.77	1.41	0.71	25.68 ± 0.39	53.37 ± 0.64
**9**	29.80 ± 0.81	40.19 ± 0.19	1.35	0.74	25.42 ± 0.12	51.70 ± 0.12
**10**	26.48 ± 0.25	37.53 ± 0.20	1.42	0.71	25.00 ± 0.67	49.28 ± 0.55
**11**	21.45 ± 0.06	32.77 ± 0.48	1.53	0.66	23.41 ± 0.50	31.52 ± 0.49
**12**	17.41 ± 0.22	24.43 ± 0.51	1.40	0.71	24.78 ± 0.77	23.60 ± 0.70
**13**	20.05 ± 0.37	32.21 ± 0.11	1.61	0.62	26.16 ± 1.11	30.28 ± 0.95
**14**	19.21 ± 0.61	25.72 ± 1.05	1.34	0.75	8.10 ± 0.22	26.70 ± 0.52
**15**	27.40 ± 0.76	37.63 ± 0.61	1.37	0.73	5.19 ± 0.03	46.37 ± 1.08
**16**	29.04 ± 1.03	42.50 ± 0.18	1.46	0.69	24.08 ± 0.15	42.03 ± 0.81
**17**	34.76 ± 0.53	49.80 ± 1.01	1.43	0.70	3.22 ± 0.70	40.22 ± 0.12
**18**	21.44 ± 0.92	32.90 ± 0.75	1.54	0.65	16.04 ± 0.41	20.24 ± 0.77
**19**	20.59 ± 0.55	29.73 ± 0.72	1.44	0.69	19.28 ± 0.09	18.25 ± 0.50
**20**	26.84 ± 0.77	34.05 ± 0.06	1.26	0.79	21.45 ± 0.31	16.79 ± 0.19
**21**	20.65 ± 1.02	28.14 ± 0.55	1.36	0.73	11.16 ± 0.39	27.82 ± 0.51
**22**	24.78 ± 0.30	33.01 ± 0.84	1.33	0.75	9.13 ± 0.55	26.16 ± 0.47
**23**	19.73 ± 0.54	25.42 ± 0.26	1.29	0.78	8.05 ± 0.11	24.97 ± 0.54
**24**	19.52 ± 0.29	25.66 ± 0.69	1.32	0.76	7.21 ± 0.27	22.49 ± 0.11
**25**	19.80 ± 0.50	25.46 ± 0.78	1.29	0.78	8.40 ± 0.64	21.70 ± 0.06
**26**	19.84 ± 0.37	25.70 ± 0.53	1.30	0.77	8.76 ± 0.90	21.51 ± 0.44
galantamine^c^	4.48 ± 0.78	46.03 ± 0.14	10.28	0.10	NT	NT
kojic acid^c^	NT	NT			0.66 ± 0.42	NT
IQl-mimosine^c^	NT	NT			0.70 ± 0.11	NT
thiourea^c^	NT	NT			NT	24.20 ± 0.3

a Values expressed are the mean ± SEM of three parallel measurements (*p* < 0.05).

b Selectivity index for AChE: IC_50_ for BChE/IC_50_ for AChE. Selectivity index for BChE: IC_50_ for AChE/IC_50_ for BChE. NT: not tested.

c Reference compounds.

### Anticholinesterase Inhibition Activity

The anticholinesterase enzyme inhibition activity of **3**–**26** showed much better activity against AChE and BChE inhibition than starting materials **1** and **2**. The thiazolidine derivatives were less active than the galantamine standard (IC_50_: 4.48 ± 0.78 μM), where **12** (IC_50_: 17.41 ± 0.22 μM) showed the highest AChE inhibition. In the BChE inhibition activity, all compounds (**3**–**26**) except **3**, **8**, and **17** showed better activity than galantamine (IC_50_: 46.03 ± 0.14 μM). Compound **12** (IC_50_: 24.43 ± 0.51 μM) was determined to be the most active against BChE in the synthesized thiazolidine-4-one series. Interestingly, N-heterocycles showed higher anticholinesterase activity as compared to nonheterocycle. Galantamine, donepezil, and tacrine, used as standards in anticholinesterase inhibition activity, have tertiary amine functionality. In our study, *tert*-amine-containing derivatives showed higher AChE and BChE inhibition activities also.

AChE and BChE selectivity index (SI) values of all synthesized compounds are given in Table 2. According to their SI values, the syntheses showed more selectivity to AChE than BChE.

### Tyrosinase Inhibition Activities

Compounds **17** (IC_50_: 3.22 ± 0.70 mM), **15** (IC_50_: 5.19 ± 0.03 mM), **24** (IC_50_: 7.21 ± 0.27 mM), **23** (IC_50_: 8.05 ± 0.11 mM), **14** (IC_50_: 8.10 ± 0.22 mM), **25** (IC_50_: 8.40 ± 0.64 mM), **26** (IC_50_: 8.76 ± 0.90 mM), and **22** (IC_50_: 9.13 ± 0.55 mM) were identified as the most active compounds in tyrosinase inhibition activity. N-heterocycle was chosen in the rational synthesis design of target molecules based on the known current activity of l-mimosine, niaciamide, and chloroquine molecules, which have heterocyclic structure nitrogen-containing, on tyrosinase enzyme inhibition. Considering the tyrosinase enzyme inhibition activity test findings, this selection has been shown to be correct.

### Urease Inhibition Activities

Thiazole-containing **20** (IC_50_: 16.79 ± 0.19 μM) and **19** (IC_50_: 18.25 ± 0.50 μM), pyrrole-containing **18** (IC_50_: 20.24 ± 0.77 μM), quinolone-containing **26** (IC_50_: 21.51 ± 0.44 μM), indole-containing **25** (IC_50_: 21.70 ± 0.06 μM), and **24** (IC_50_: 22.49 ± 0.11 μM) were found to be more active than the positive standard thiourea (IC_50_: 24.20 ± 0.03 μM). The urease enzyme inhibition activity of selected heterocycles in the rational design of target molecules, especially inspired by the known current activity of lansoprazole, rabeprazole, and omeprazole molecules containing a N-heterocycle on urease enzyme inhibition, supported this prediction.

### Structure–Activity Relationship (SAR) Evaluated of Thiazolidine-4-one Scaffold (**3–26**)

**Figure 3:**
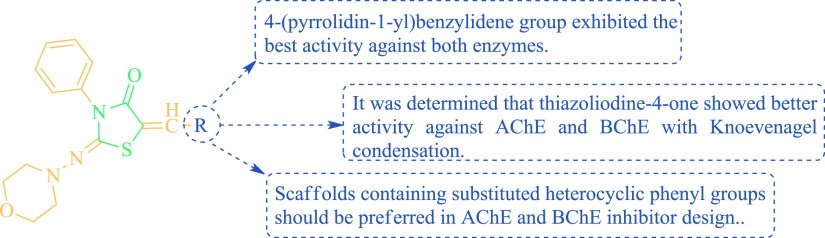
SAR of thiazolidine-4-one scaffold (**3–26**) as AChE and BChE inhibitors.

According to the anticholinesterase inhibition activity results in Table 2, when the structure–activity relationship of the synthesis (**3–26**) AChE and BChE inhibition was examined, the compound **12** attached to the 4-pyrrolidinylphenyl ring showed better activity than the structures condensed to phenyl. It was determined that increasing the number of methoxy group in the phenyl ring decreased the activity against both enzymes. The SAR evaluation of the thiazolidine-4-one scaffold (**3–26**) as inhibitors of AChE and BChE is given in Figure 3.

**Figure 4:**
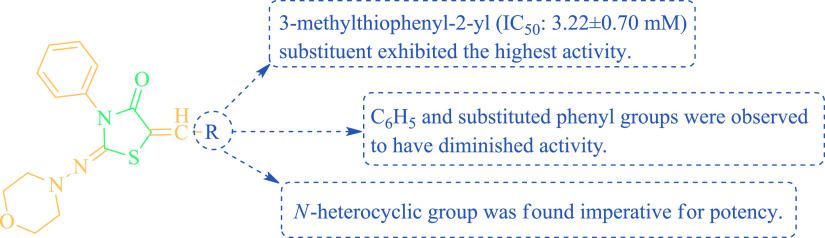
SAR finding of thiazoliodine-4-one scaffold **(3–26)** as tyrosinase inhibitors.

According to the tyrosinase inhibition activity results in Table 2, when the structure–activity relationship of synthesis **(3–26)** tyrosinase inhibition was examined, it was determined that S-heterocyclic and substituted S-heterocyclic structures inhibited tyrosinase better. It was determined that heterocyclic structures (compounds **24**, **25**, and **26**) conjugated to the phenyl ring exhibited better activity than the 4-substituted-heterocyclic structures to the phenyl ring. It was found that compound **11** (-N(CH_3_)_2,_ IC_50_: 23.41 ± 0.50 mM), one of the structures that donated electrons to the phenyl ring, was better tyrosinase inhibition than compound **4** (−Br, IC_50_: 28.41 ± 0.90 mM). The increase in the number of -OCH_3_ groups in the phenyl ring led the activity in a positive direction. The overall SAR finding of tyrosinase inhibitors of the thiazoliodin-4-one scaffold **(3–26)** is given in Figure 4.

**Figure 5:**
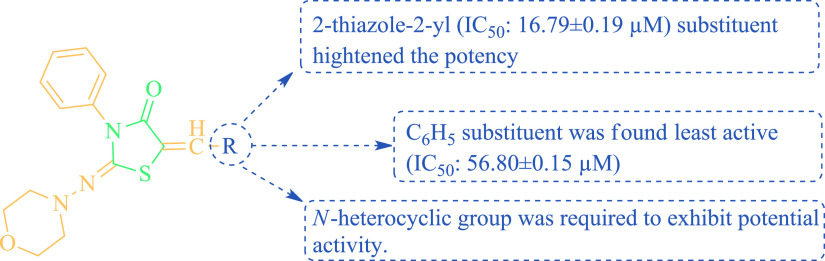
SAR evaluated as urease inhibitors of thiazoliodine-4-one scaffold (**3–26**).

The structure–activity relationship of urease inhibition of synthesis substances was examined, it was determined that the activity increased as the synthesis step progressed. According to Table 2, in the inhibitor design of the three-step target products, it was observed that the phenyl groups (**3–16**) directly attached to the thiazolidine-4-one structure showed better activity than the potential of the electron-donating groups (−N(CH_3_)_2_ > −OCH_3_ > −OH > −Br). The activity progressed as monosubstituted > trisubstituted > disubstituted as the number of bonding positions of −OCH_3_ attached to the phenyl ring within the electron donating group increased. Another important point is that when heterocyclic structures are attached instead of phenyl, it has been determined that it exhibits significant increases in activity. Compound **20** (N and S) (IC_50_: 16.79 ± 0.19 μM) containing two five-membered heteroatoms was found to exhibit more excellent activity than compound **15** (IC_50_: 46.37 ± 1.18 μM), **16** (IC_50_: 42.03 ± 0.81 μM), **17** (IC_50_: 40.22 ± 0.12 μM), **18** (IC_50_: 20.24 ± 0.77 μM), and **19** (IC_50_: 18.25 ± 0.50 μM) containing a single heteroatom. The six-membered N-heterocyclic structures compound **23** (pyridin-4-yl, IC_50_: 24.97 ± 0.54 μM) showed better activity than compound **22** (pyridin-3-yl; IC_50_: 26.16 ± 0.47 μM) and compound **21** (pyridin-2-yl; IC_50_: 27.82 ± 0.51 μM). Finally, the activity of condensed compounds (compound **24**; IC_50_: 22.49 ± 0.11 μM, compound **25**; IC_50_: 21.70 ± 0.06 μM, and compound **26**; IC_50_: 21.51 ± 0.44 μM) from heteroatom-containing structures was observed to be more active than compounds bound to phenyl (compound **12**; IC_50_: 23.60 ± 0.70 μM), compound **13** IC_50_: 30.28 ± 0.95 μM), and compound **14**; IC_50_: 26.70 ± 0.52 μM). The overall SAR assessment of urease inhibitors of the thiazolidine-4-one (**3–26**) scaffold is given in Figure 5.

### Characterization of the Binding Site of Target Enzymes with Molecular Docking

Molecular docking procedure was applied to examine the interaction mechanism and binding affinities of 5-substituted-2-(substituted)imino-thiazolidin-4-ones with AChE, BChE, tyrosinase, and urease. The lower the binding energy and *K*_i_ value, the tighter the ligand binds to the enzyme or the greater the binding affinity between the ligand and the protein. Based on the docking results, compounds **3**–**26** have showed better binding affinity against AChE, BChE, tyrosinase, and urease compared with positive standards [galantamine: AChE (−9.13 kcal/mol), galantamine: BChE (−7.61 kcal/mol), kojic acid: tyrosinase (−3.96 kcal/mol), thiourea: urease (−3.32 kcal/mol)] (see Table S1 for details).

Besides, compound **26** was determined to be the most effective (top-ranked docking score) compound with a binding energy of −10.91 kcal/mol against AChE as given in Table S1. Likewise, this compound demonstrated inhibitory activity against AChE with low concentration (IC_50_: 19.84 ± 0.37 μM) in in vitro study. This compound also showed strong binding affinity and inhibitory activity with BChE, tyrosinase, and urease (see in Tables 2 and S1). The noncovalent interactions such as hydrogen bonding and hydrophobic interactions play a key role in stabilizing energetically favored ligands at the active site of a protein structure and help improve binding affinity and drug efficacy. In this direction, we analyzed noncovalent bond interactions to better understand the binding affinity of compound **26**, which showed strong biological activity according to the in silico and in vitro analysis, with these enzymes at the molecular level.

**Figure 6:**
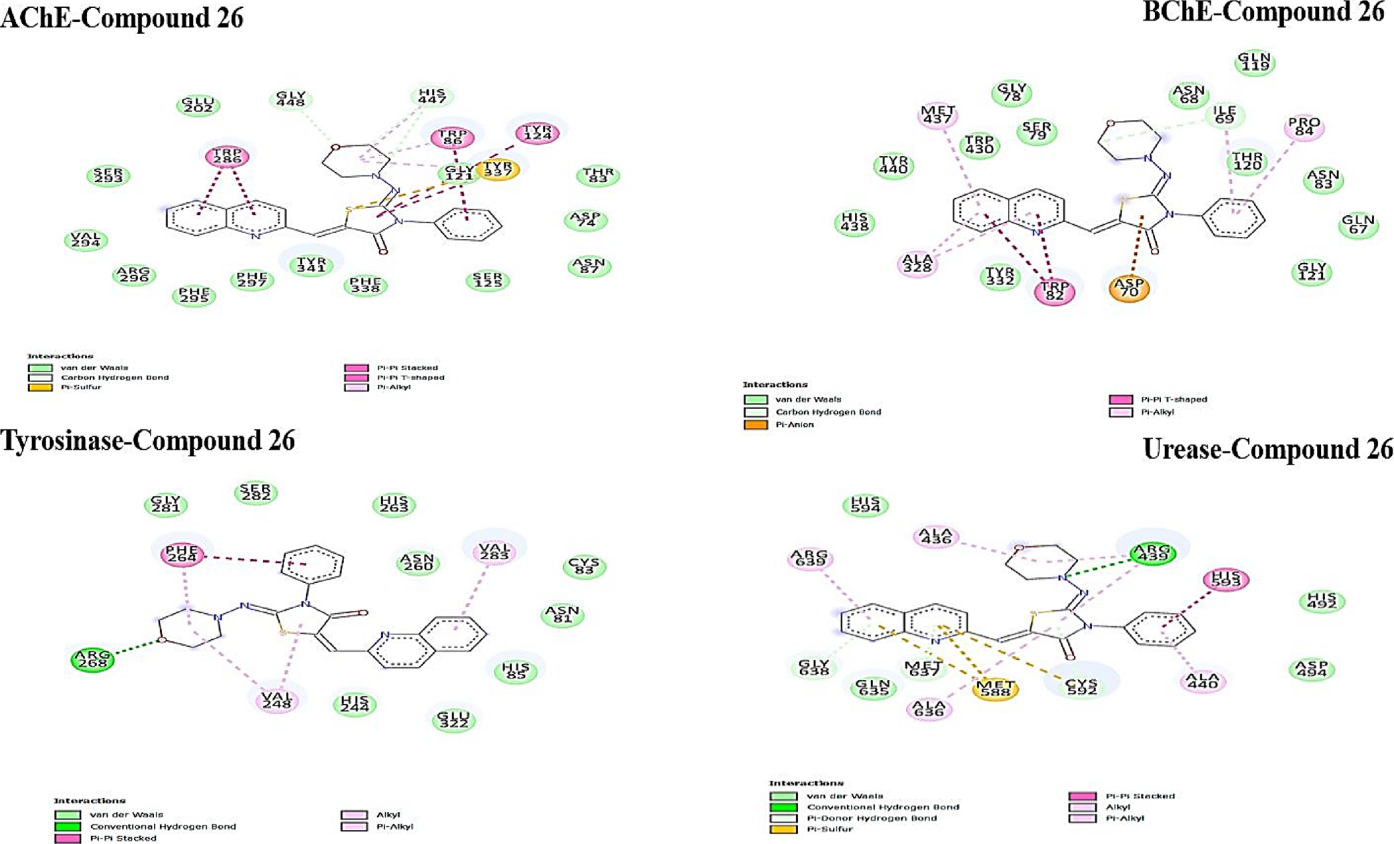
2D analysis of the lowest energy binding conformations of AChE, BChE, tyrosinase, and urease and the most effective compound **26**.

The active site of AChE contains two subsites. An esteratic subsite includes the catalytic triad consists of Ser203, His447, and Glu334 and is responsible for the catalytic functional unit of AChE. The anionic subsite consists of Trp86, Glu202, and Tyr337.^65,66^ Compound **26** formed a hydrogen bond with His447 and Gln448, pi-sulfur bond interaction between sulfur atom and Tyr337, π–π stacking interaction with Trp286, π–π T-shaped bond interaction with Tyr124 and Tyr337, and π-alkyl interaction with Trp86, as illustrated in Figure 6.

Likewise, compound **26** and galantamine interacted with Trp82, Asn83, Pro84, Thr120, Gly121, and His438 residues in the BChE enzyme, which fit well in the active site of BChE (Figure 6 and Table S1). In addition, compound **26** formed a hydrogen bond with Ile69, π-anion interaction with Asp70, and π-alkyl interaction with Met437 and Ala328 of BChE. This interaction may contribute to better binding affinity against BChE than the galantamine standard.

In the meantime, the active site of the tyrosinase enzyme contains six conserved histidine residues (His61, His85, His94, His259, His263, and His269) that are necessary for the catalytic activities and folding of tyrosinases.^67^ According to the docking simulation, compound **26** formed hydrophobic interactions with His85, His263, and His244 conserved histidine residues of the tyrosinase enzyme active site. Furthermore, compound **26** was found to make strong hydrogen bonding interactions with the Arg268 with a bond distance of 2.15 Å, which was located on the active site of tyrosinase (Figure 6).

In addition, compound **26** formed four hydrogen bonds with Arg439, Cys592, Met637, and Gly638 residues, as well as five hydrophobic interactions with His593, Ala436, Arg439, Ala639, and Ala440 residues in urease. Cys592 is required for enzymatic activity and is found on the mobile flap closing the active site of the urease enzyme.^68^ The most potent compound **26** interacted the π-donor hydrogen bond with the Cys592, which belongs to the active site flap and is essential for enzymatic activity (Figure 6). This interaction can cause a significant decrease to the catalytic activity of urease by blocking the action of a flap at the entrance of the active site channel. Consequently, molecular docking results have suggested that compound **26** bound to the active site of AChE, BChE, tyrosinase, and urease and interacted with important amino acid residues for catalytic activity.

### Molecular Dynamic Simulation and Binding Free Energy Calculation

Compound **26** with the top-ranking docking score was simulated with AChE using GROMACS 5.0.7 to better understand the ligand binding site interaction and the stability of the protein–ligand complexes. The MM/PBSA method was used to calculate the binding energy of the complex structure using ensembles derived from molecular dynamics (MD) simulation. The binding (−120.082), van der Waals (−192.715), electrostatic (−26.430), polar solvation (121.262), and solvent accessible surface area (SASA) (−22.199 kcal/mol) energies were obtained for the AChE and compound **26**.

Intermolecular interactions play a key role in stabilizing energetically preferred ligands at the active site of a protein structure. Compound **26** occurred several intermolecular interactions with the AChE binding site. Electrostatic and van der Waals energy contributions were found to be important in interactions with Thr75, Gly121, Trp86, Trp439, and His447 residues. Especially, pi-alkyl interaction with His447 and Tyr86 was stable in the complex structure during the 100 ns MD simulation. Besides, two new strong hydrogen bond interactions occurred with Gly121 and Tyr75.

**Figure 7:**
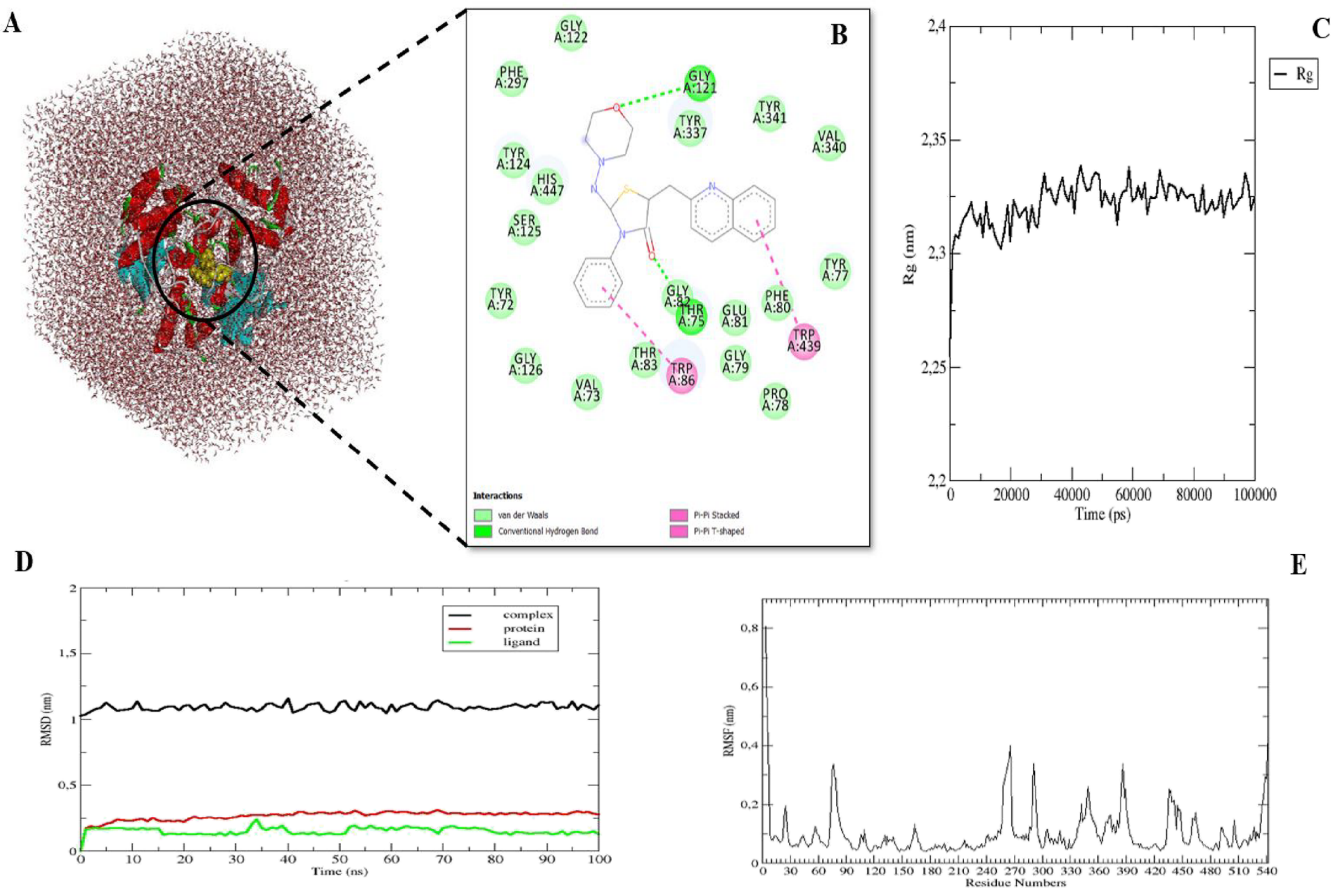
(A) 3D structure of AChE-compound **26** in the solvation box. (B) 2D analysis of the MD simulation conformations of AChE-compound **26.** (C) Radius of gyration (*R*_g_) analysis of in the complex structure during the 100 ns MD simulation time. (D) The RMSD trajectory of protein, compound **26**, and complex structures. Red color indicates protein, black color indicates complex (protein and ligand), and green color indicates ligand (compound **26**) structures. (E) The RMSF profile of AChE in the complex structures.

Furthermore, root-mean-square fluctuation (RMSF) for each residue of AChE with compound **26** was analyzed (Figure 7). RMSF reflects the mobility of a particular residue around its reference position. The result of this analysis indicated that the Ser203, His447, and Glu334 residues responsible for the catalytic functional domain of AChE have less fluctuation (0.08, 0.18, and 0.04 nm, respectively) (Figure 7). In summary, the reference position of the AChE active site residues did not significantly change on binding of the ligands, thus implying that this complex form was stable. Besides, the RMSD values of backbone Cα atoms were calculated to be in the range of 1–1.25 Å for a complex and 0.1–0.5 Å for protein and ligand structures (Figure 7). The overall RMSD results showed that there was no significant variation in the RMSD values of the AChE-compound **26** complex and protein and ligand structure. These results implied that the binding of these compound **26** at the active site of AChE was stable.

In addition, we examined the *R*_g_ for proteins in the molecular dynamic simulation data. The *R*_g_ provides information about regular secondary structure of protein and its compactness. We observed that the *R*_g_ of the protein fluctuates around stable values (2.25–2.35 nm) during the 100 ns. This analysis showed that the protein remained stable in its compact form (Figure 7).

Consequently, the MD simulation analysis results convince that the interactions of compound **26** with the amino acid, which plays an important role in the catalytic activity of the protein, remain stable throughout the simulation, so that the ligand stability is reliable in the AChE sites.

## 4 Conclusions

Thiazolidin-4-one derivatives presented as antioxidants with AChE, BChE, tyrosinase, and urease inhibitors were exhibited tremendous activity according to the bioactivity findings. Compound **11** was found to be the most active in the synthesis series (**3–26**) in all antioxidant assays. In both AChE and BChE assays, it was determined that the target products showed the best activity with the IC_50_ values of compounds **12**, 17.41 ± 0.22 and 24.43 ± 0.51 μM, respectively. In addition, all thiazolidin-4-one derivatives except compounds **3** and **8** were found to be more effective in the BChE assay than galantamine, the positive standard of the test. In the urease inhibition assay, compounds **20** (IC_50_: 16.79 ± 0.19 μM), **19** (IC_50_: 18.25 ± 0.50 μM), **26** (IC_50_: 21.51 ± 0.44 μM), **25** (IC_50_: 21.70 ± 0.06 μM), **24** (IC_50_: 23.60 ± 0.70 μM), and **12** (IC_50_: 24.20 ± 0.30 μM) were found to be more active than thiourea (IC_50_: 24.20 ± 0.30 μM), which is the positive standard of the assay), while compounds **17** (IC_50_: 3.22 ± 0.22 mM) and **15** (IC_50_: 5.19 ± 0.03 mM) were found to be the most active compounds of the series in the tyrosinase inhibition activity assay. Overall, according to the enzyme inhibition activity results, it appears to be one of the main design strategies in thiazolidin-4-one-based inhibitor development, especially N-heterocyclic scaffolds, to produce safe and efficient agents for future medical or industrial applications. Based on the biological activity findings, two or more pharmacophoric moieties of different bioactive molecules were found to exhibit significant activity in a single scaffold to obtain hybrids with improved affinity and efficacy. An SI value of ≤0.5 exhibits low selectivity, while a selectivity with an SI value of 0.5–2.0 includes compounds that inhibit both enzymes in a balanced manner. According to the therapeutic index analogy, if the SI value is >10, it creates a toxic effect.^69^ According to SI values, all synthesis products inhibit both enzymes in a balanced way. Also, considering the AChE and BChE selectivity values of the synthesis substances, none of them is considered to have a toxic effect. The SAR study of the enzyme inhibition activities of synthesis reveals the important influence of the spatial and electronic nature of their inhibition potential, providing important information for a logical pathway to inhibitor design studies of these enzymes in future studies.

## Supporting Information Available

Lowest binding energy values of the compounds **1**–**26** and positive control compounds from each docking analysis in the active site of AChE, BChE, tyrosinase, and urease; FT-IR spectrum, ^1^H NMR spectrum, and ^13^C NMR spectrum of compounds **1**–**26** (PDF)
