# Supplementary material for: Design, Synthesis, Pharmacological Activities, Structure–Activity Relationship, and In Silico Studies of Novel 5-Substituted-2-(morpholinoimino)-thiazolidin-4-ones
Source: ACS Omega. 2023 Oct 4;8(41):38641–57. doi: 10.1021/acsomega.3c05928 (PMC10586451; doi:10.1021/acsomega.3c05928)
Supplement: Supplementary file 1 — ao3c05928_si_001.pdf [file ao3c05928_si_001.pdf]

SUPPLEMENTARY DATA

Title: Design, Synthesis, Pharmacological Activities, SAR and In silico Studies of Novel 5-substitue-2-(morpholinoimino)-thiazolidin-4-ones

Yusuf Sıcak

Department of Medicinal and Aromatic Plants, Köyceğiz Vocational School, Muğla Sıtkı Koçman University, Muğla, Turkey

🖂 **Yusuf SICAK**

Department of Medicinal and Aromatic Plants, Köyceğiz Vocational School, Muğla Sıtkı Koçman University, Muğla, Turkey

E-mail: yusufsicak@mu.edu.tr

Table S1: The lowest binding energy values of the compound 1-26 and positive control compounds from each docking analysis in the active site of AChE, BChE, Tyrosinase and Urease

Compound Name	AChE Binding Energy(kcal/mol)	BChE Binding Energy(kcal/mol)	Tyrosinase Binding Energy(kcal/mol)	Urease Binding Energy(kcal/mol)
**1**	-6.78	-6.32	-5.59	-5.98
**2**	-7.01	-6.26	-5.71	-5.71
**3**	-9.33	-8.54	-6.32	-7.15
**4**	-10.67	-8.91	-7.04	-8.05
**5**	-9.73	-8.64	-6.77	-7.39
**6**	-9.38	-8.43	-6.48	-7.66
**7**	-10.14	-8.36	-6.52	-8.25
**8**	-9.75	-8.49	-6.66	-8.09
**9**	-10.83	-9.05	-7.09	-8.94
**10**	-9.96	-9.56	-7.27	-8.93
**11**	-9.83	-8.04	-6.69	-7.30
**12**	-10.76	-7.95	-6.42	-7.35
**13**	-10.25	-8.63	-7.48	-7.57
**14**	-9.74	-8.86	-7.18	-7.32
**15**	-9.85	-9.50	-7.14	-8.30
**16**	-9.67	-8.48	-6.34	-6.85
**17**	-10.27	-8.67	-7.09	-6.96
**18**	-9.37	-8.51	-5.95	-6.78
**19**	-9.75	-8.35	-6.69	-6.63
**20**	-9.56	-8.15	-6.24	-6.91
**21**	-9.75	-8.38	-6.22	-7.18
**22**	-9.72	-8.69	-6.44	-7.16
**23**	-9.70	-8.22	-6.14	-7.17
**24**	-10.61	-9.84	-6.52	-7.78
**25**	-10.30	-9.39	-6.74	-8.40
**26**	-10.91	-9.54	-6.96	-8.06
**Galantamine**	-9.13	-7.61	NT	NT
**Kojic acid**	NT	NT	-3.96	NT
**Thiourea**	NT	NT	NT	-3.32

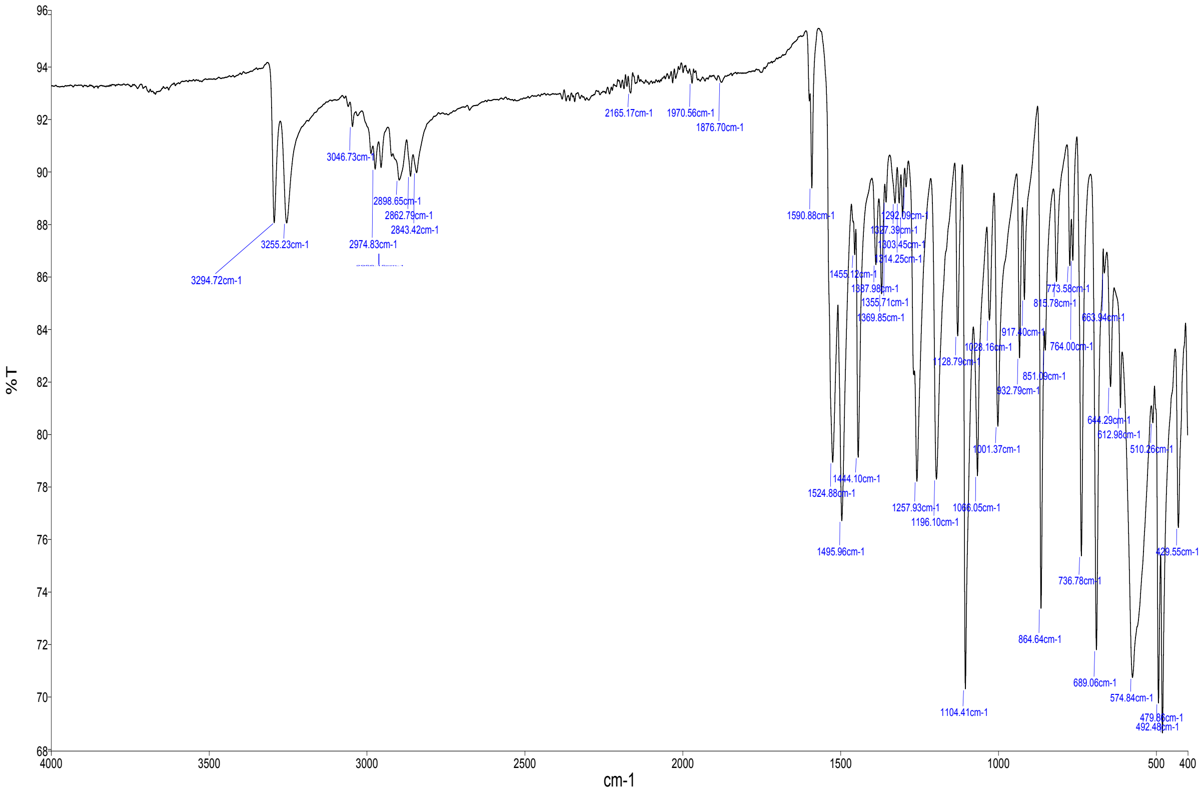

Figure S1. FT-IR spectrum of compound **1**

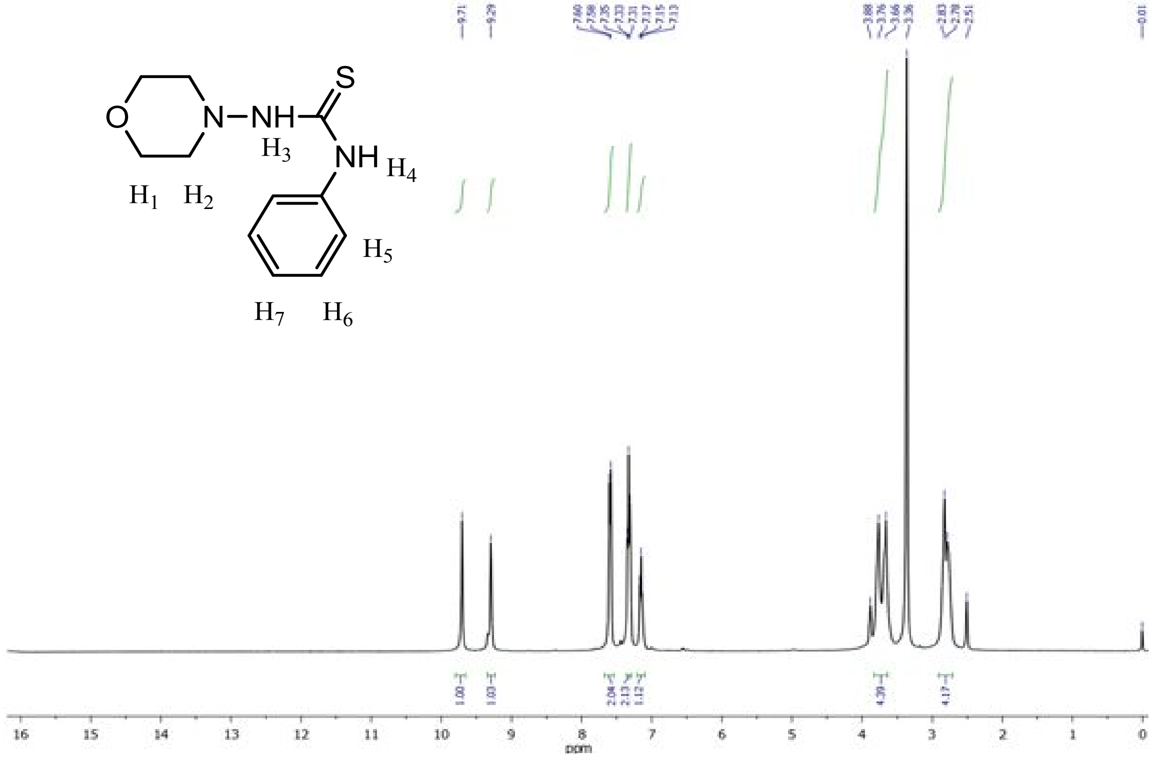

Figure S2. ^{1}H NMR spectrum of compound **1**

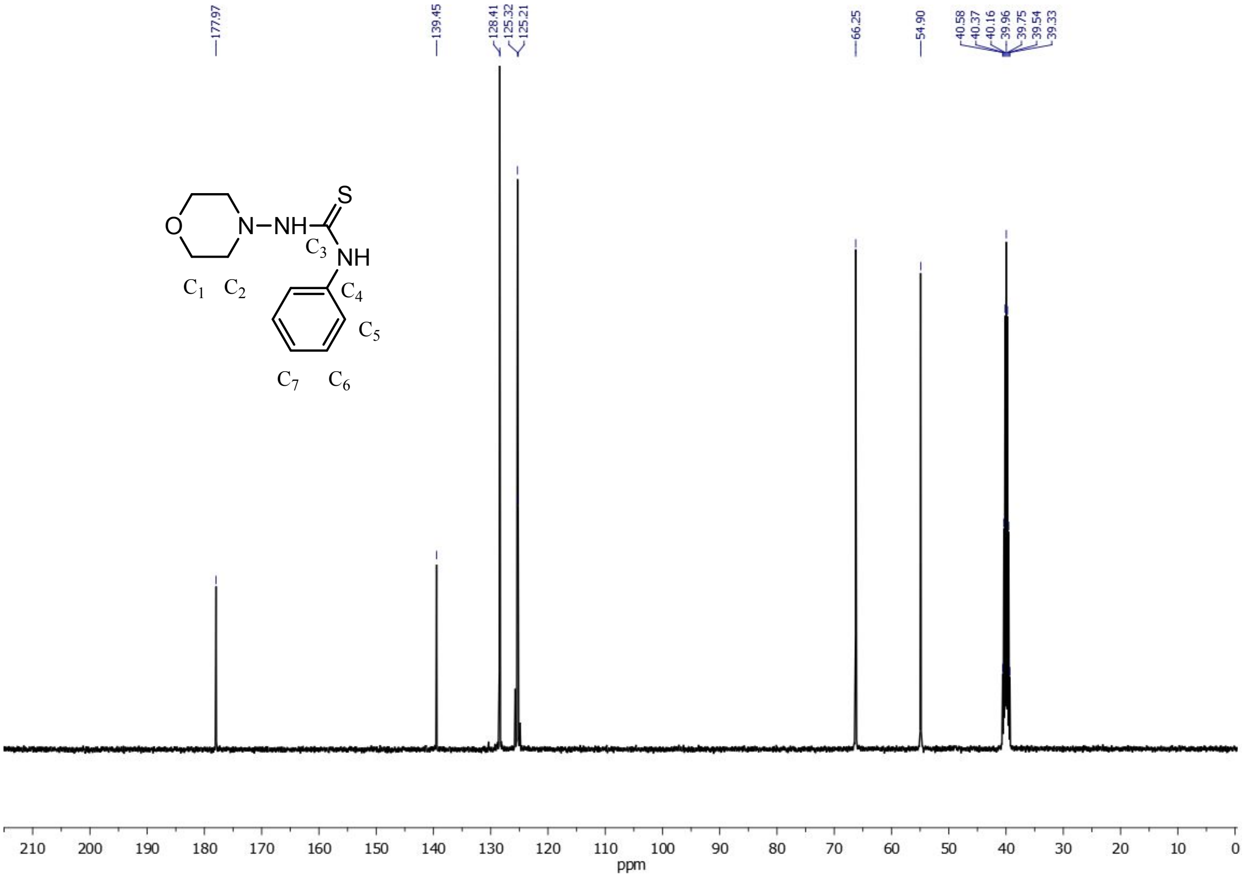

Figure S3. ^{13}C NMR spectrum of compound **1**

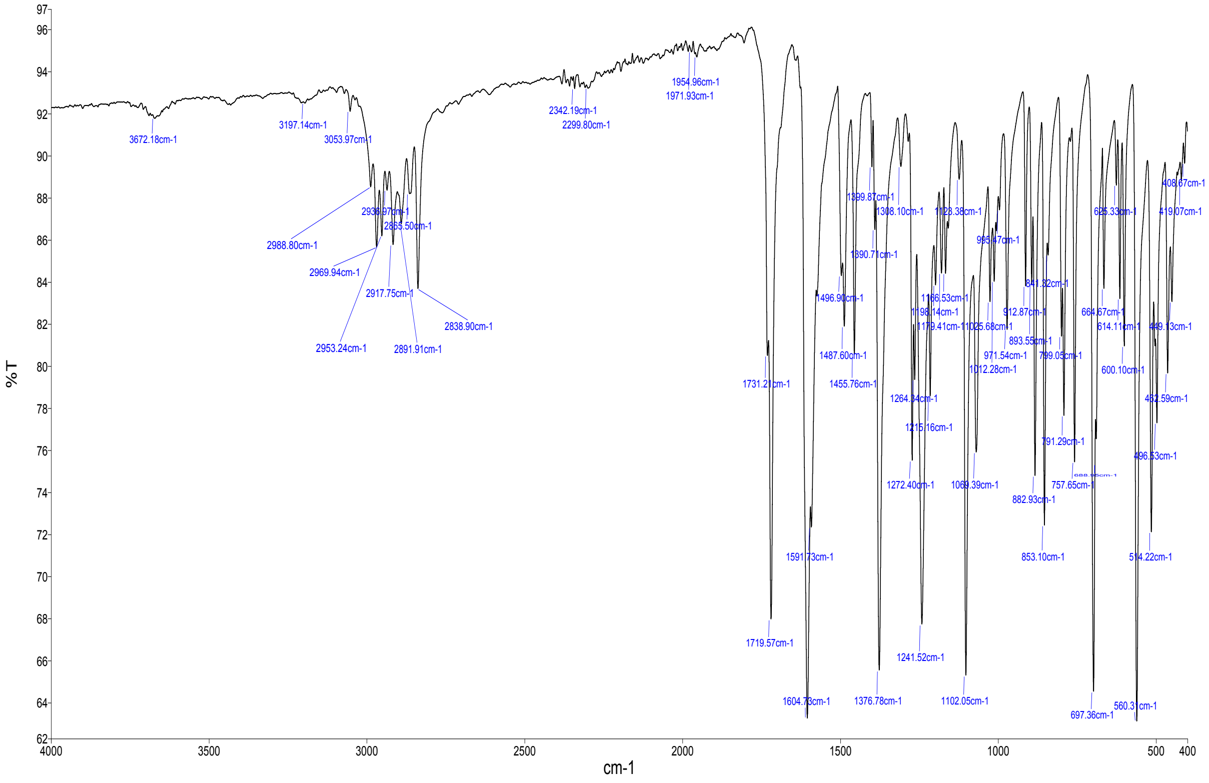

Figure S4. FT-IR spectrum of compound **2**

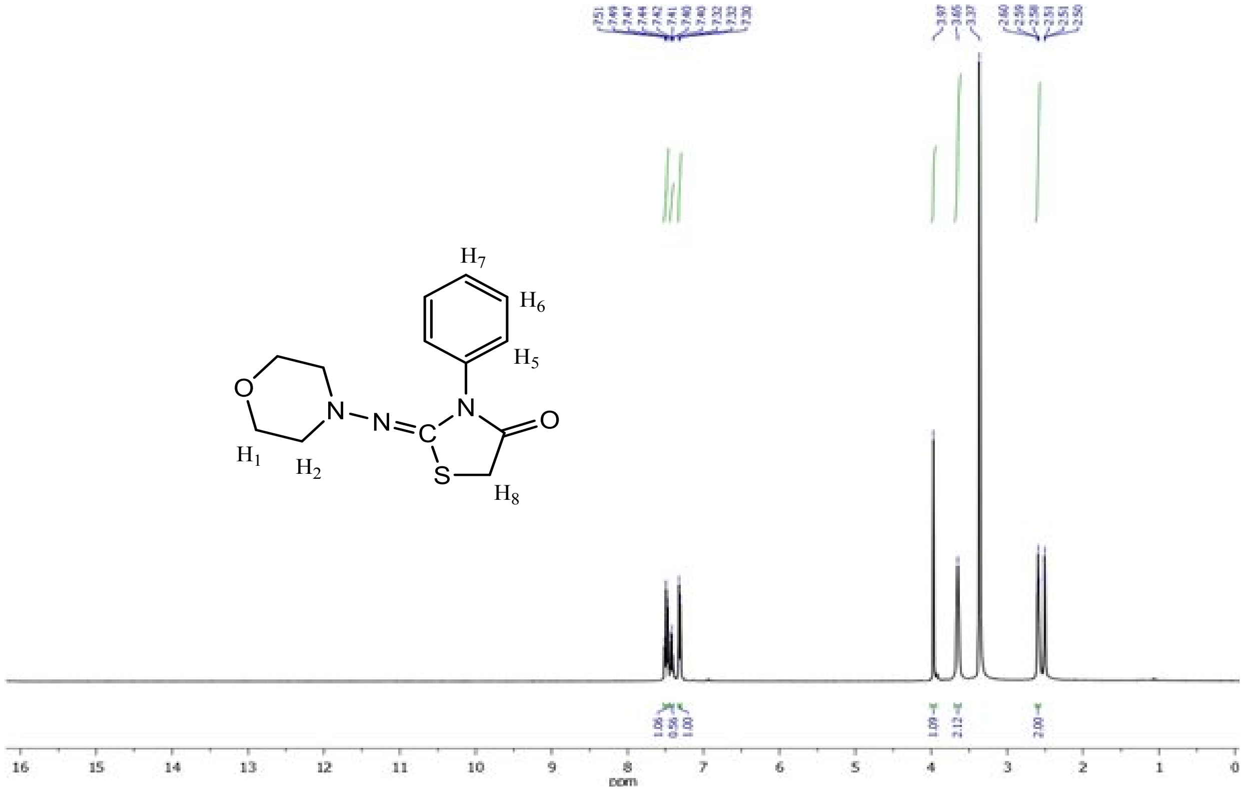

Figure S5. ^{1}H NMR spectrum of compound **2**

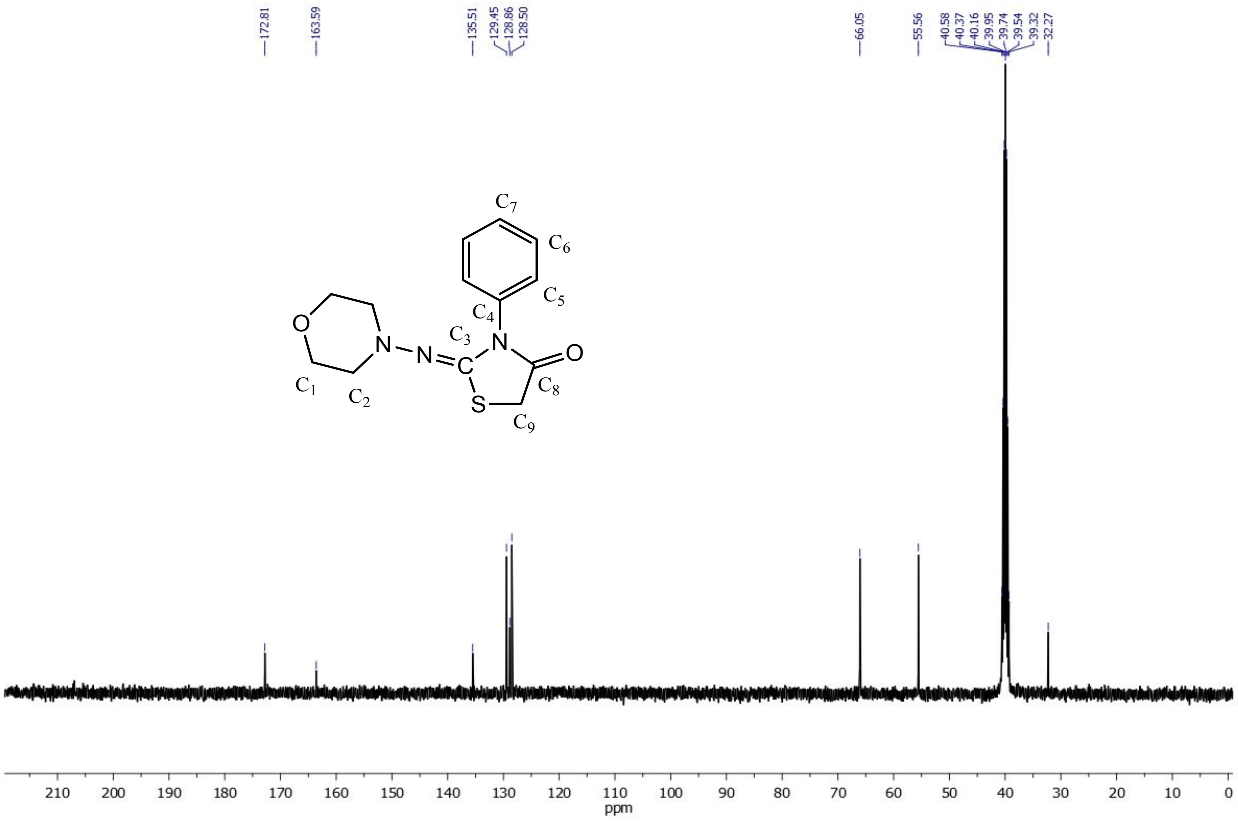

Figure S6. ^{13}C NMR spectrum of compound **2**

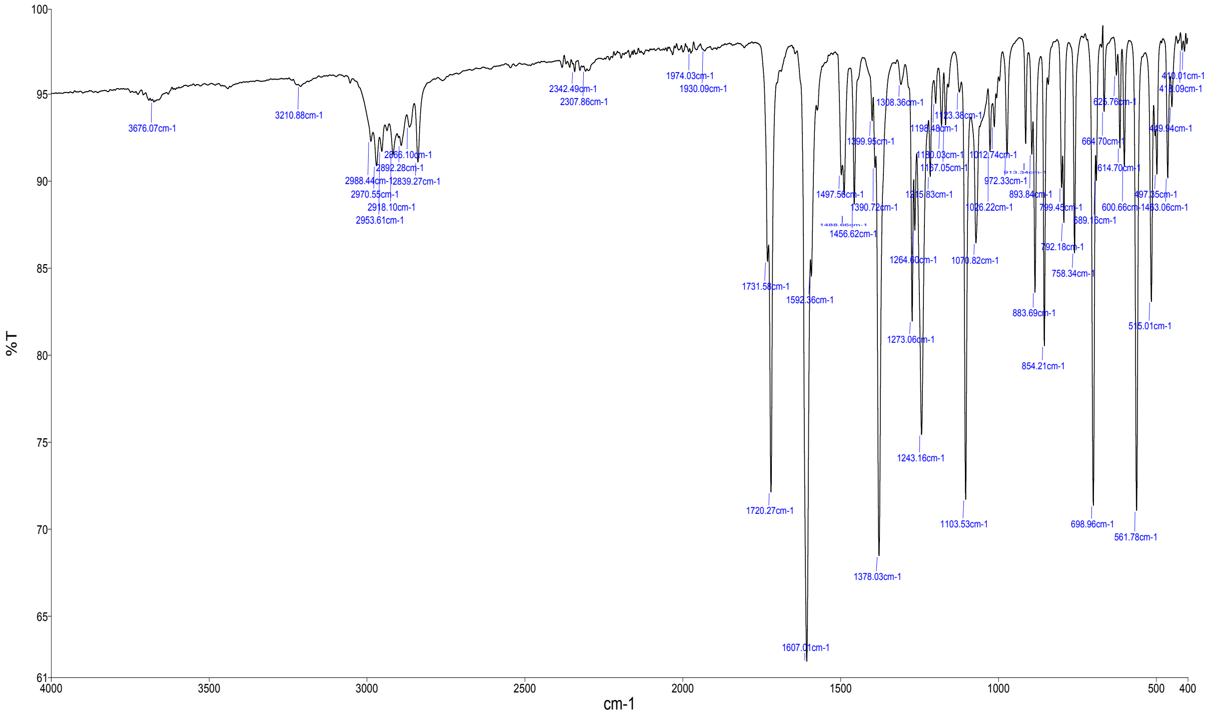

Figure S7. FT-IR spectrum of compound **3**

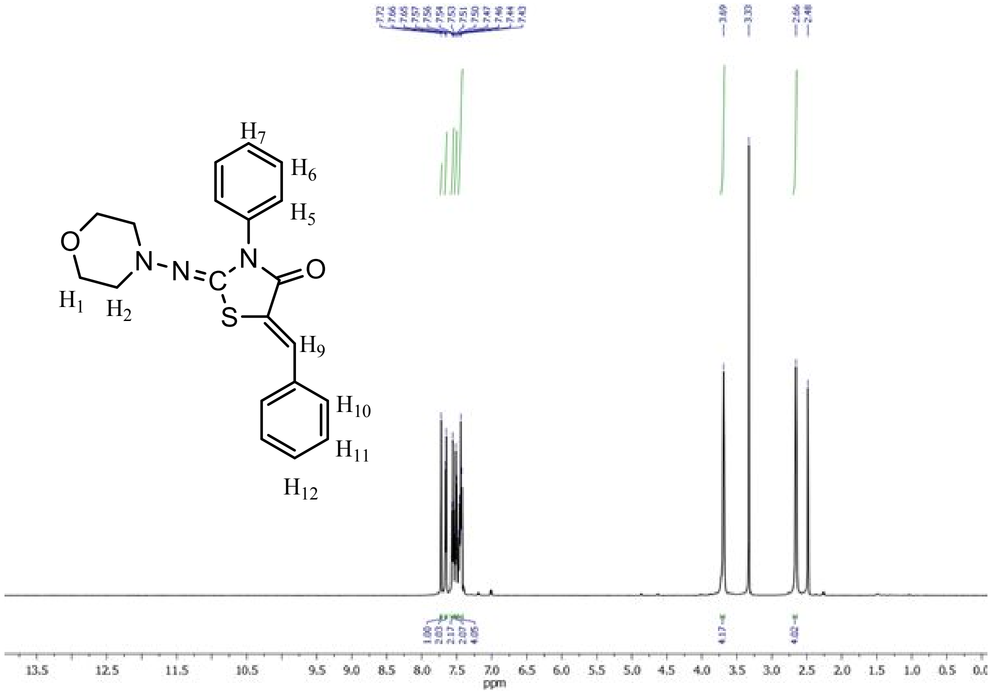

Figure S8. ^{1}H NMR spectrum of compound **3**

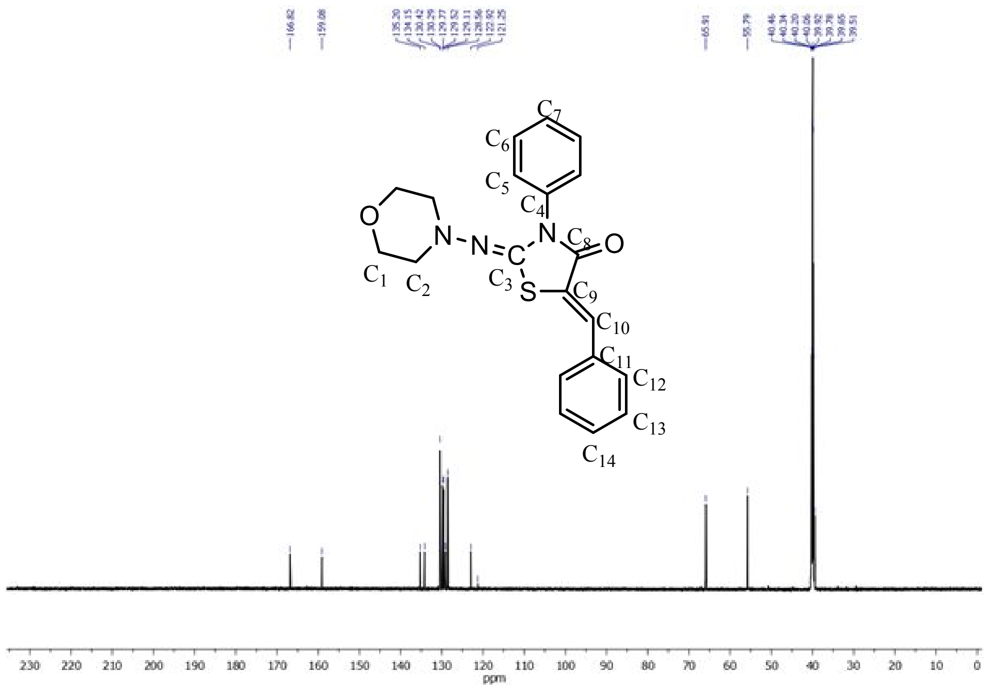

Figure S9. ^{13}C NMR spectrum of compound **3**

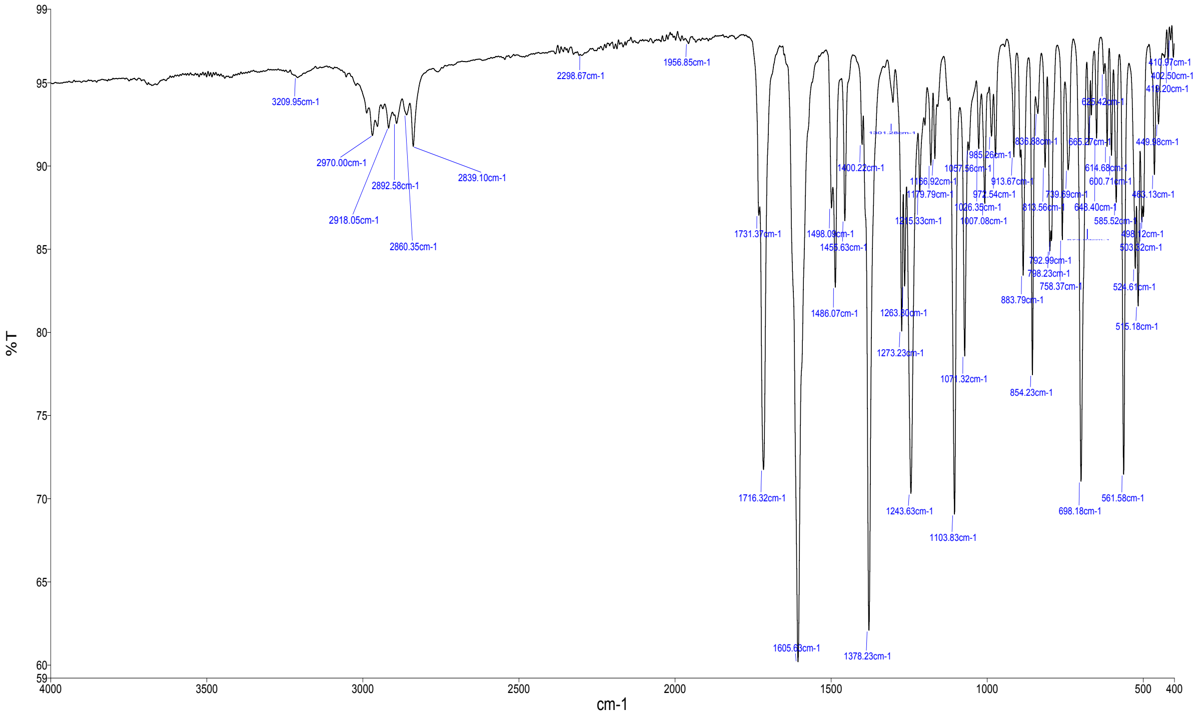

Figure S10. FT-IR spectrum of compound **4**

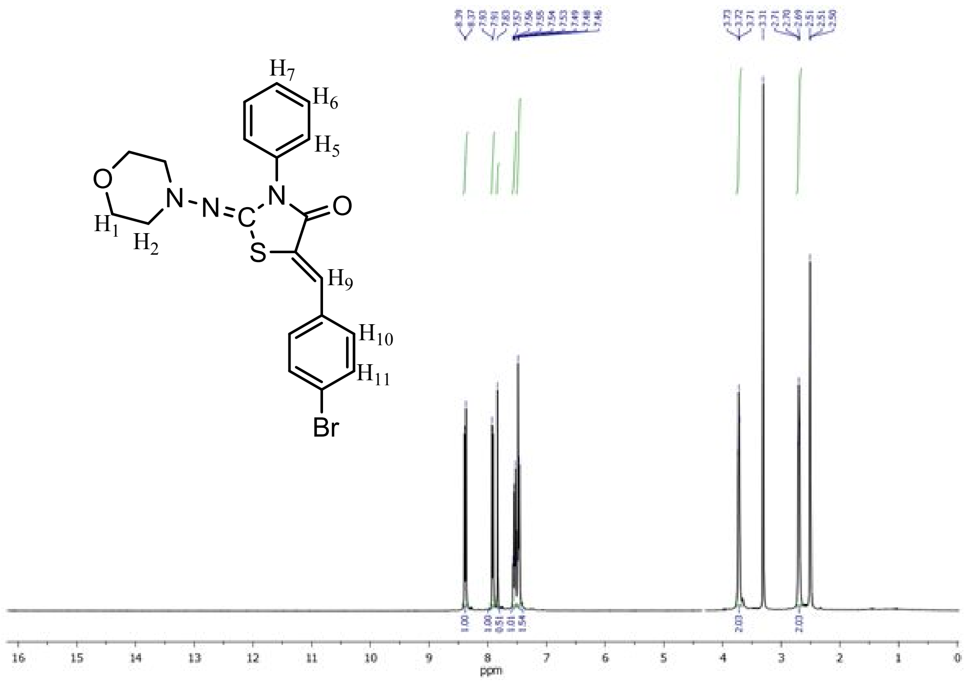

Figure S11. ^{1}H NMR spectrum of compound **4**

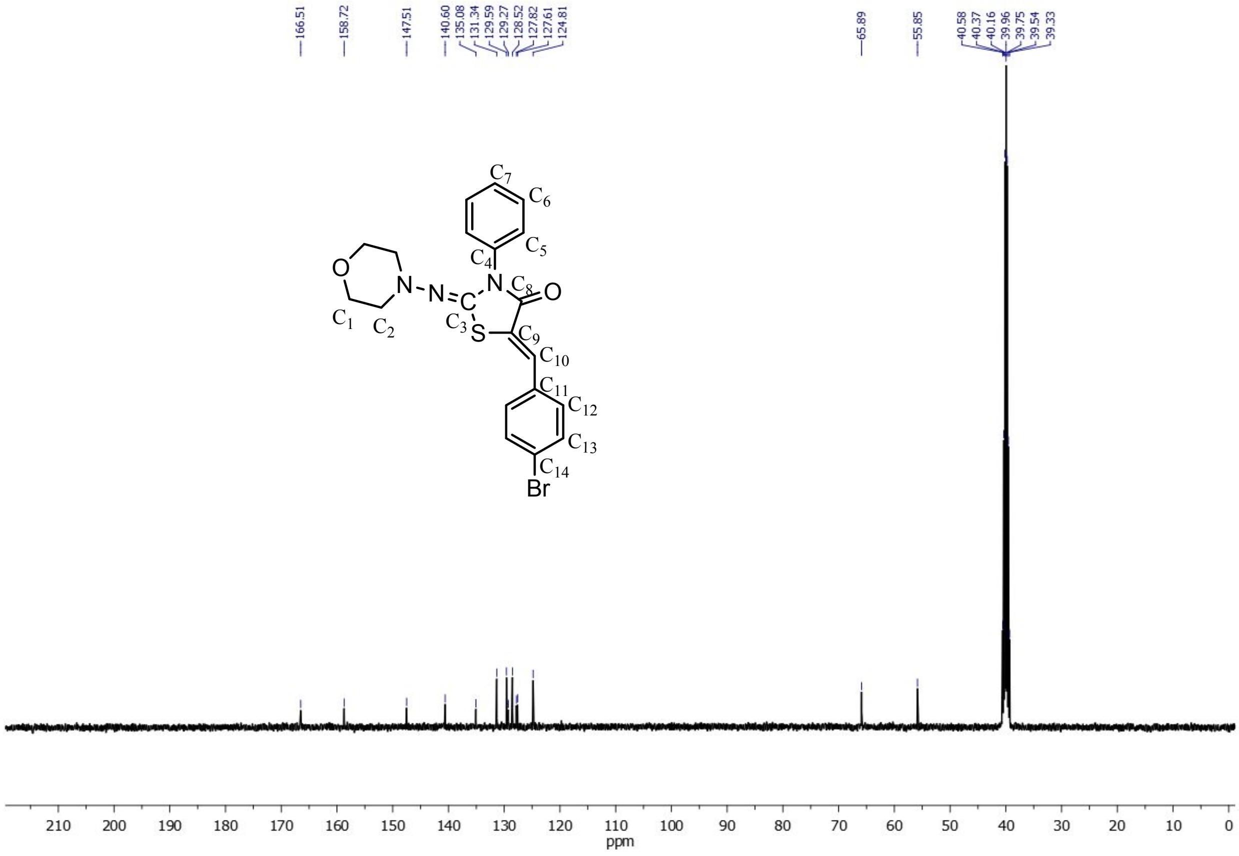

Figure S12. ^{13}C NMR spectrum of compound **4**

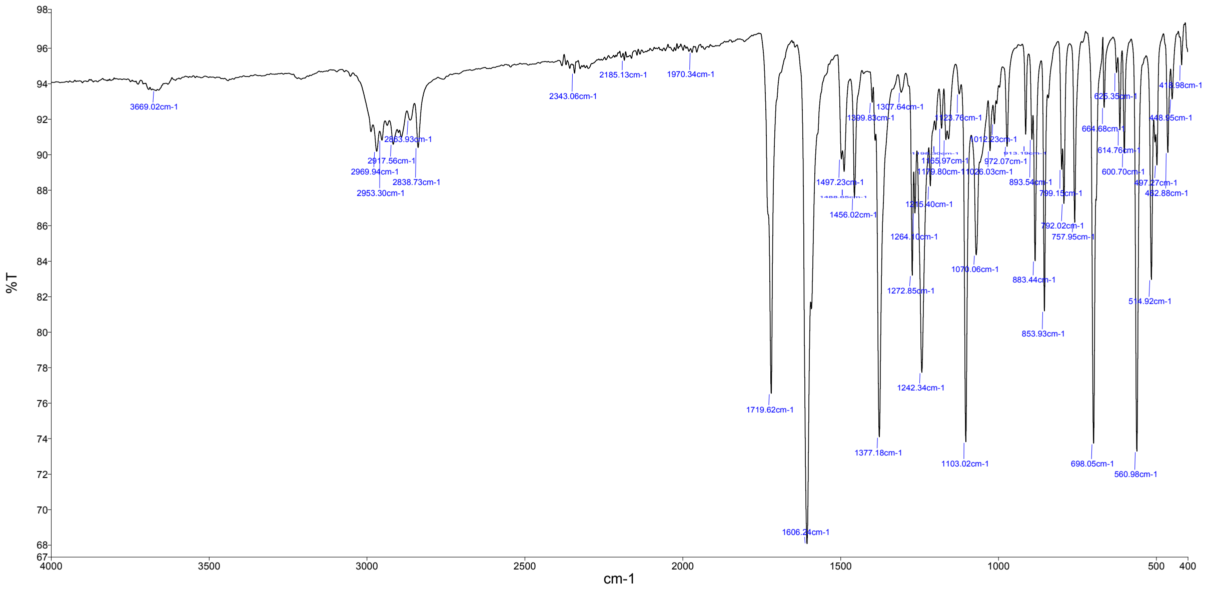

Figure S13. FT-IR spectrum of compound **5**

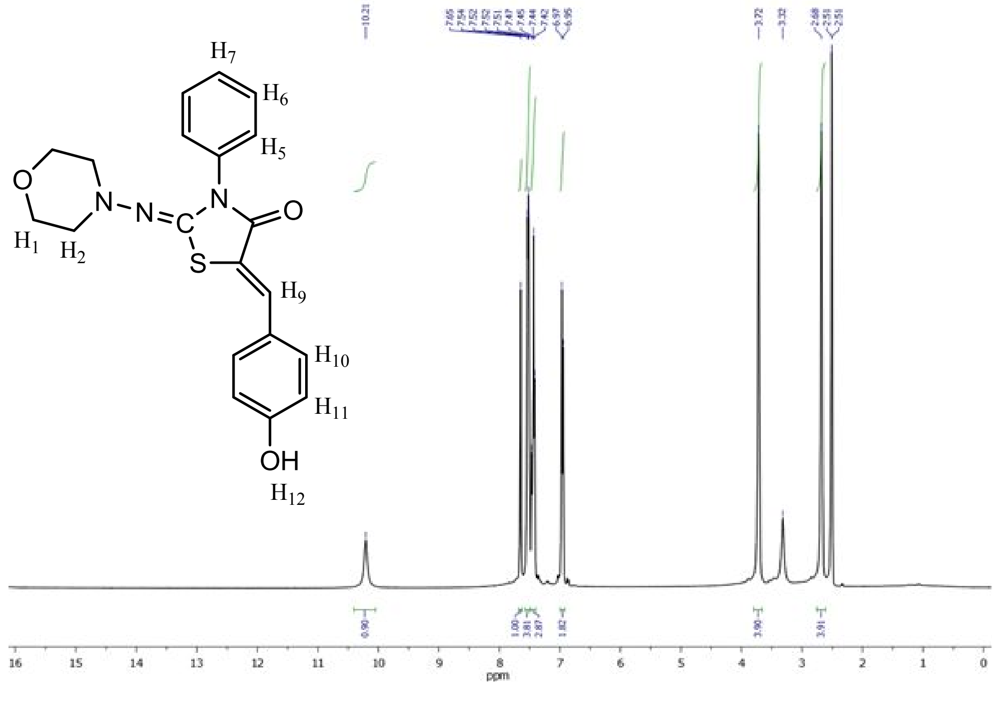

Figure S14. ^{1}H NMR spectrum of compound **5**

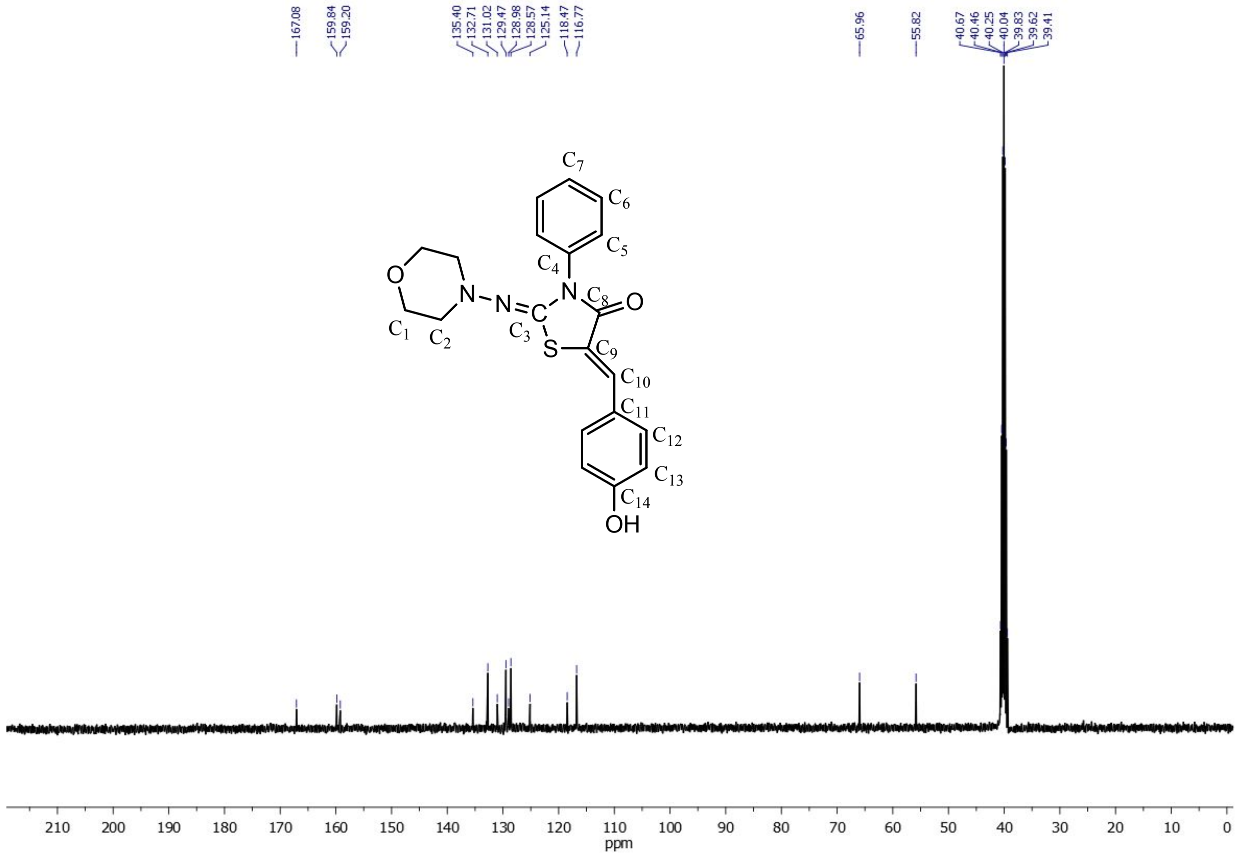

Figure S15. ^{13}C NMR spectrum of compound **5**

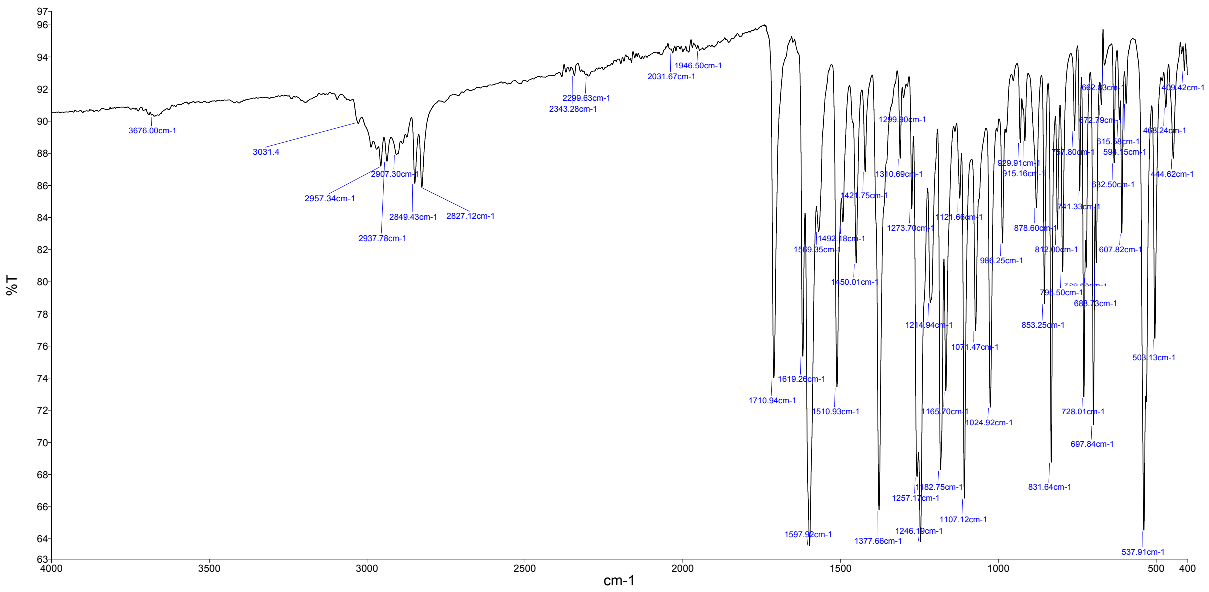

Figure S16. FT-IR spectrum of compound **6**

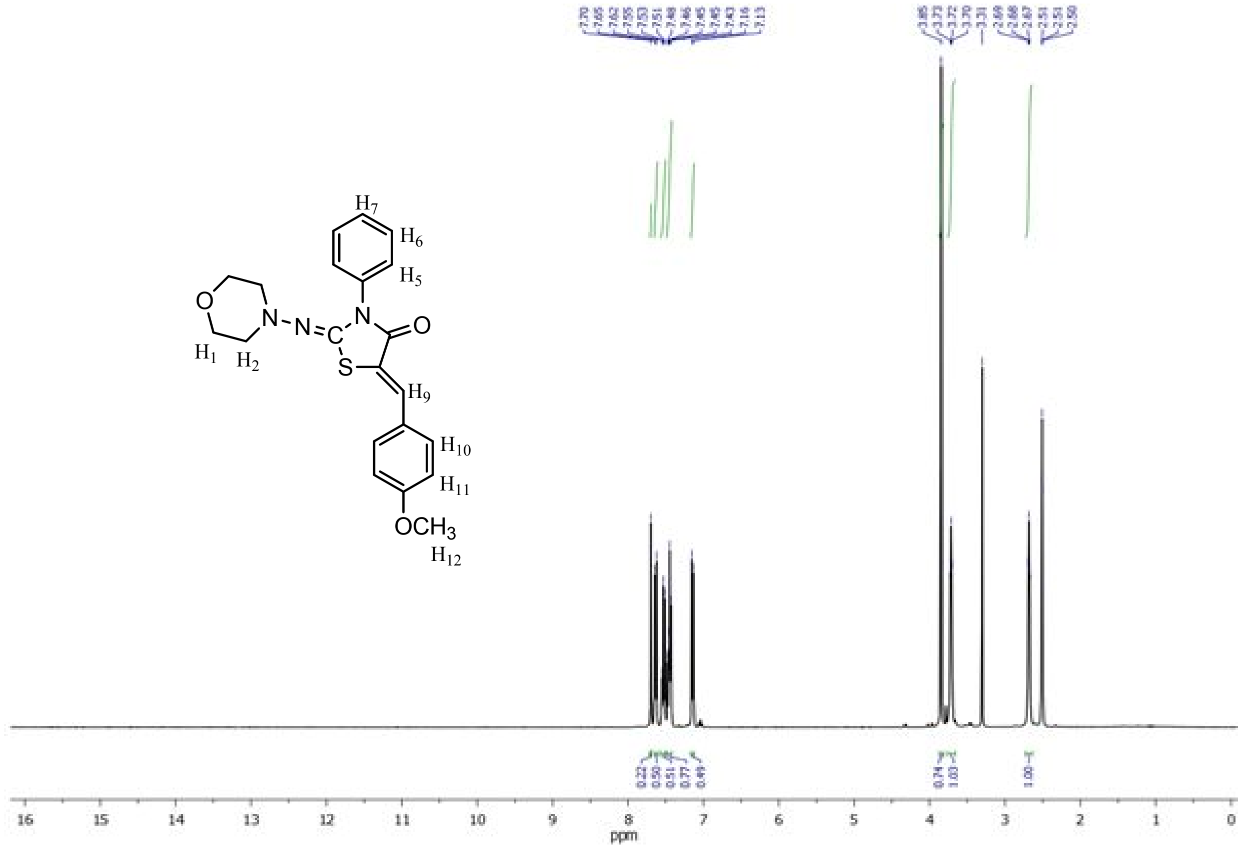

Figure S17. ^{1}H NMR spectrum of compound **6**

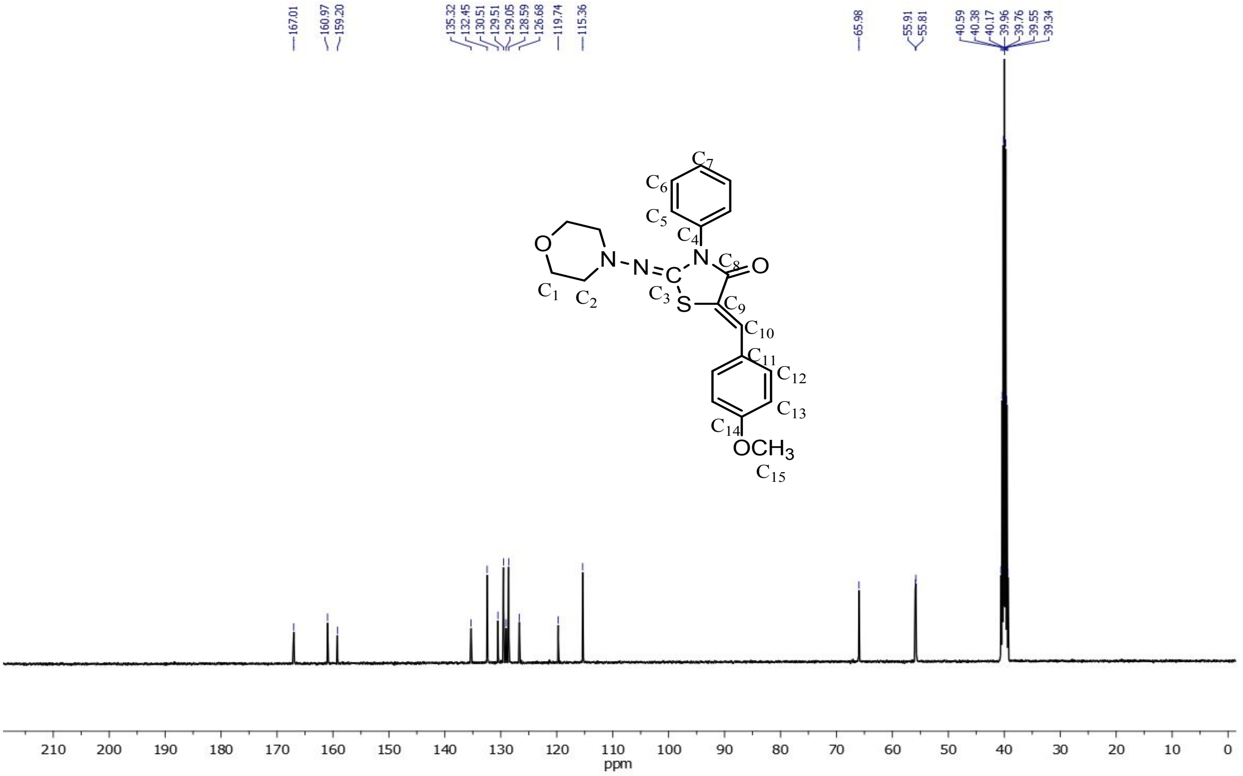

Figure S18. ^{13}C NMR spectrum of compound **6**

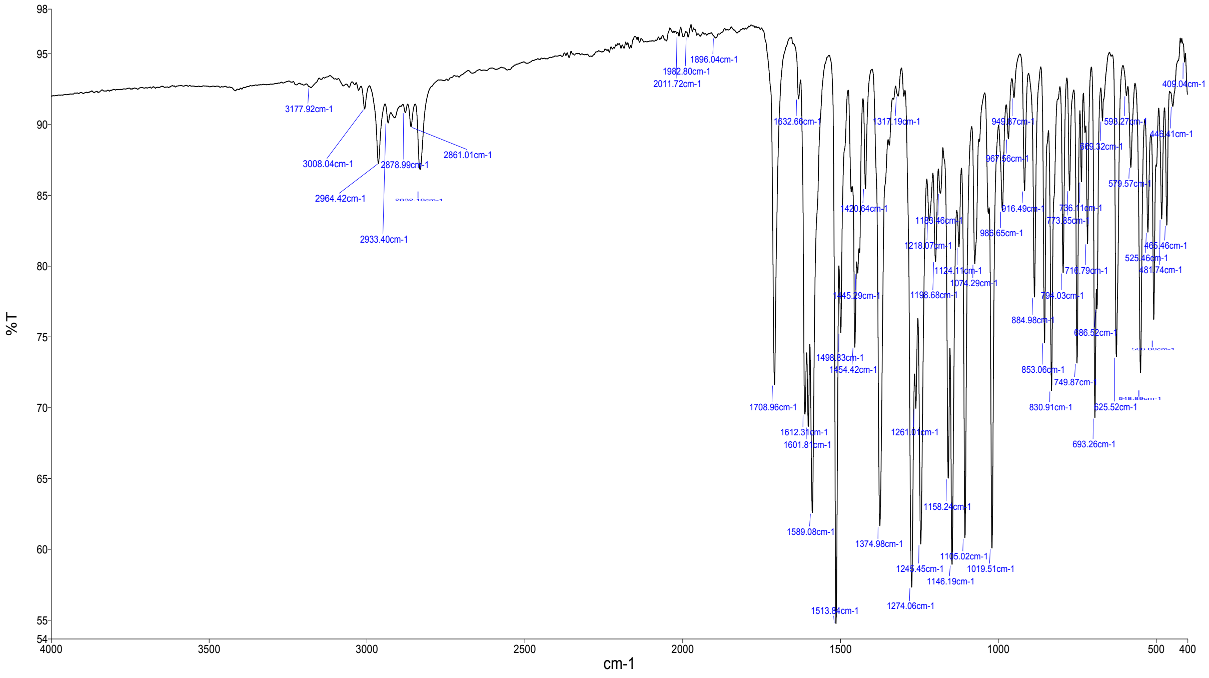

Figure S19. FT-IR spectrum of compound **7**

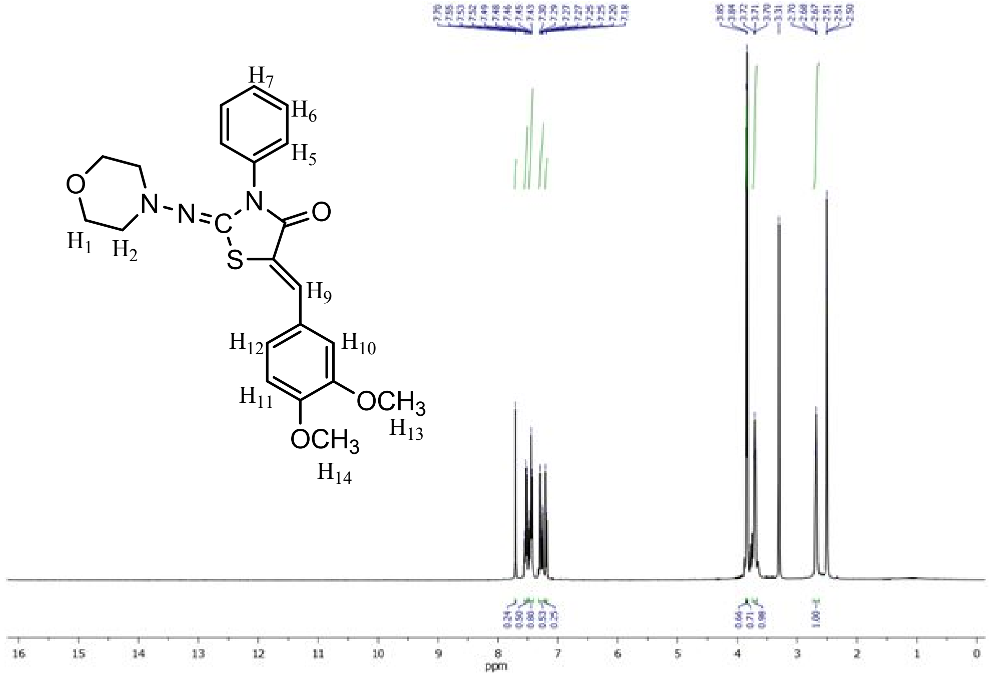

Figure S20. ^{1}H NMR spectrum of compound **7**

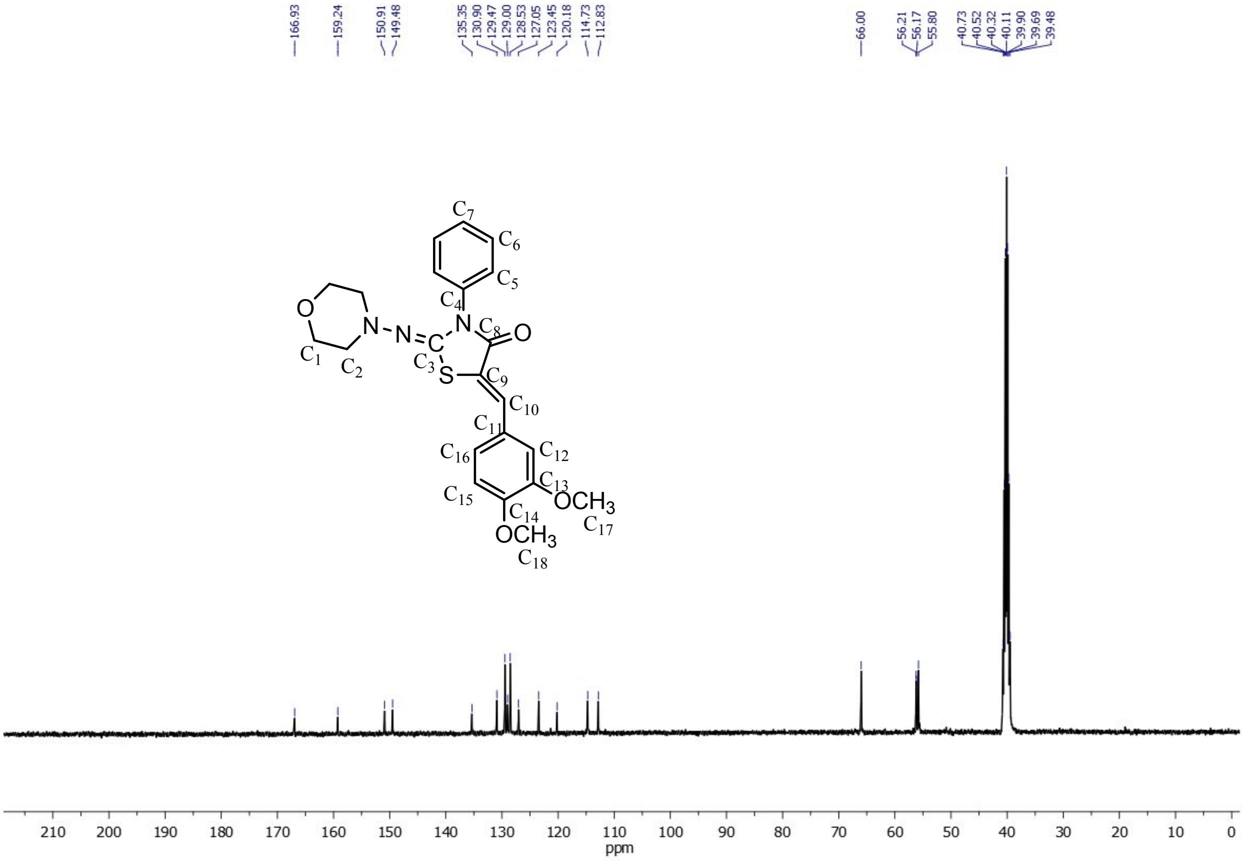

Figure S21. ^{13}C NMR spectrum of compound **7**

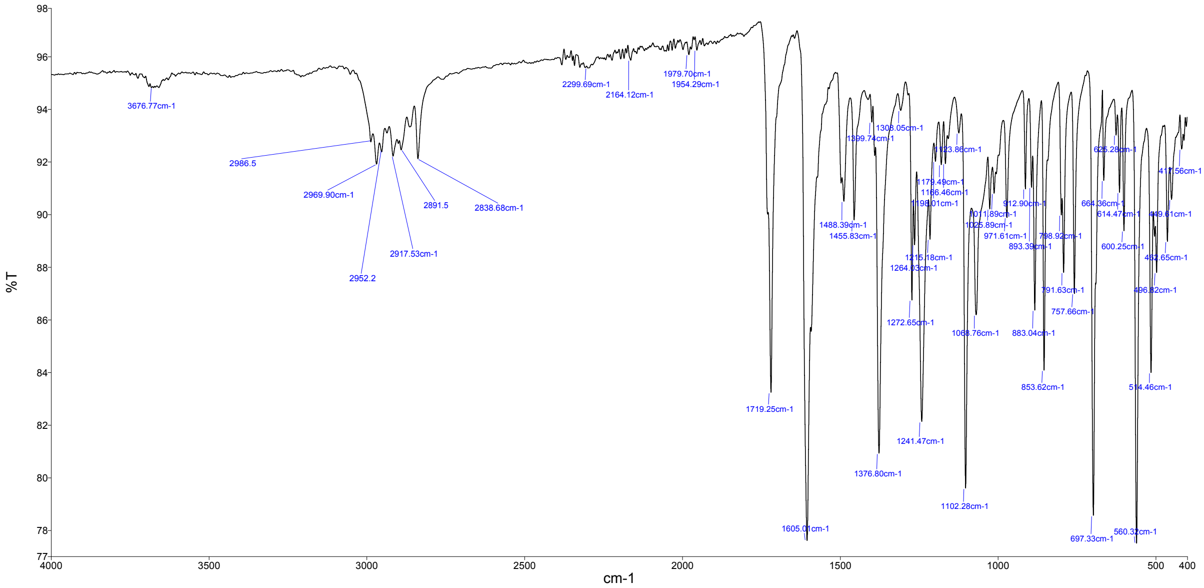

Figure S22. FT-IR spectrum of compound **8**

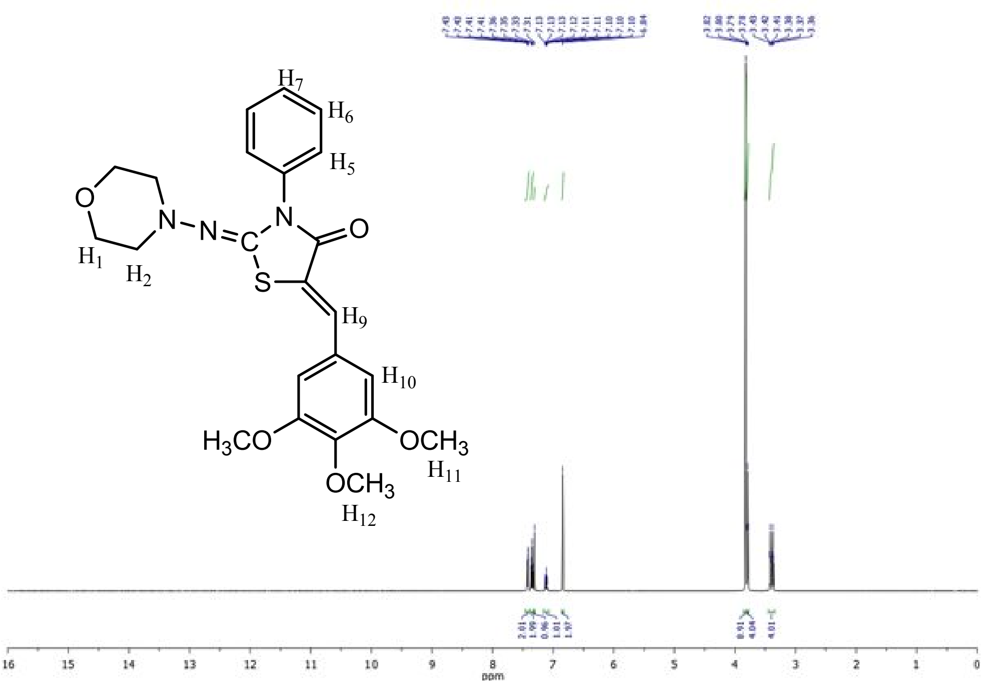

Figure S23. ^{1}H NMR spectrum of compound **8**

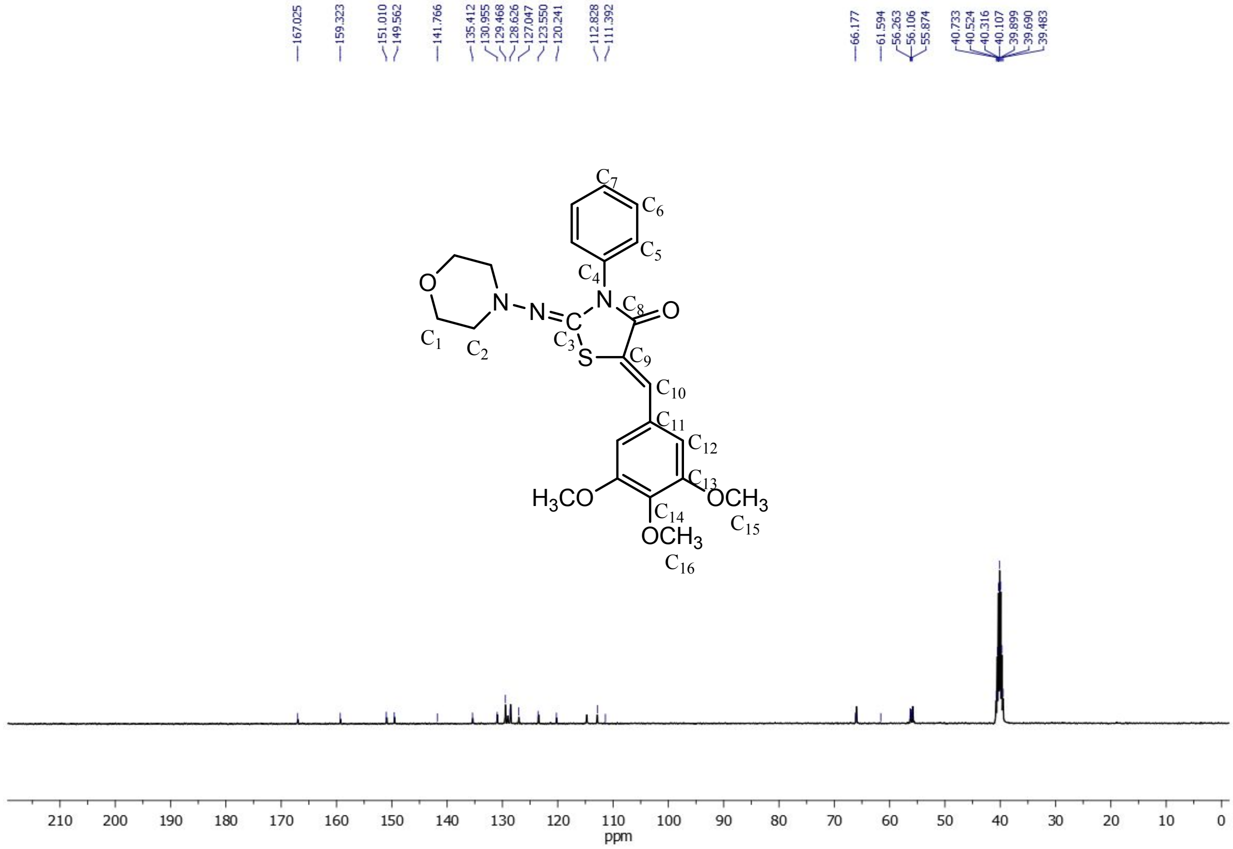

Figure S24. ^{13}C NMR spectrum of compound **8**

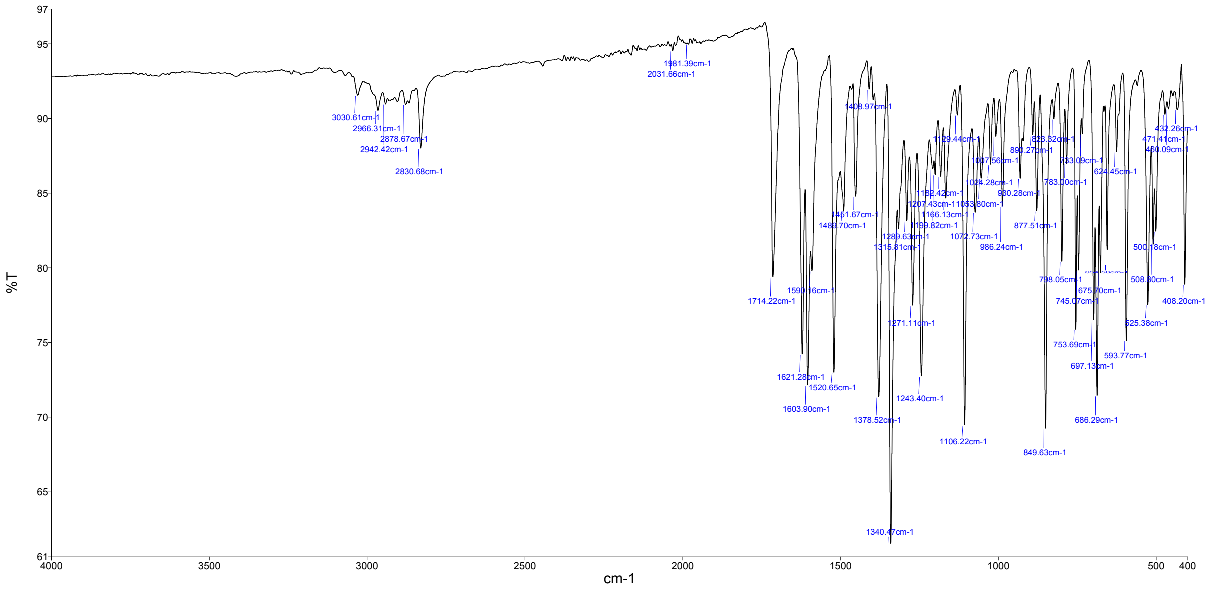

Figure S25. FT-IR spectrum of compound **9**

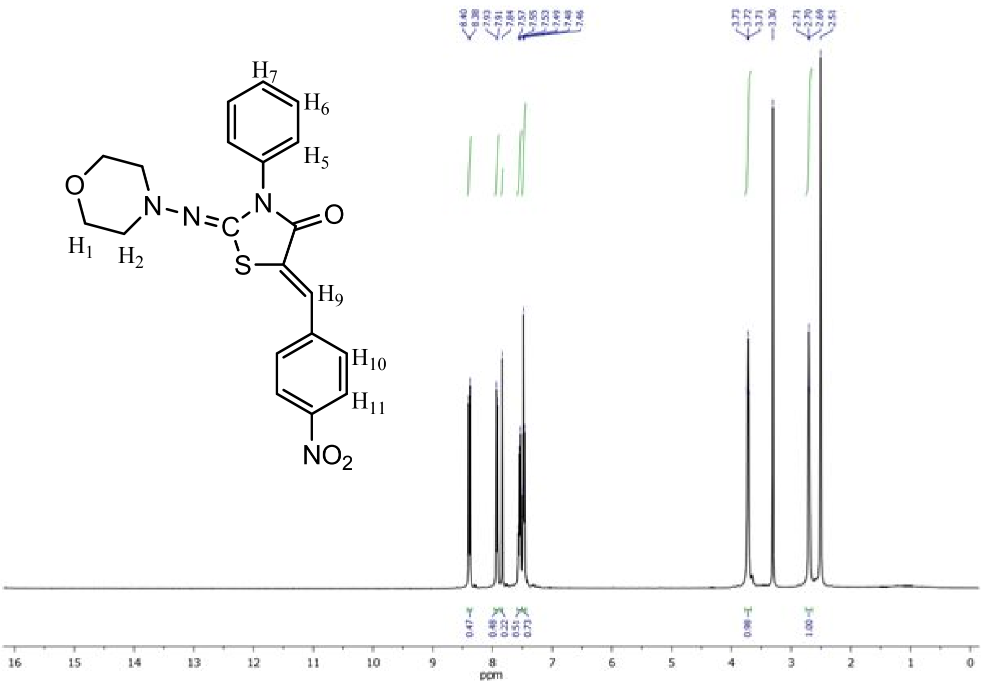

Figure S26. ^{1}H NMR spectrum of compound **9**

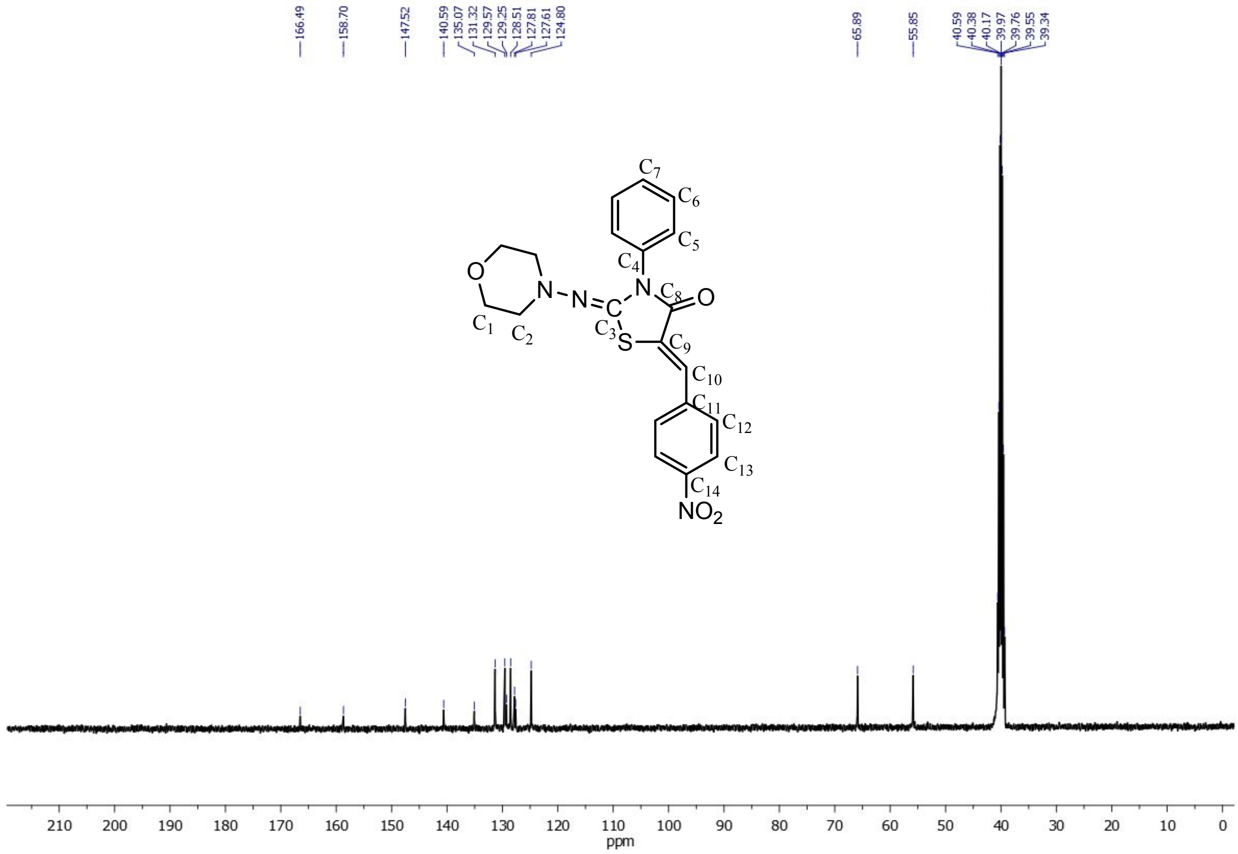

Figure S27. ^{13}C NMR spectrum of compound **9**

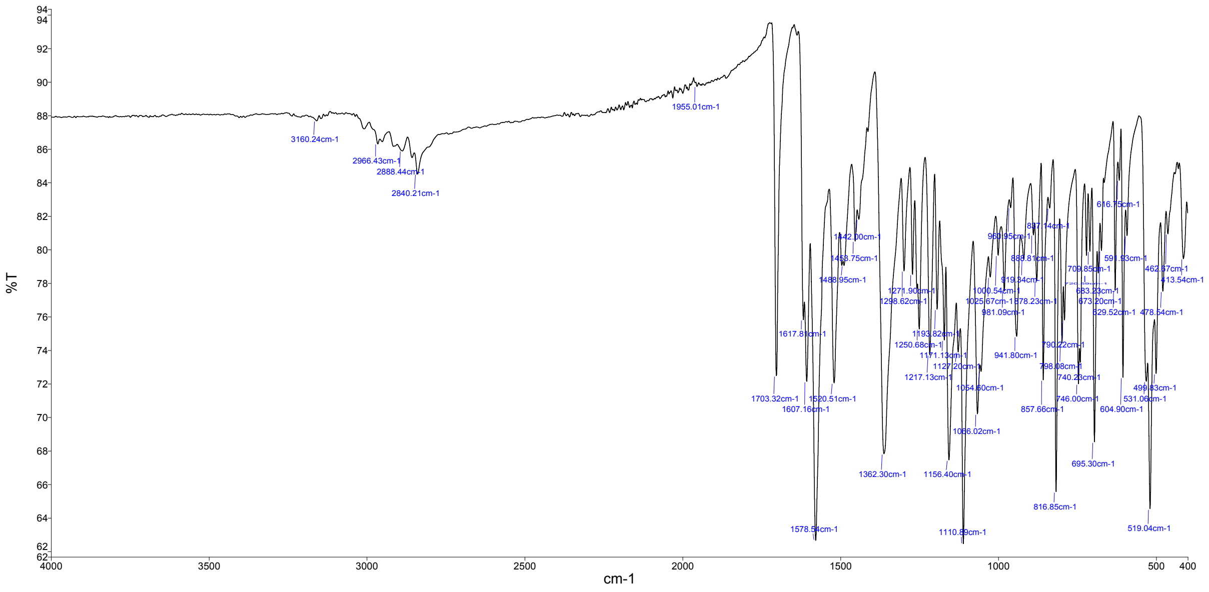

Figure S28. FT-IR spectrum of compound **10**

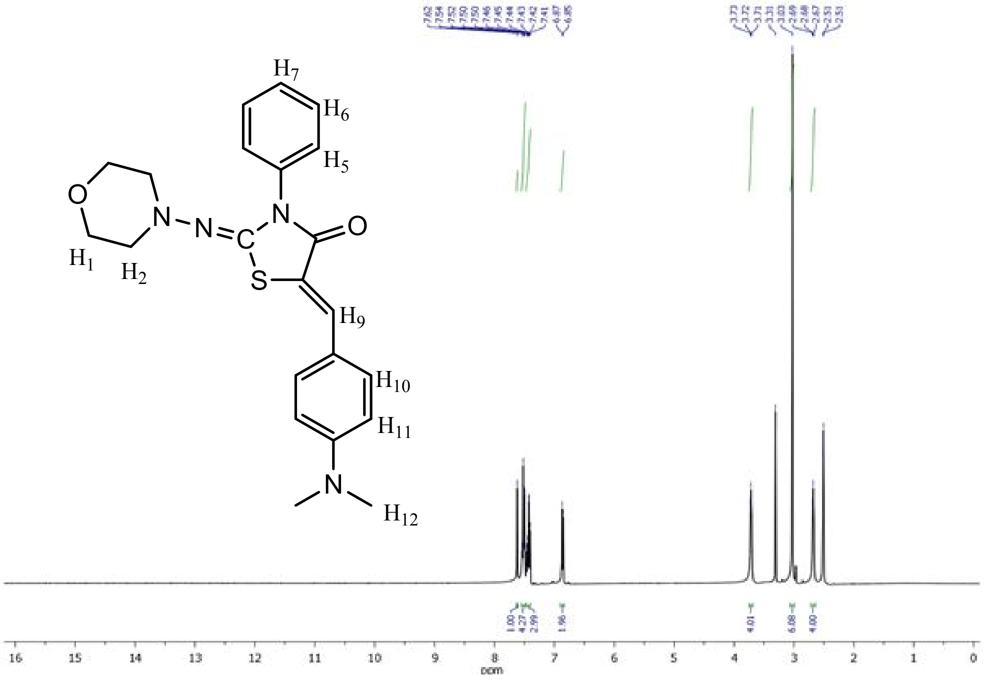

Figure S29. ^{1}H NMR spectrum of compound **10**

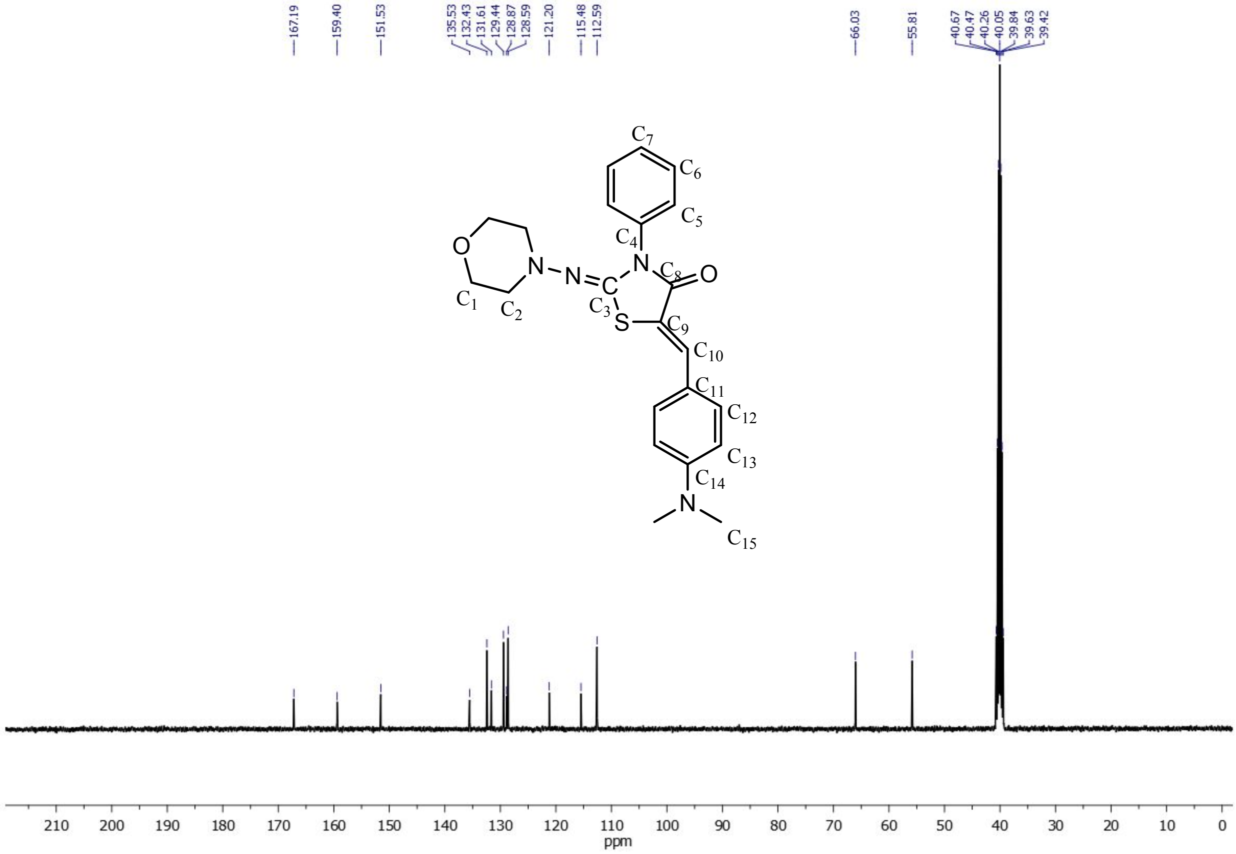

Figure S30. ^{13}C NMR spectrum of compound **10**

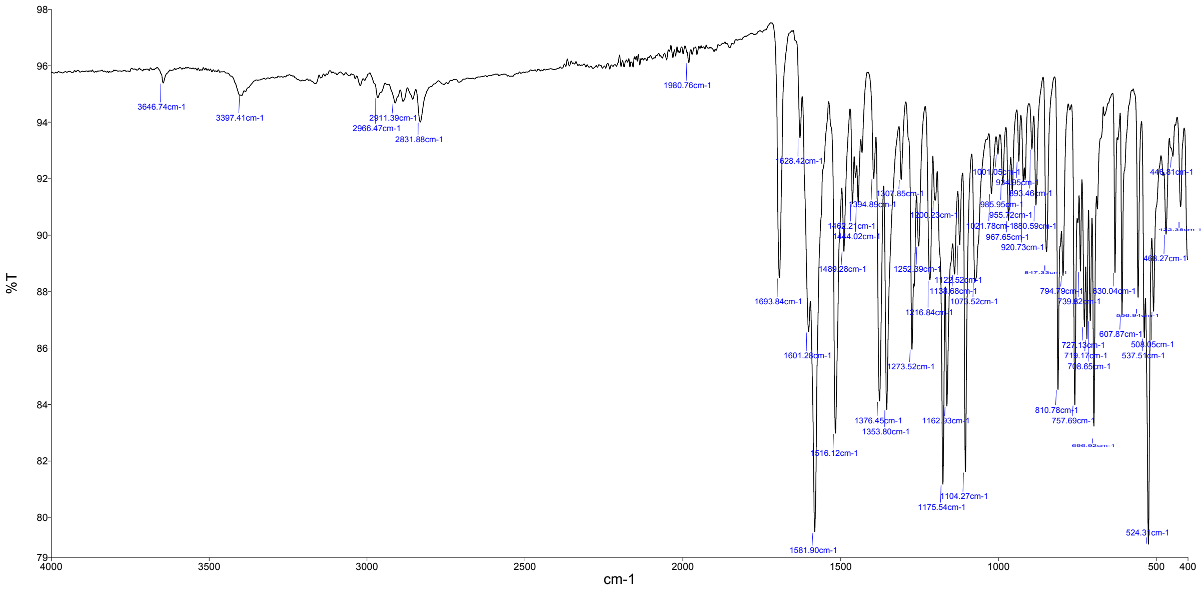

Figure S31. FT-IR spectrum of compound **11**

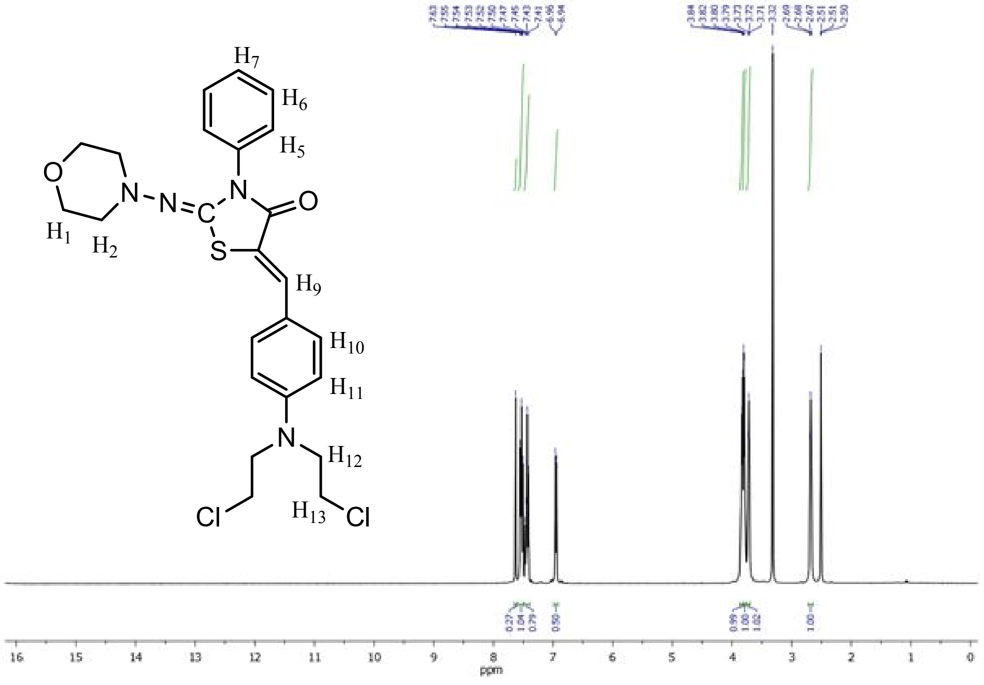

Figure S32. ^{1}H NMR spectrum of compound **11**

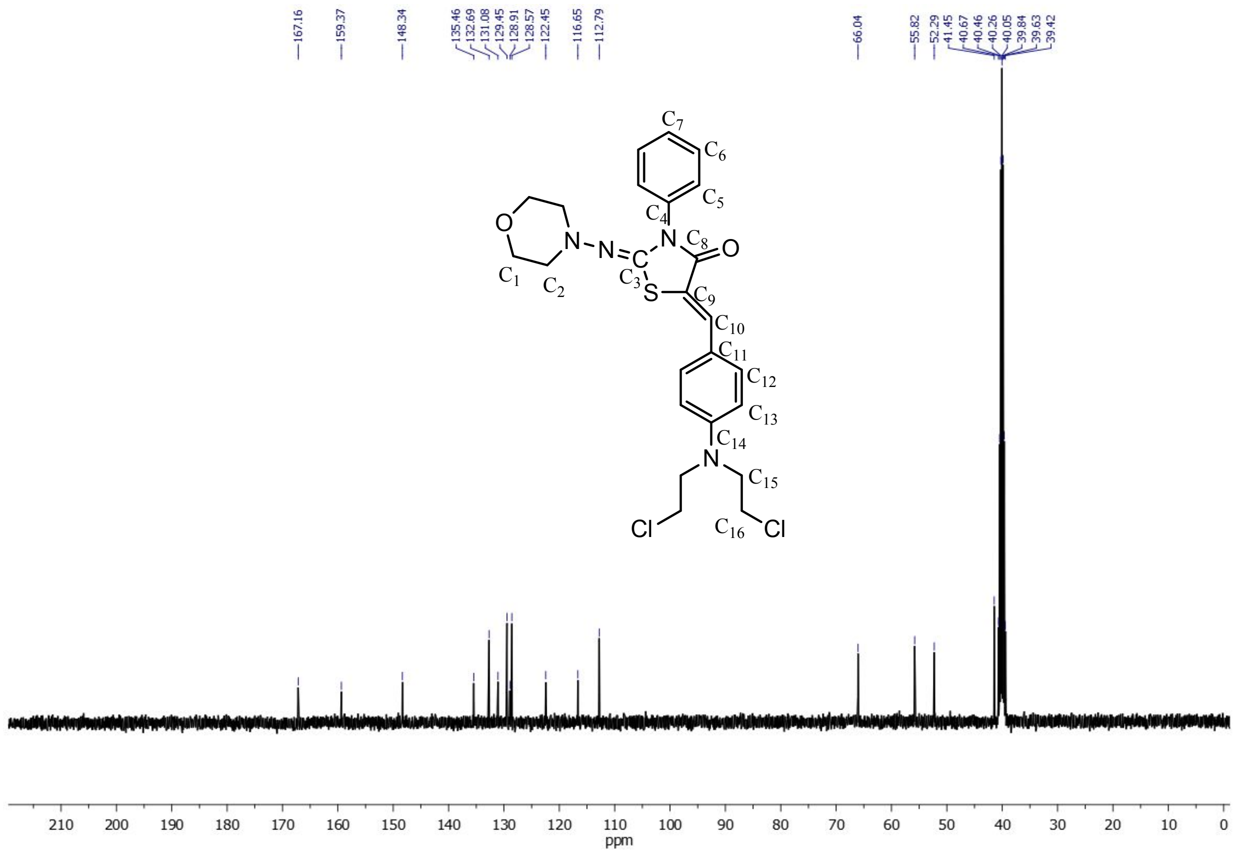

Figure S33. ^{13}C NMR spectrum of compound **11**

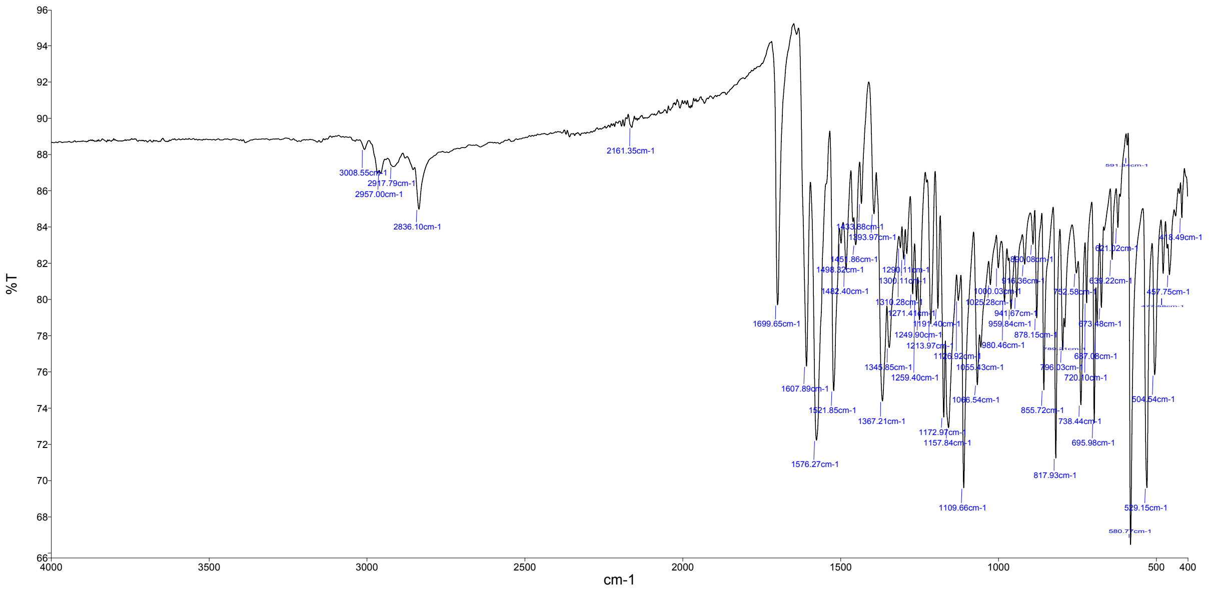

Figure S34. FT-IR spectrum of compound **12**

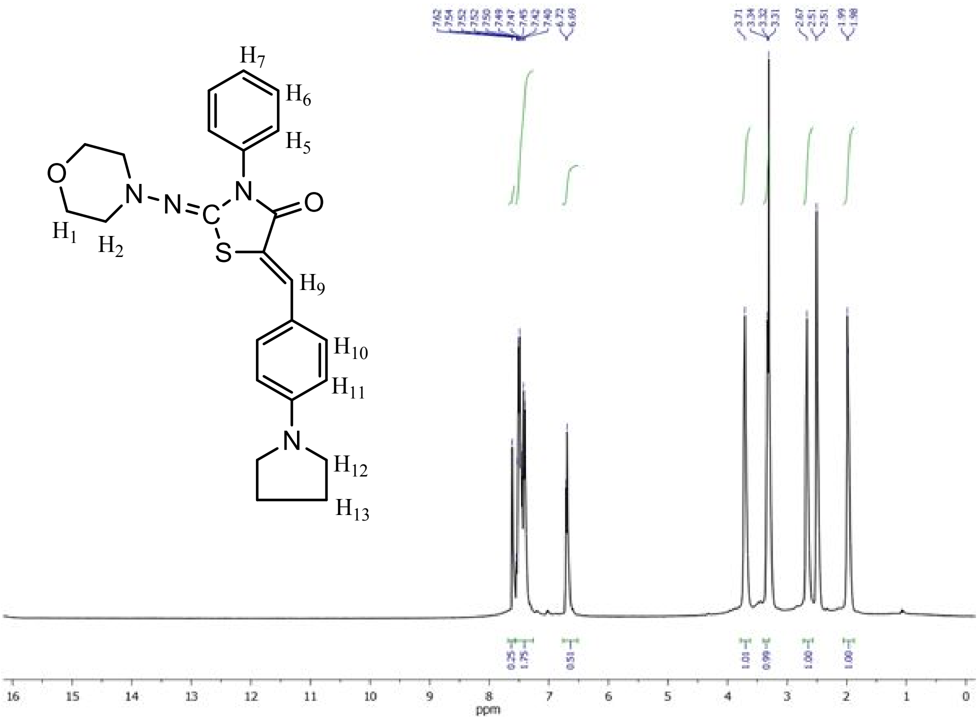

Figure S35. ^{1}H NMR spectrum of compound **12**

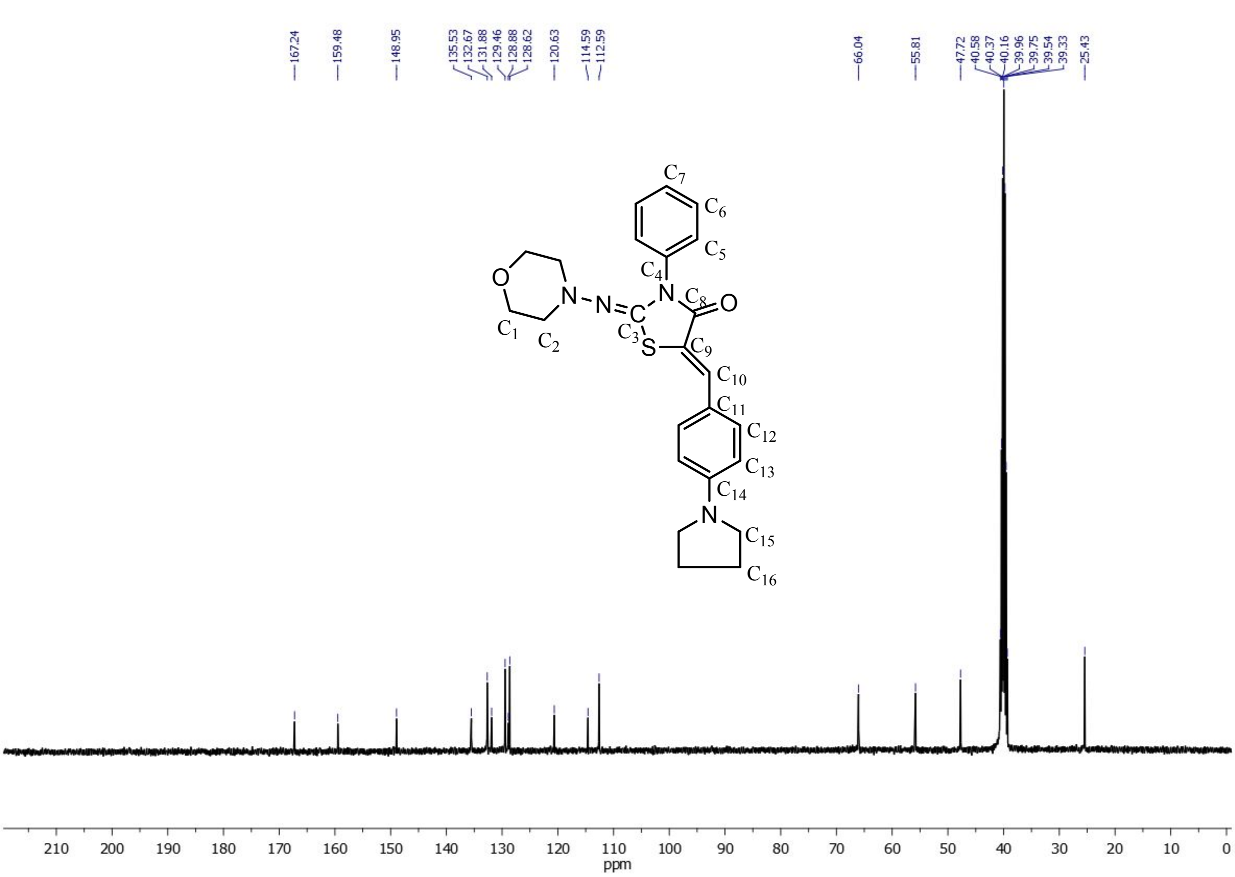

Figure S36. ^{13}C NMR spectrum of compound **12**

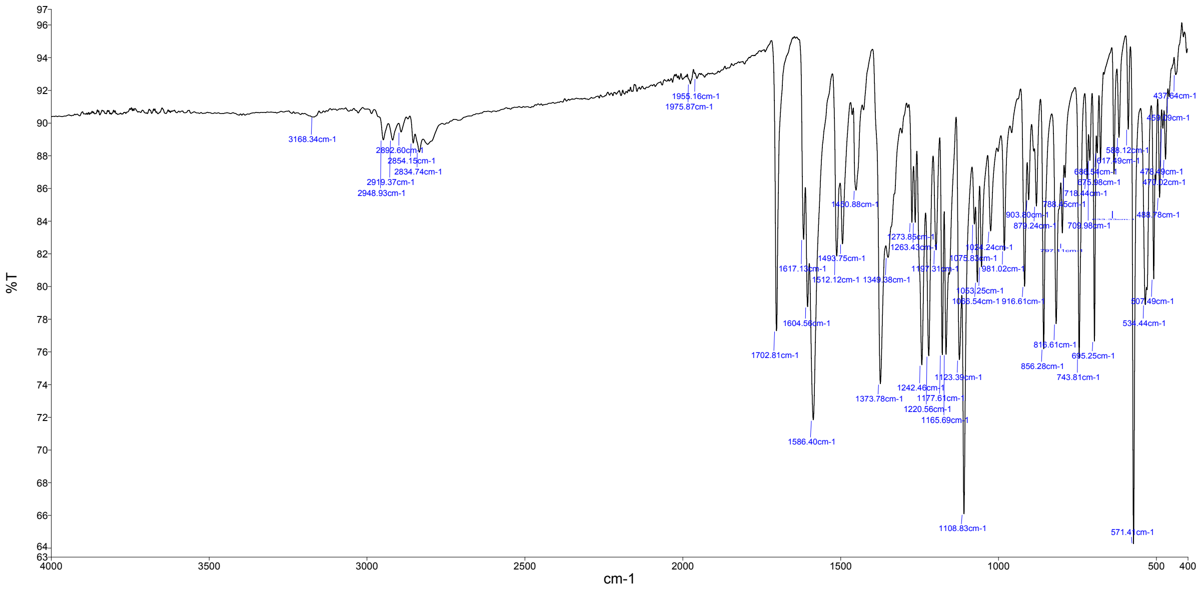

Figure S37. FT-IR spectrum of compound **13**

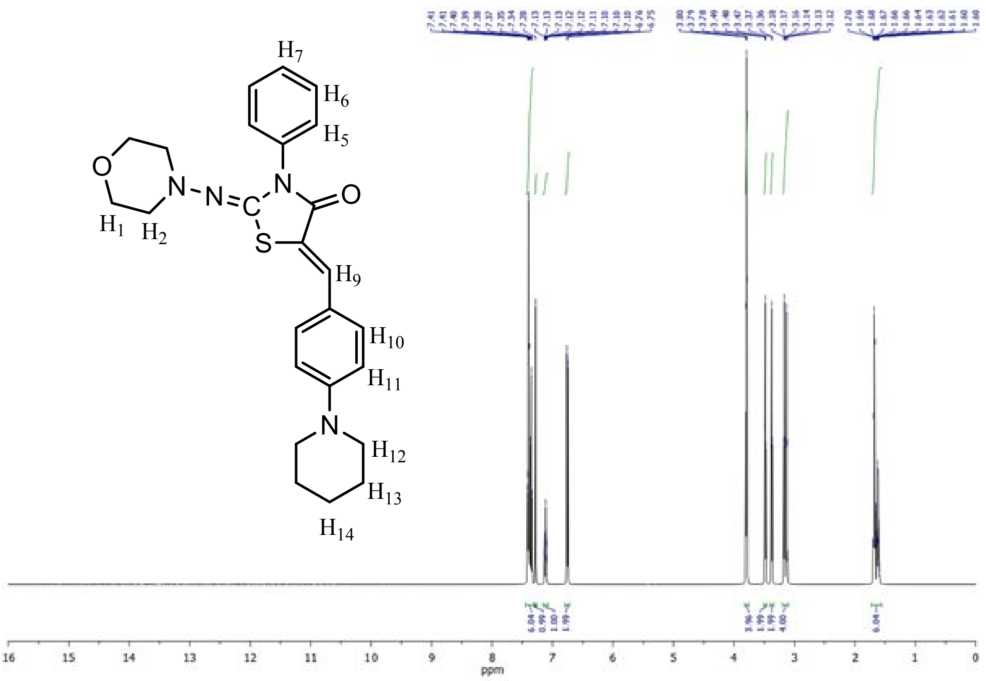

Figure S38. ^{1}H NMR spectrum of compound 1**3**

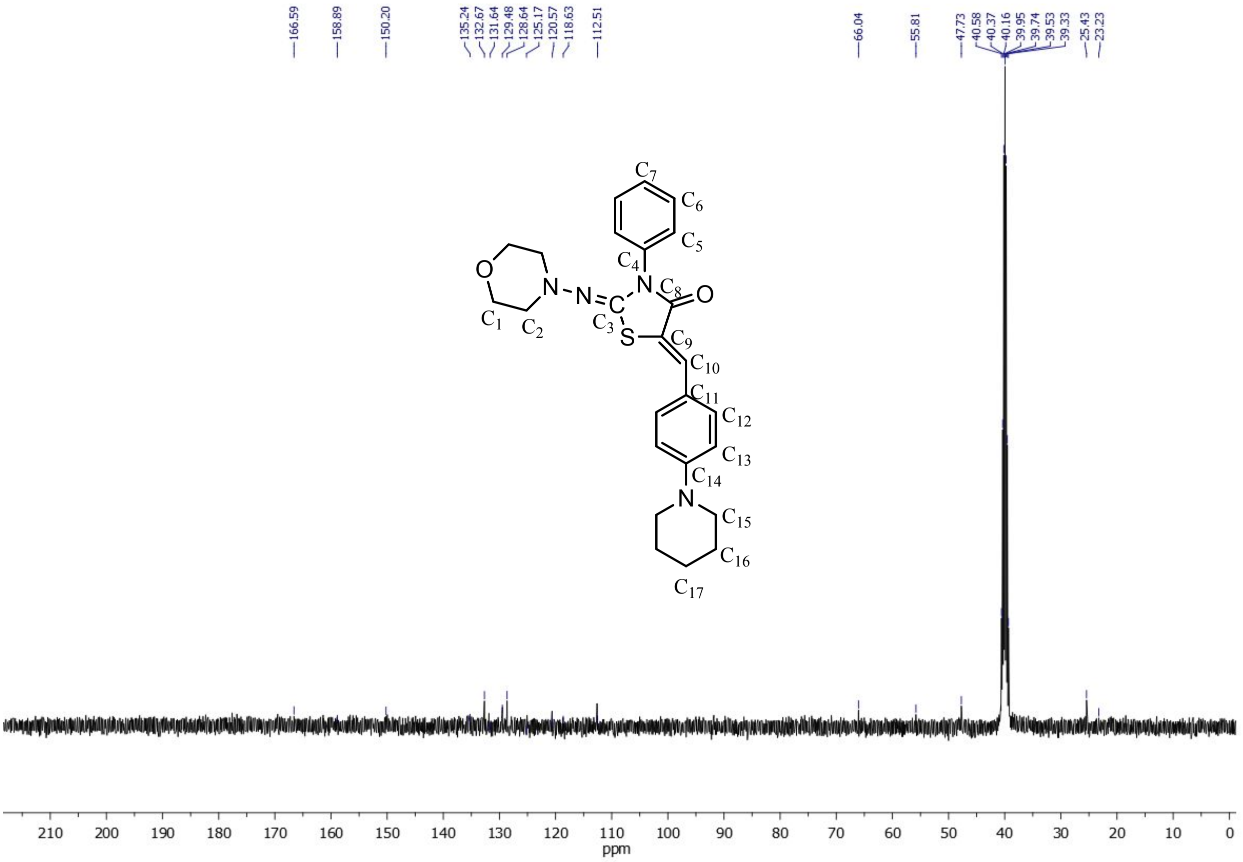

Figure S39. ^{13}C NMR spectrum of compound 1**3**

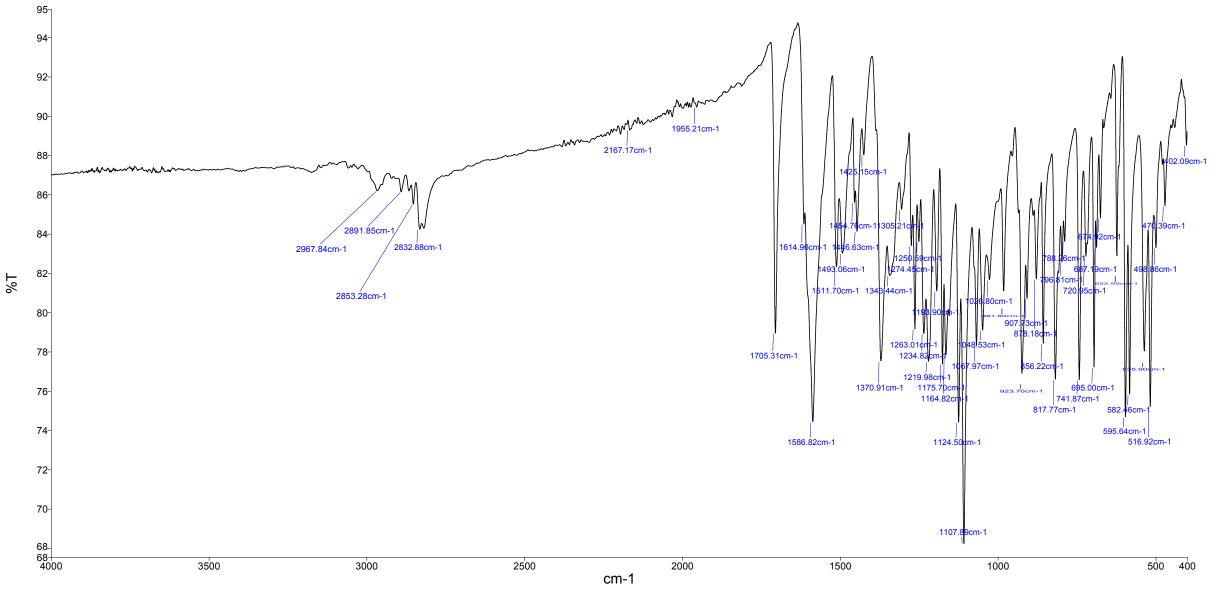

Figure S40. FT-IR spectrum of compound **14**

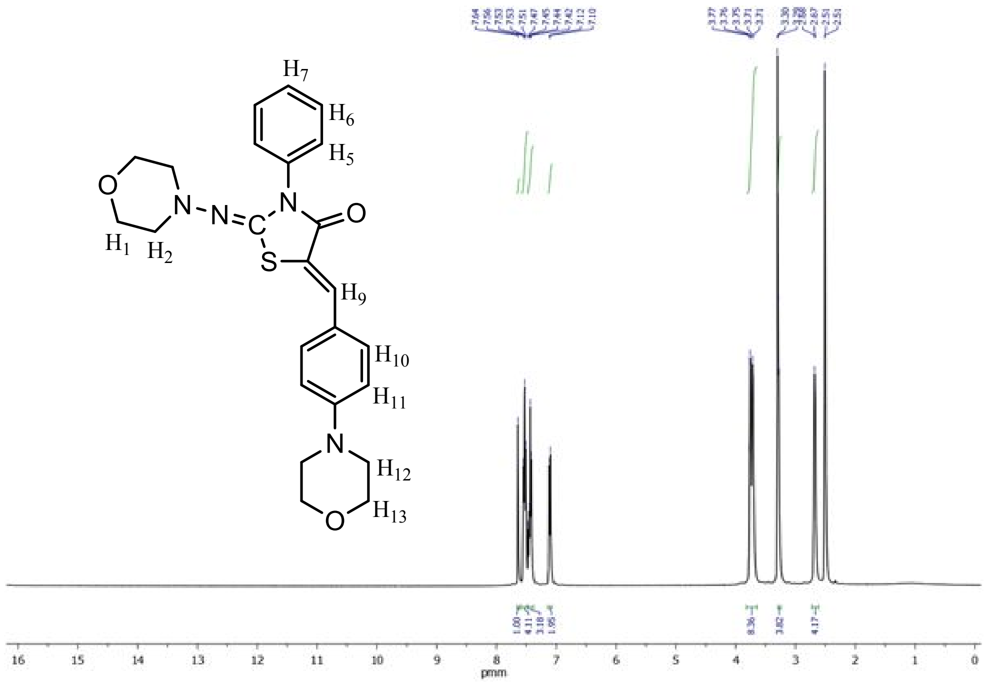

Figure S41. ^{1}H NMR spectrum of compound **14**

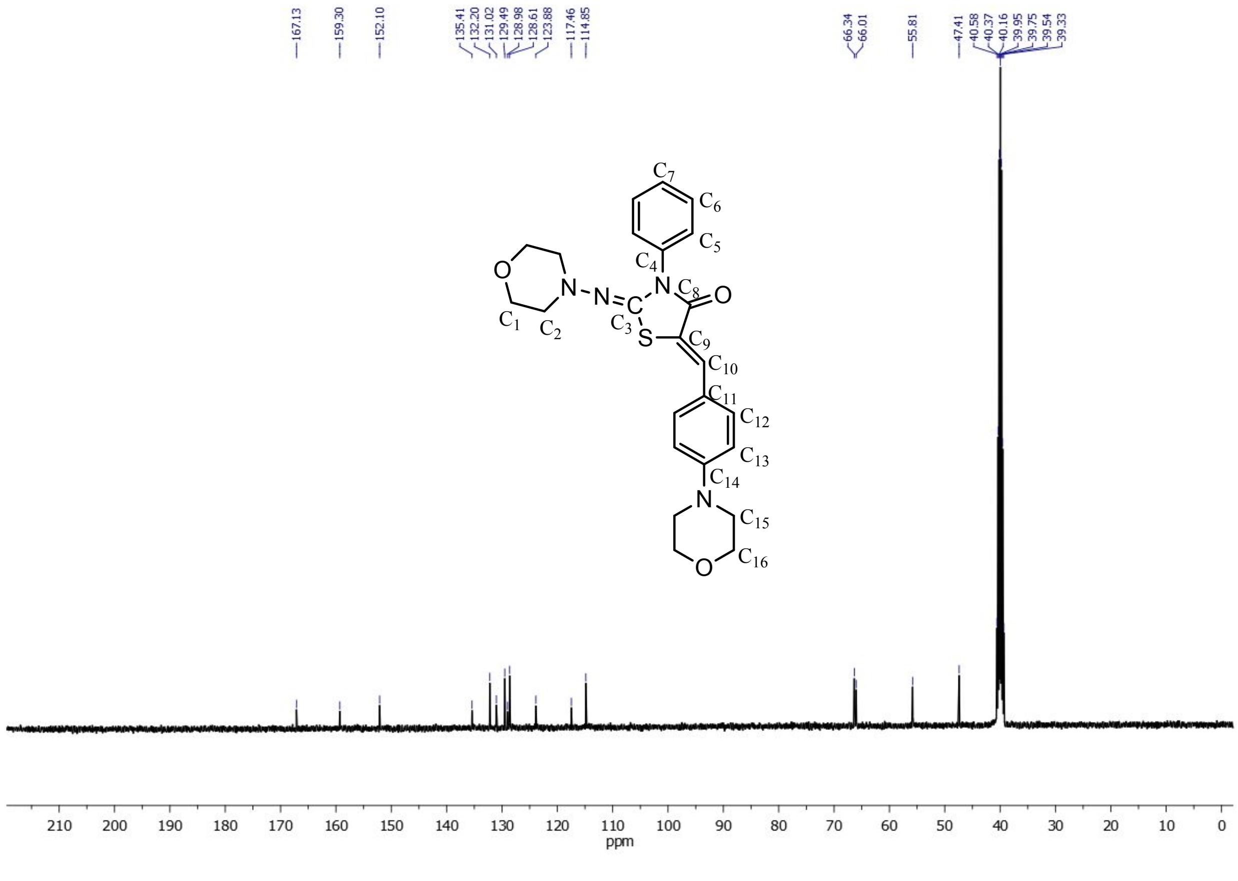

Figure S42. ^{13}C NMR spectrum of compound **14**

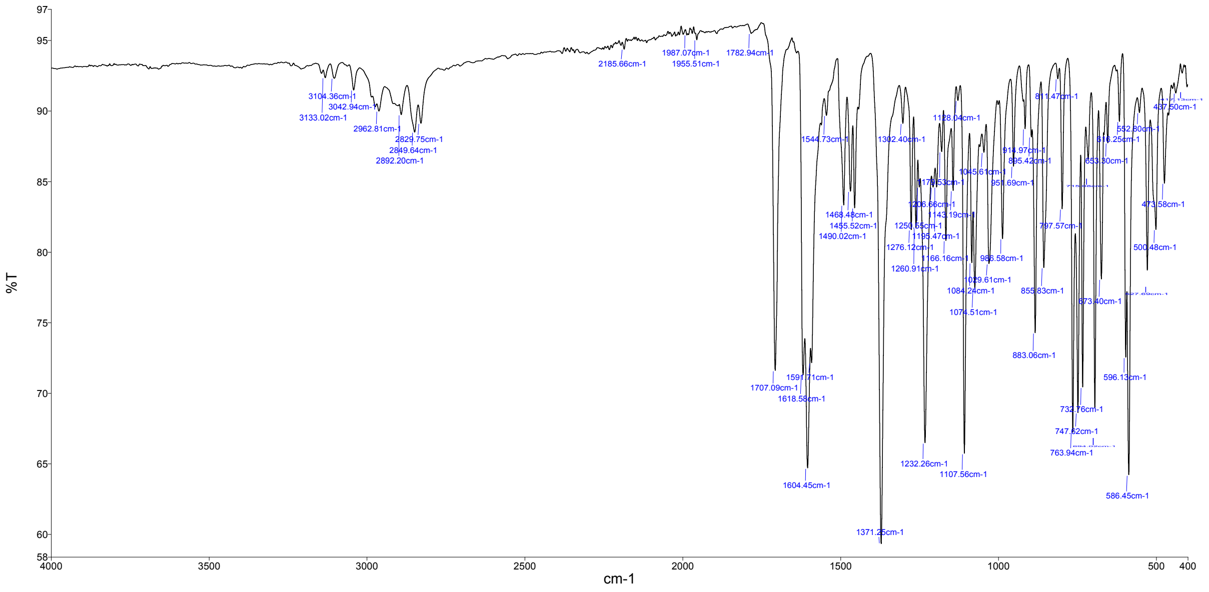

Figure S43. FT-IR spectrum of compound **15**

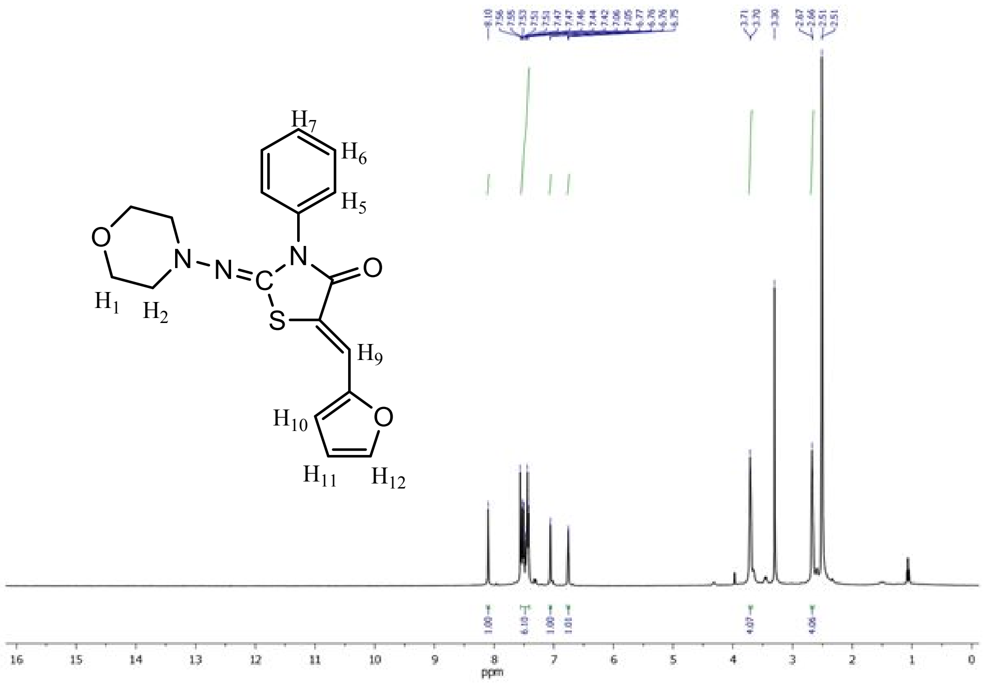

Figure S44. ^{1}H NMR spectrum of compound **15**

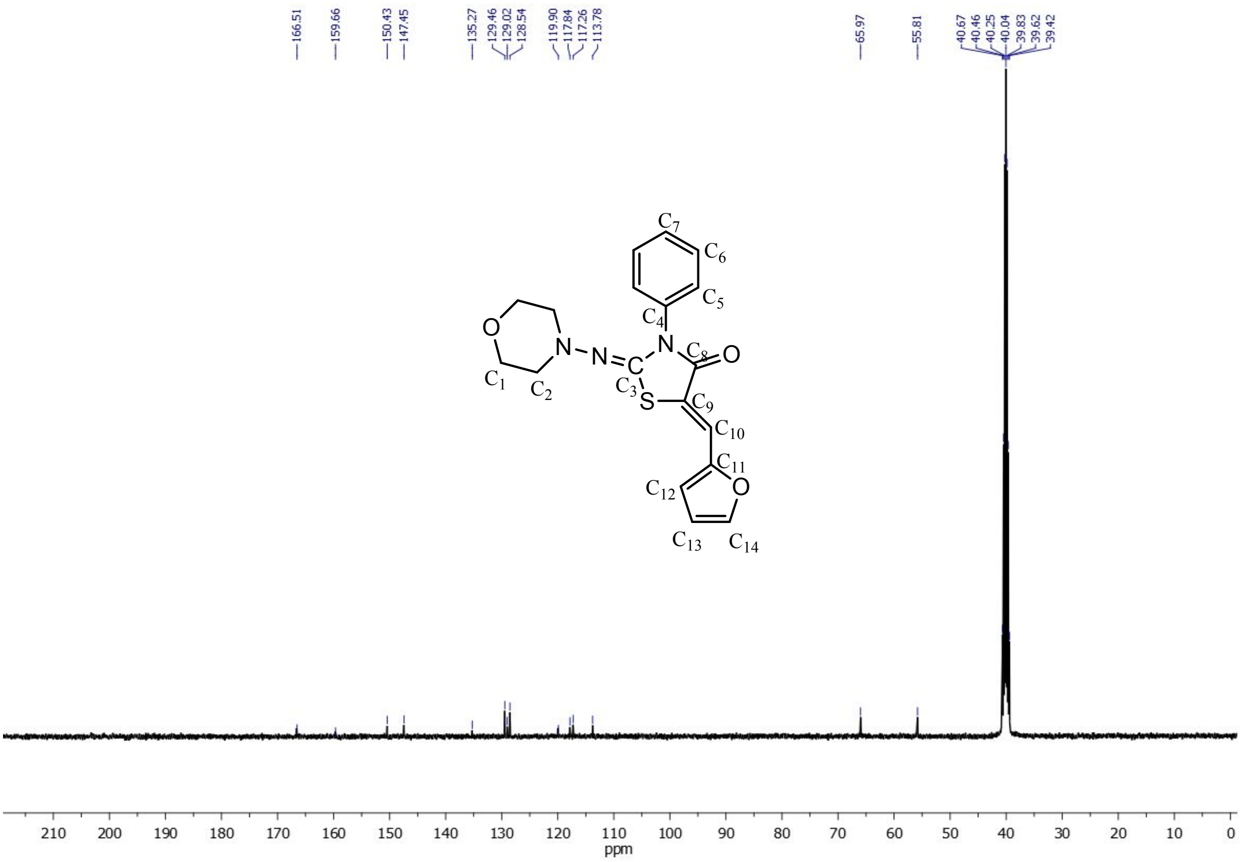

Figure S45. ^{13}C NMR spectrum of compound **15**

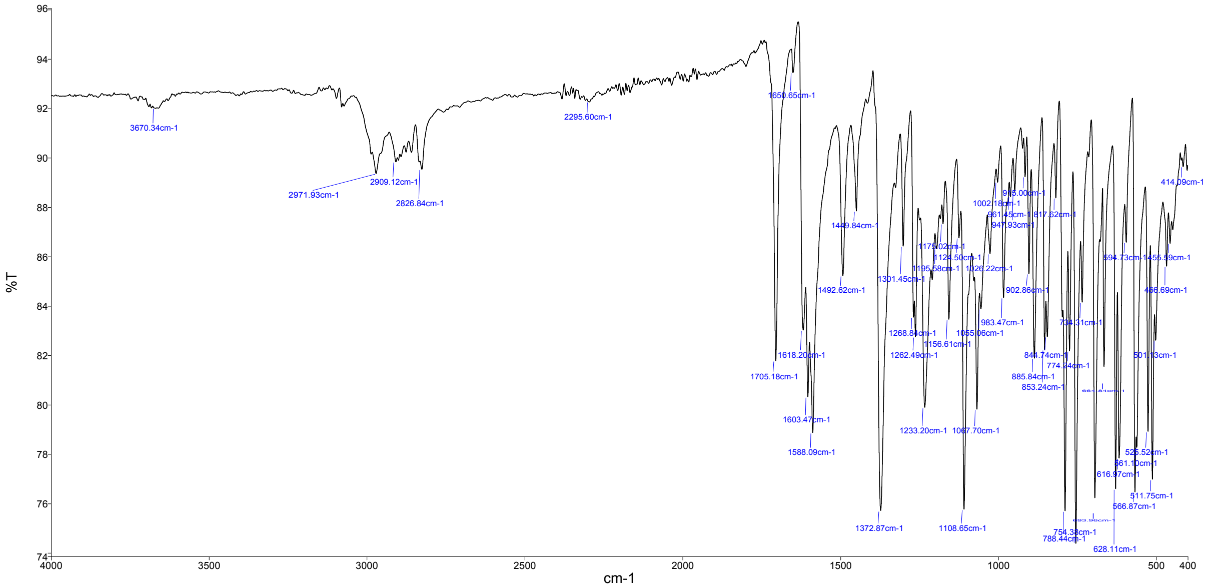

Figure S46. FT-IR spectrum of compound **16**

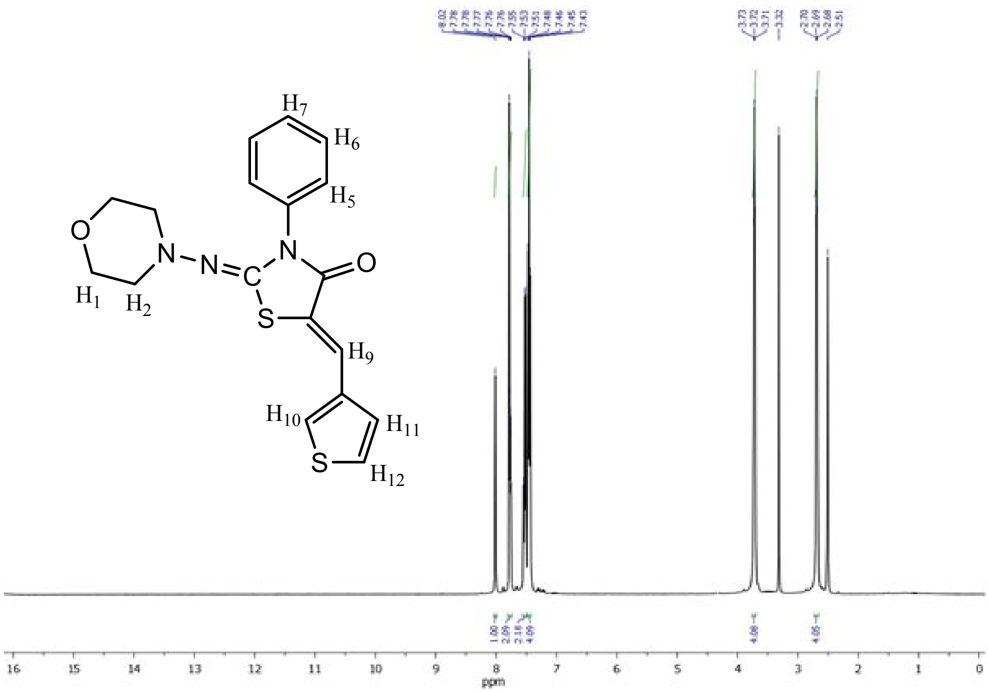

Figure S47. ^{1}H NMR spectrum of compound **16**

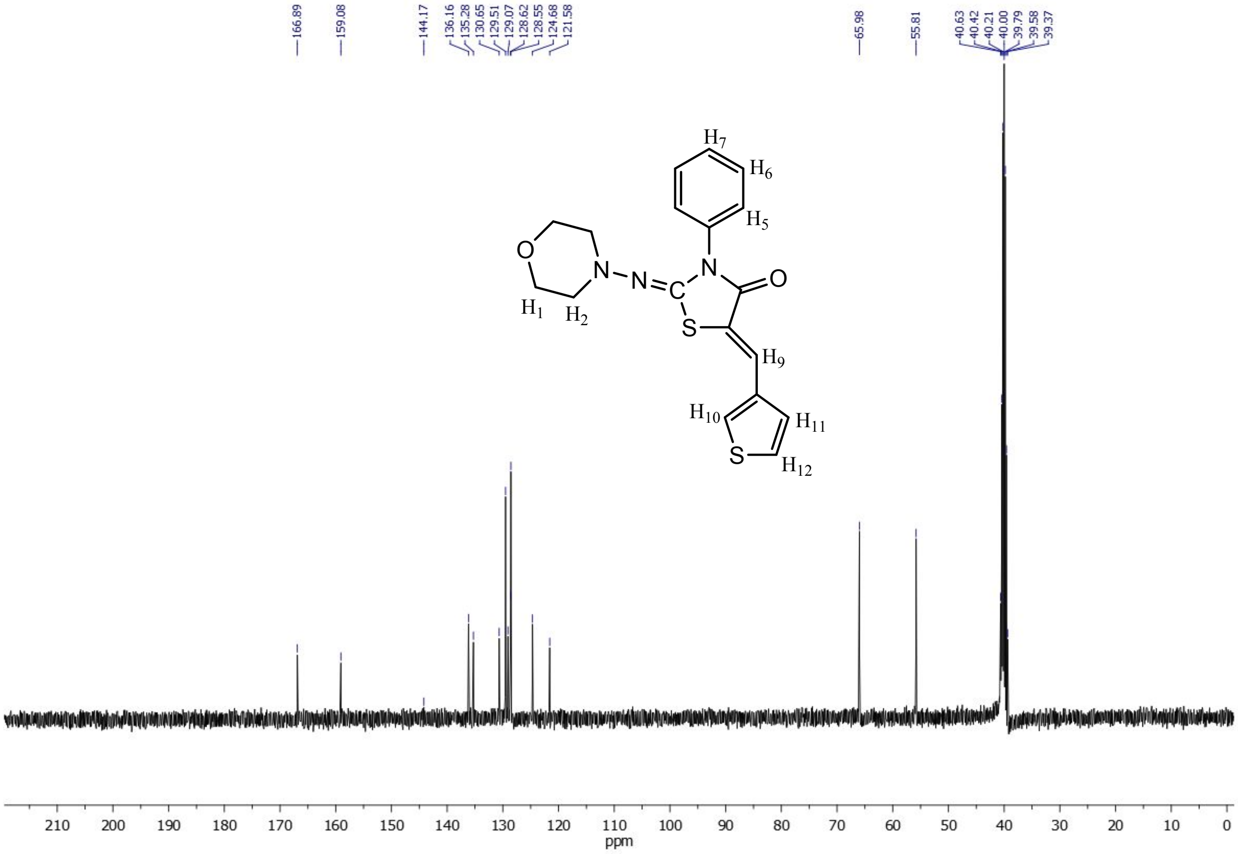

Figure S48. ^{13}C NMR spectrum of compound **16**

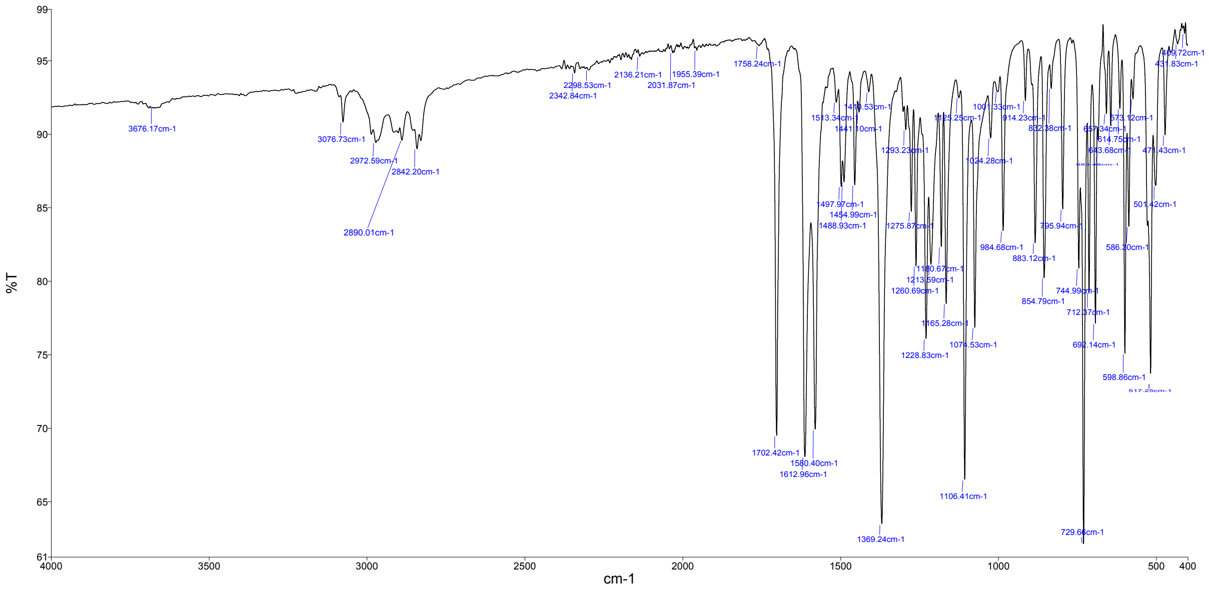

Figure S49. FT-IR spectrum of compound **17**

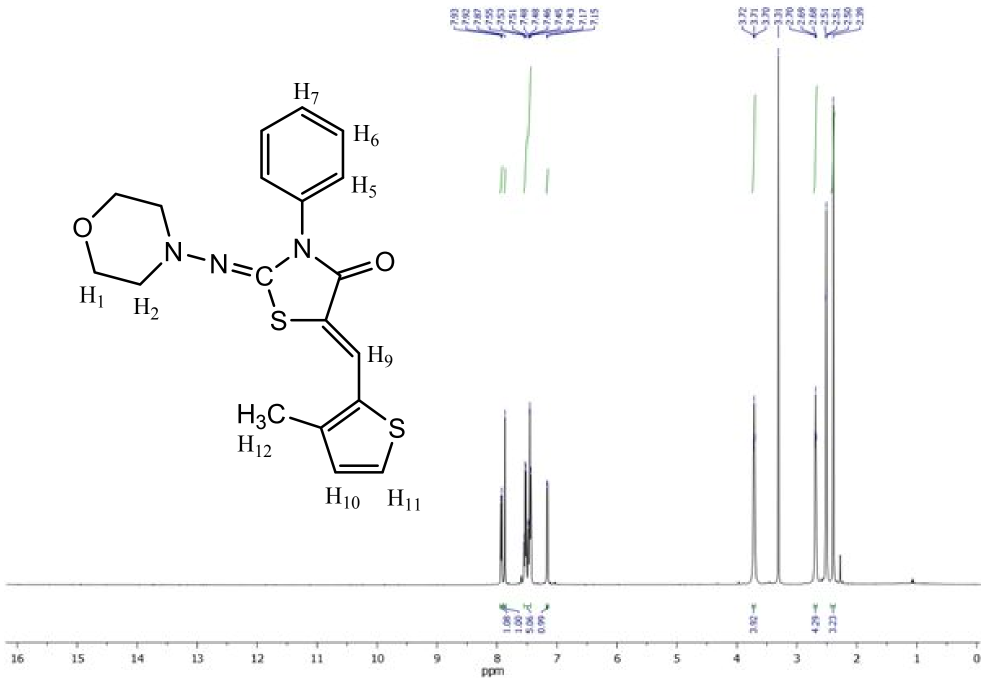

Figure S50. ^{1}H NMR spectrum of compound **17**

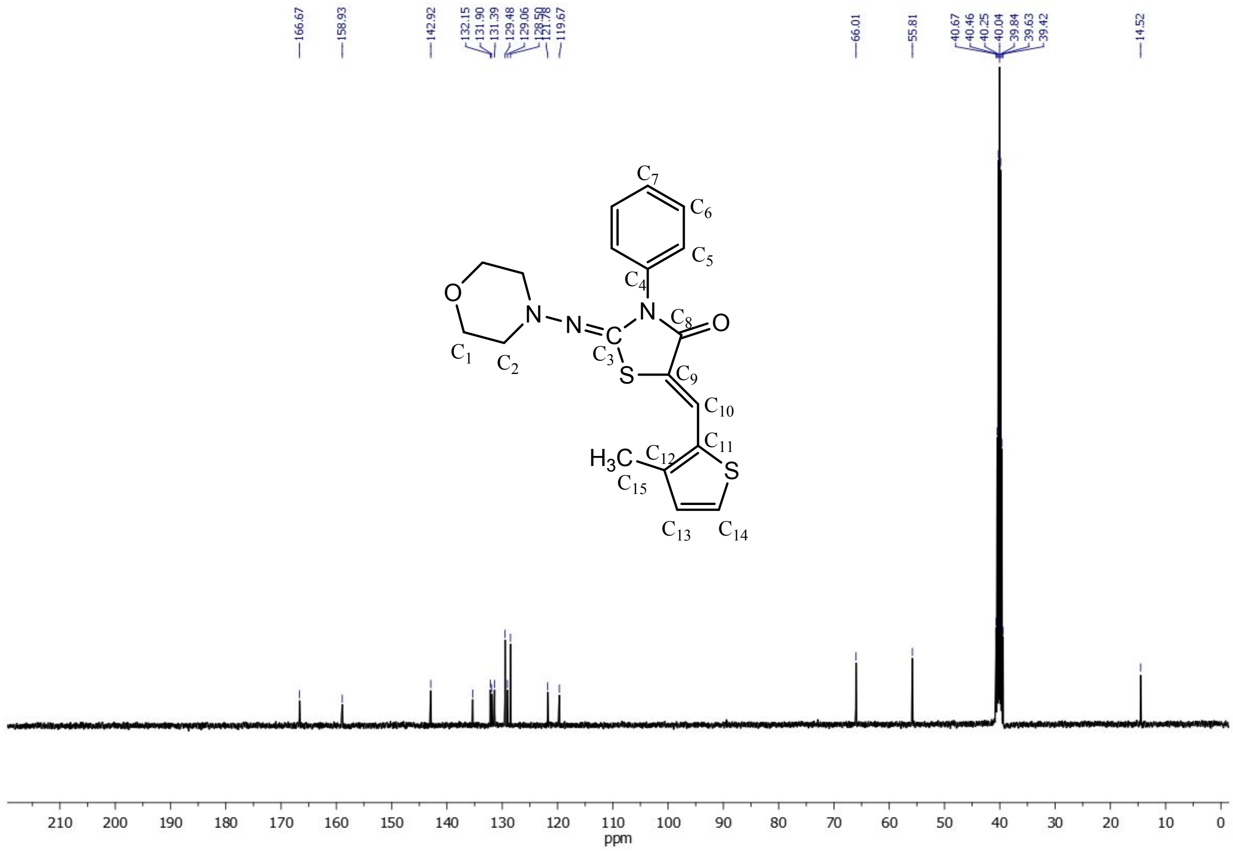

Figure S51. ^{13}C NMR spectrum of compound **17**

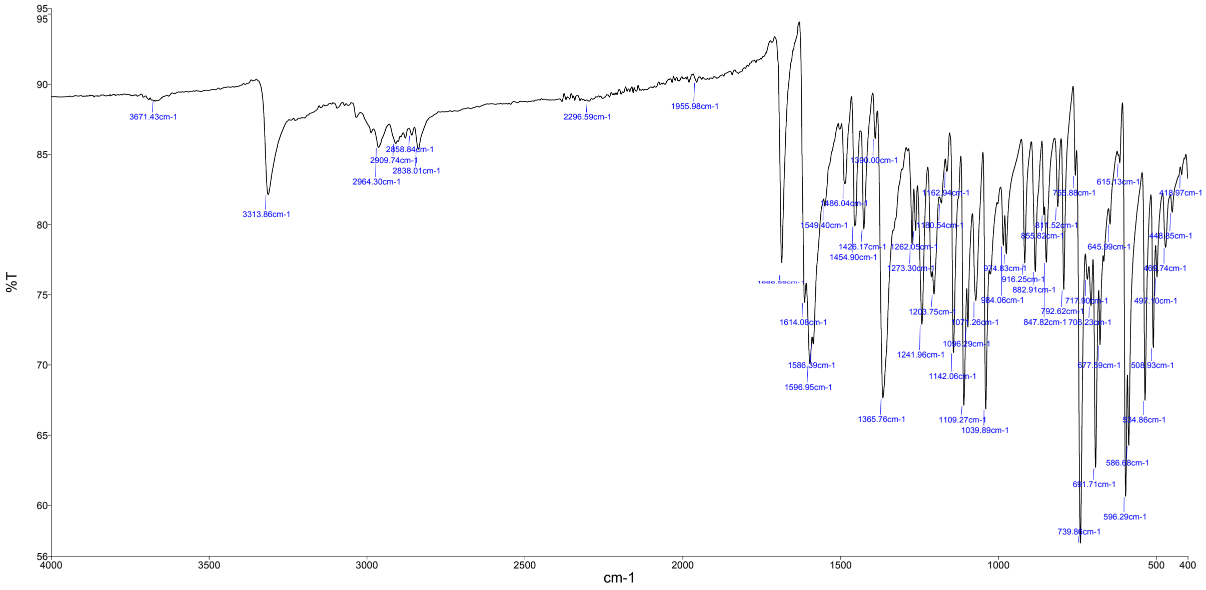

Figure S52. FT-IR spectrum of compound **18**

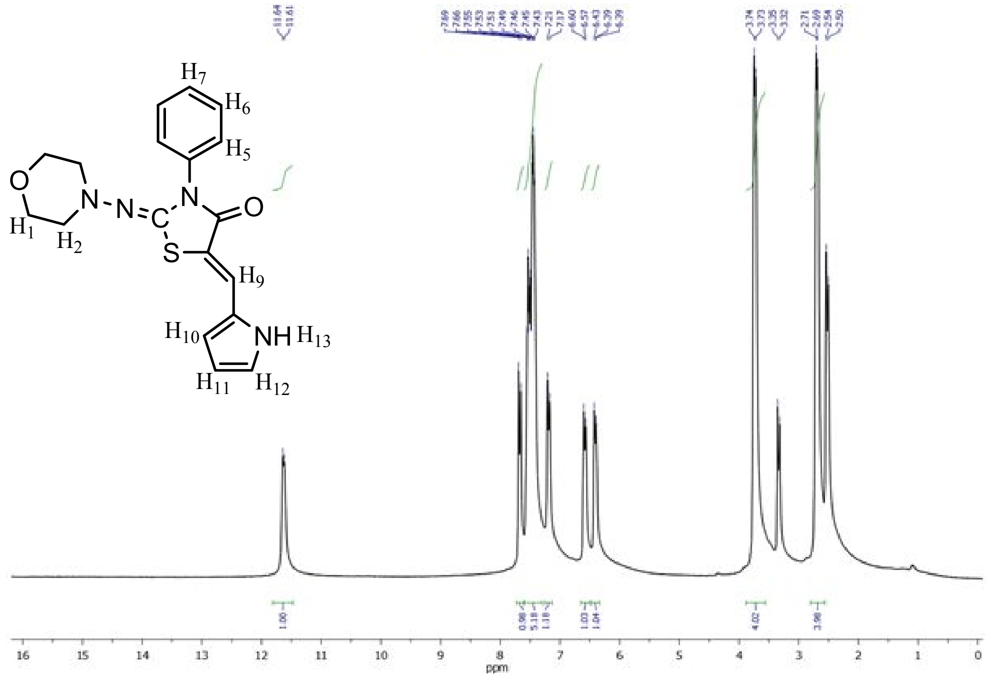

Figure S53. ^{1}H NMR spectrum of compound **18**

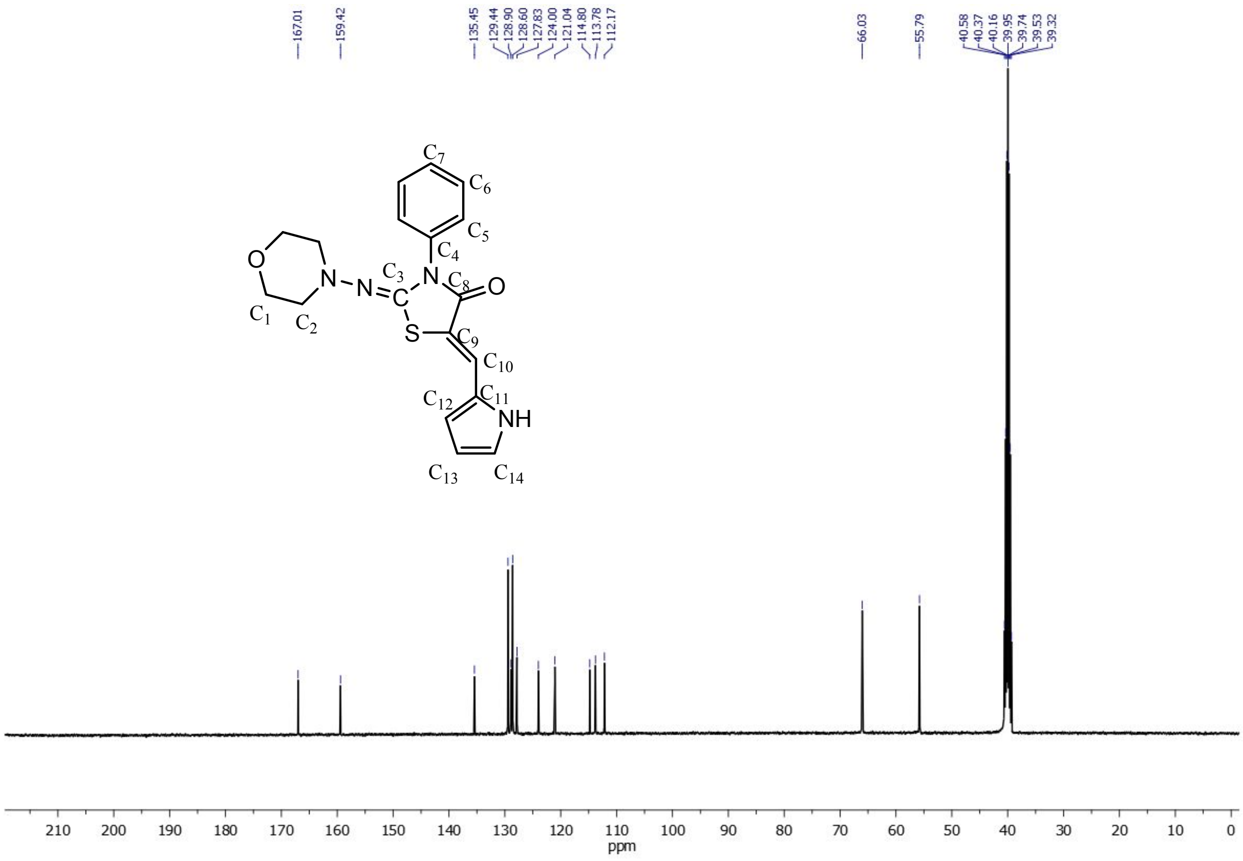

Figure S54. ^{13}C NMR spectrum of compound **18**

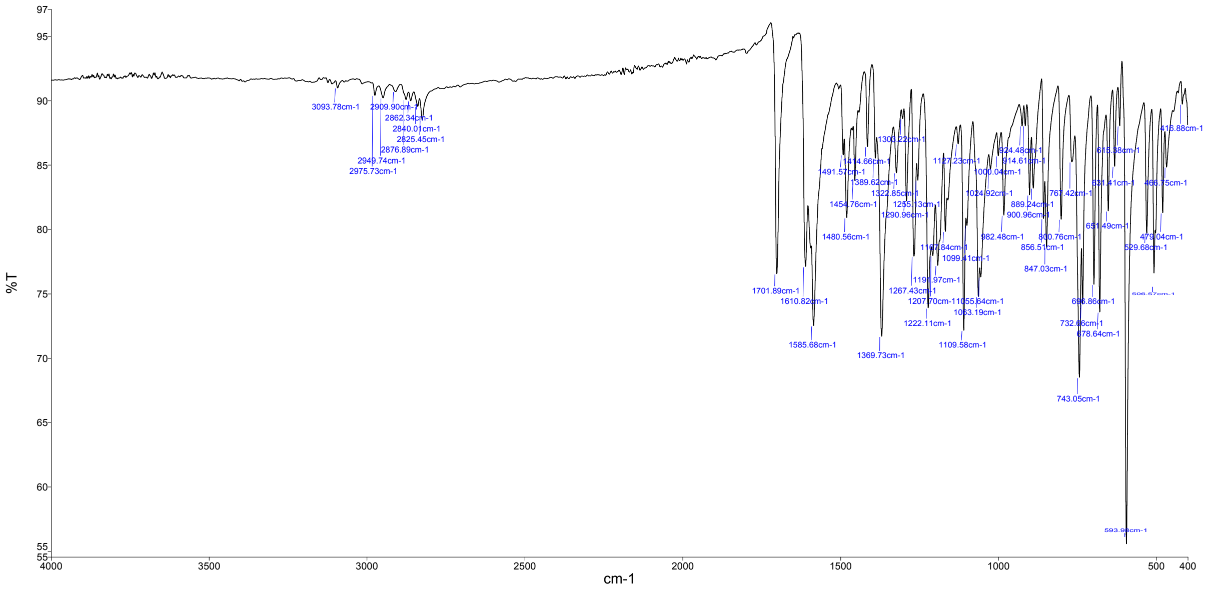

Figure S55. FT-IR spectrum of compound **19**

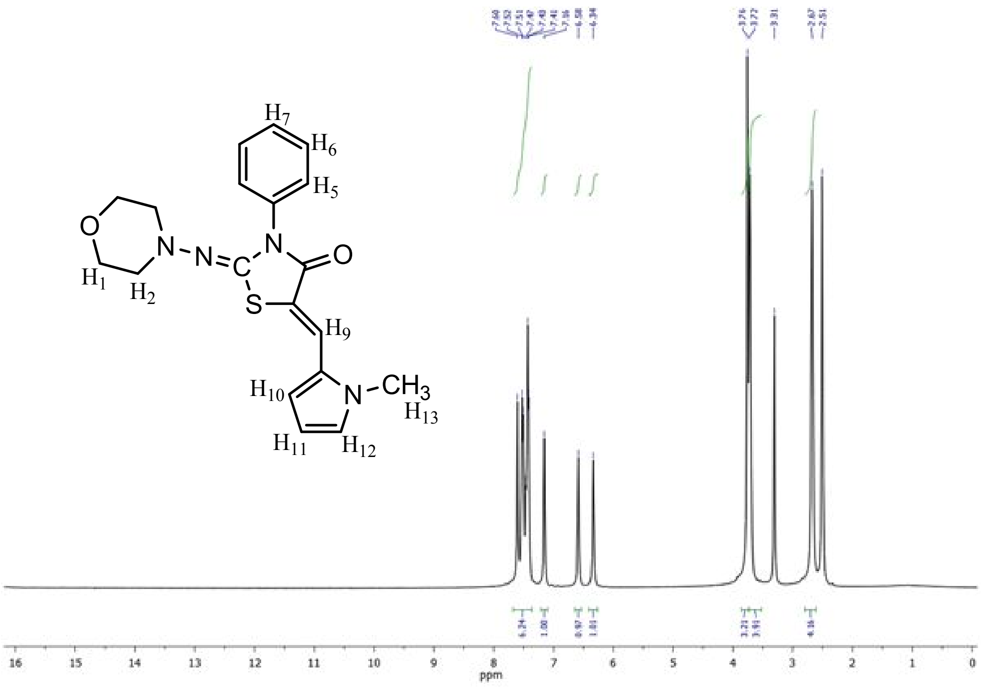

Figure S56. ^{1}H NMR spectrum of compound **19**

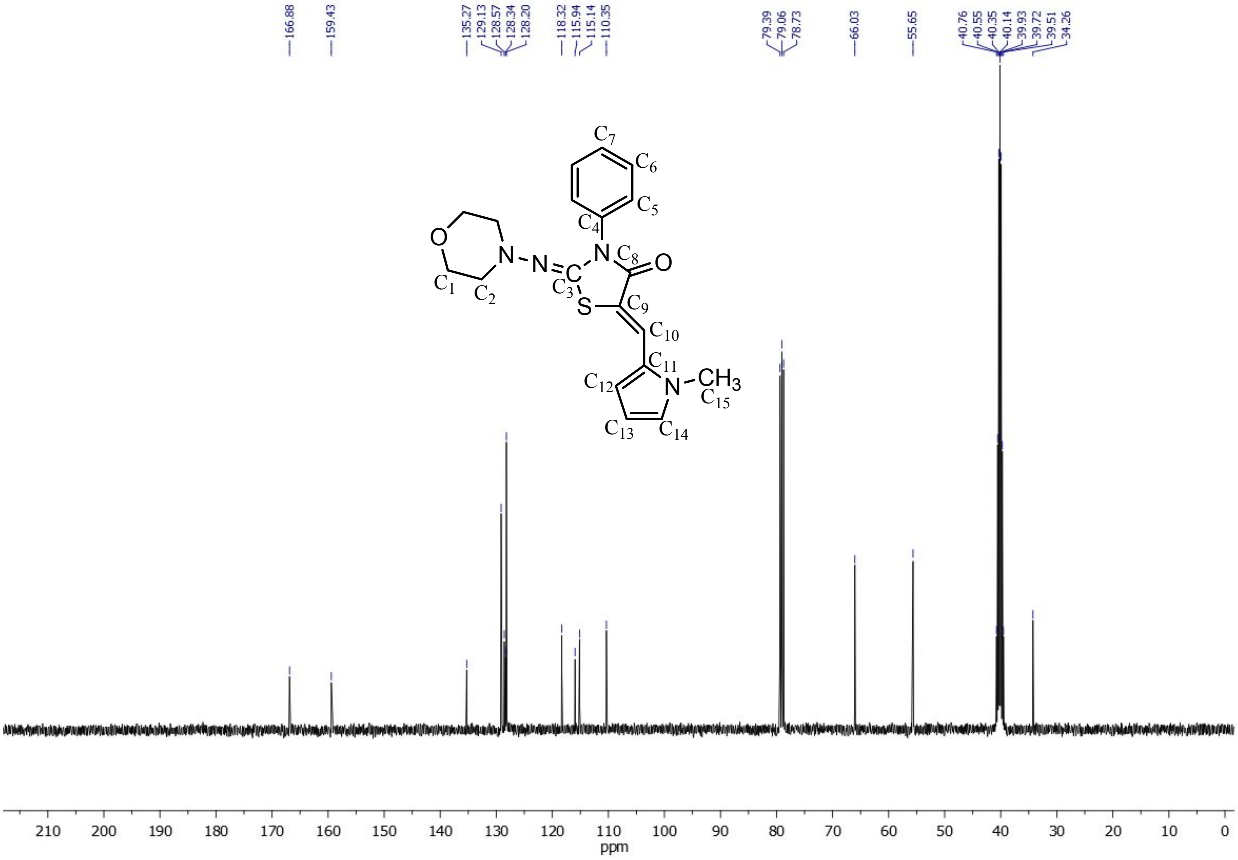

Figure S57. ^{13}C NMR spectrum of compound **19**

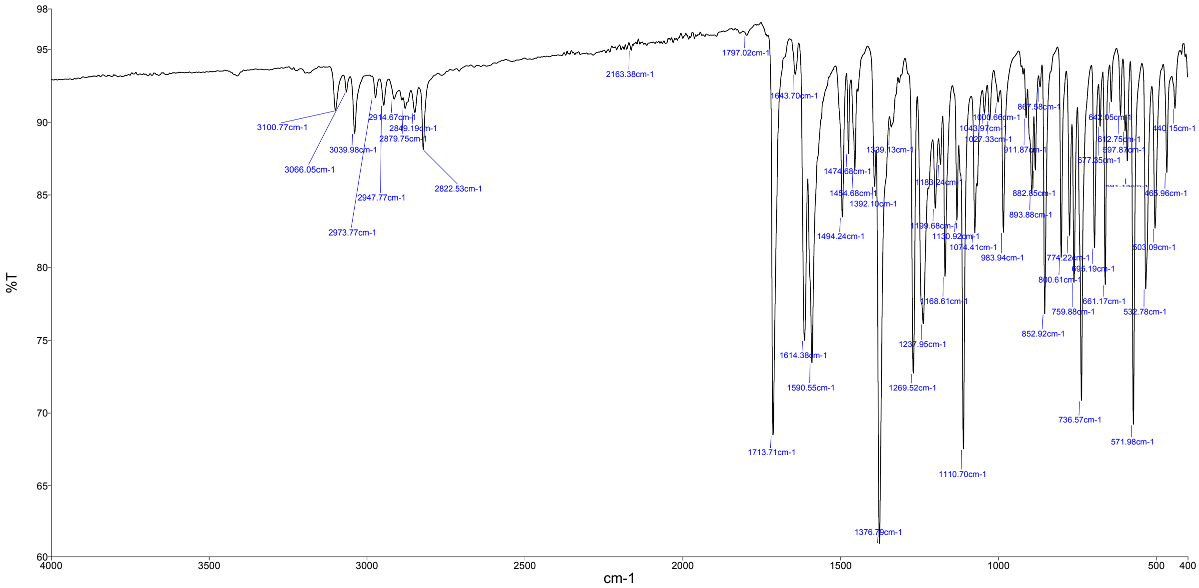

Figure S58. FT-IR spectrum of compound **20**

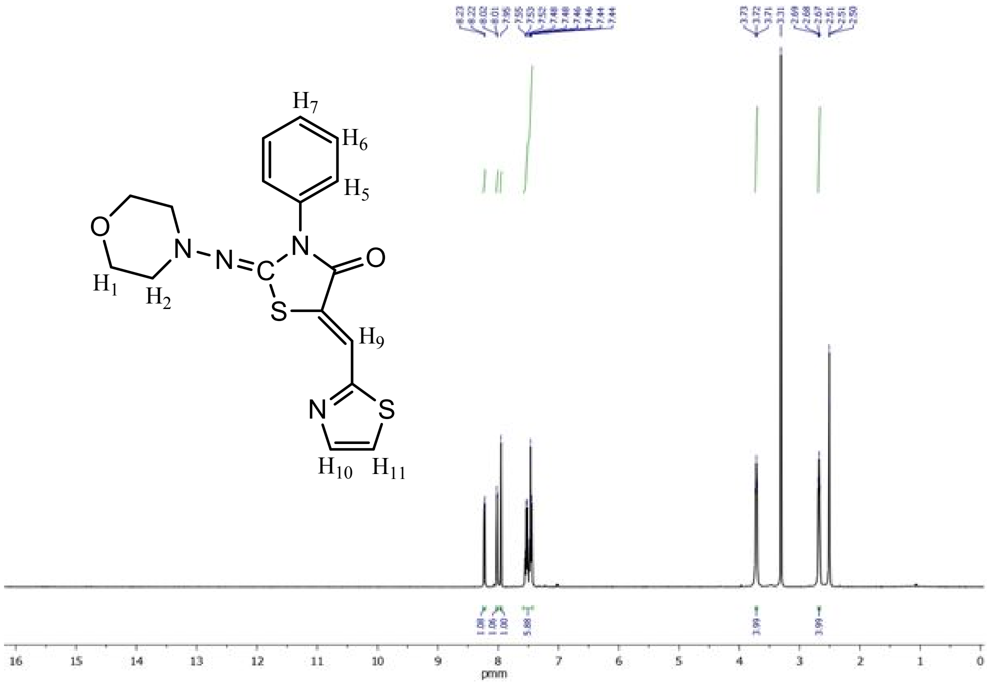

Figure S59. ^{1}H NMR spectrum of compound **20**

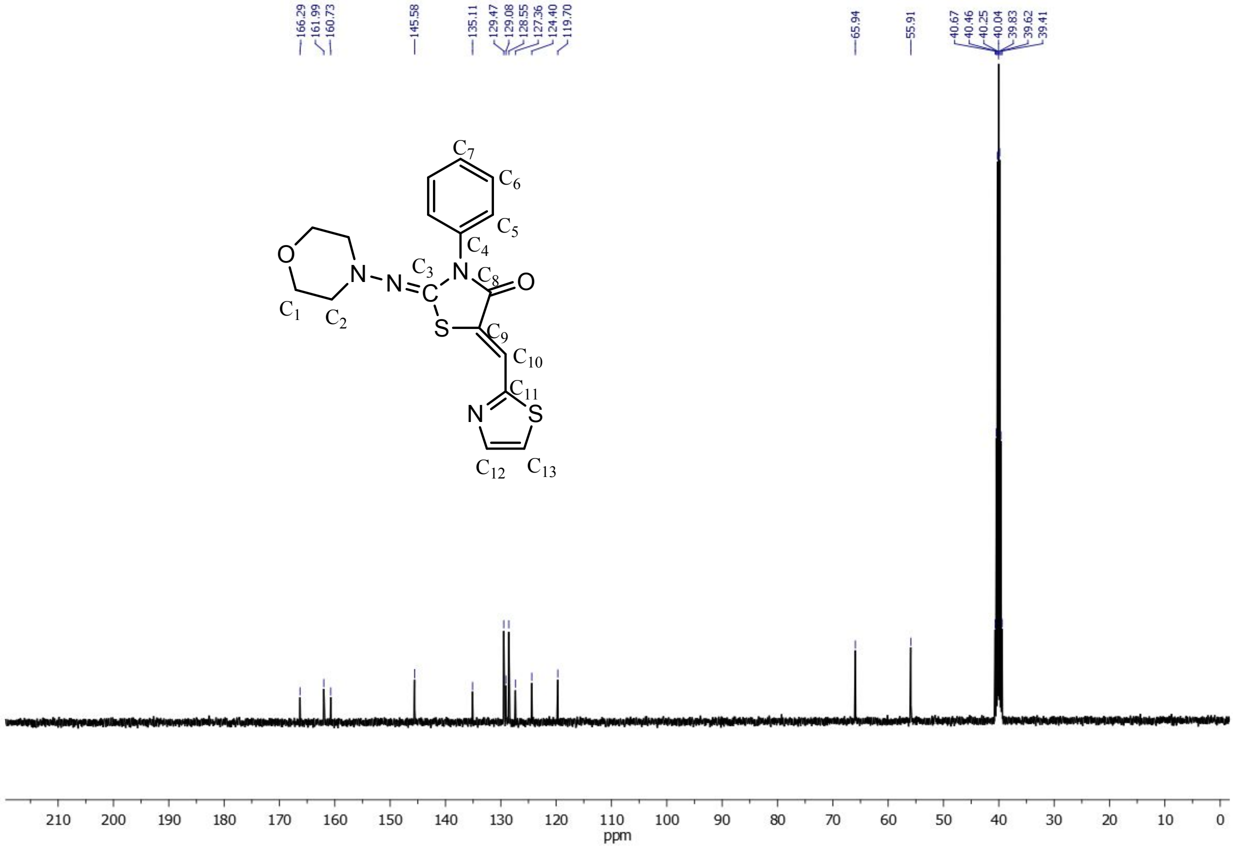

Figure S60. ^{13}C NMR spectrum of compound **20**

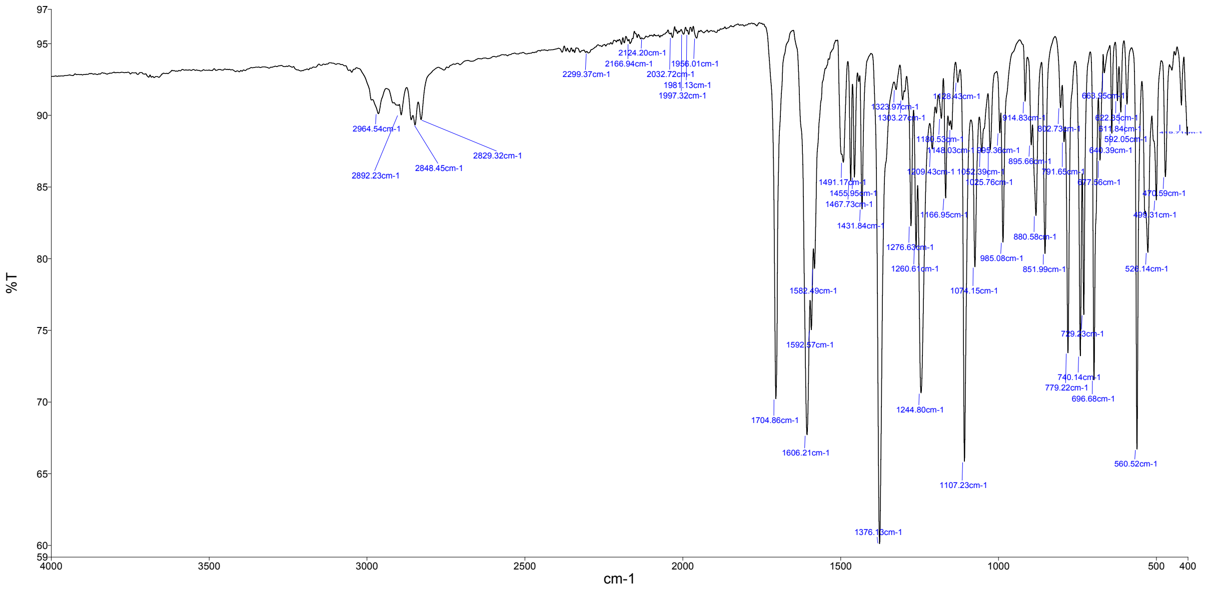

Figure S61. FT-IR spectrum of compound **21**

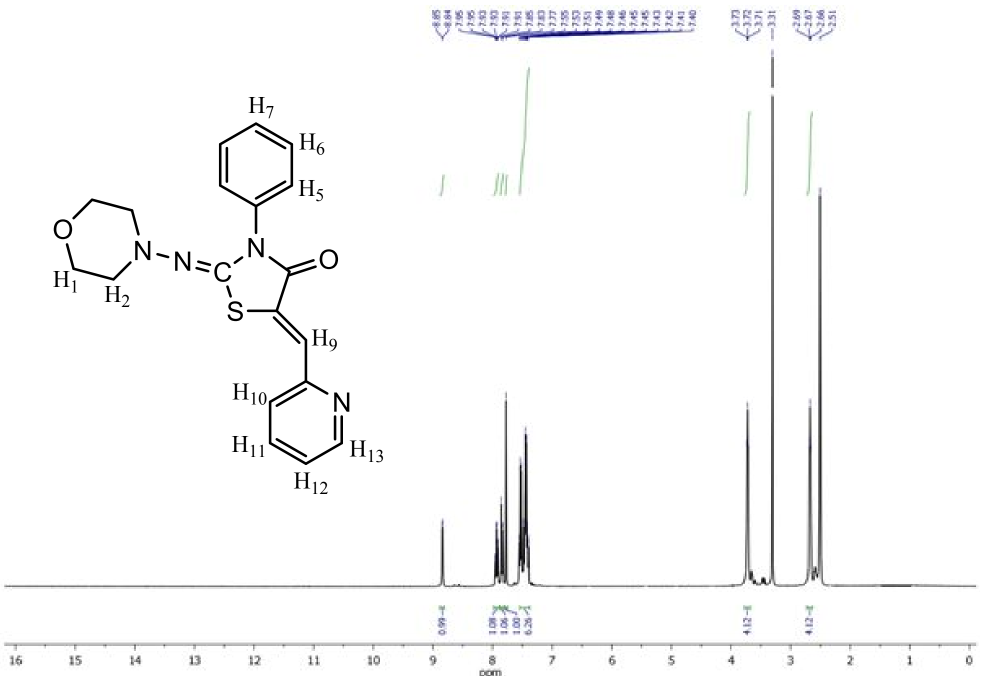

Figure S62. ^{1}H NMR spectrum of compound **21**

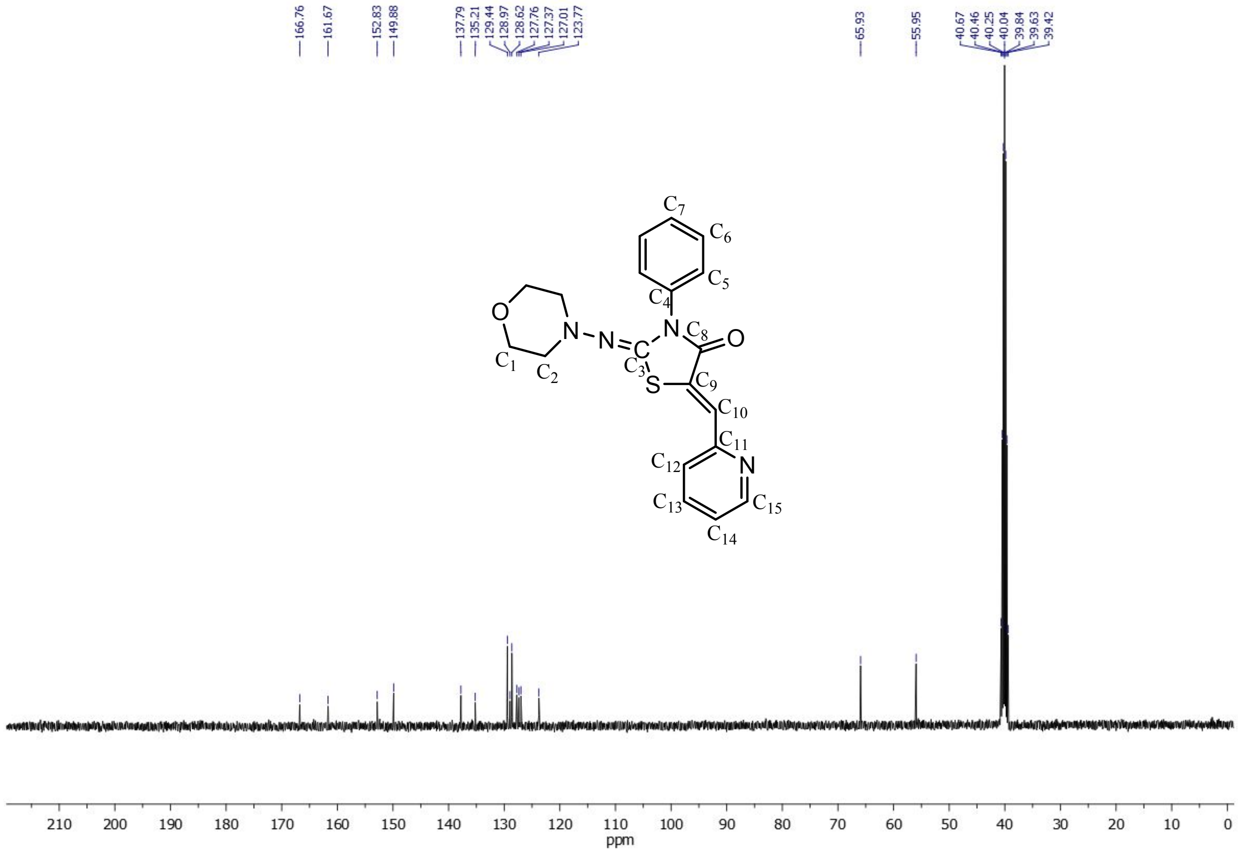

Figure S63. ^{13}C NMR spectrum of compound **21**

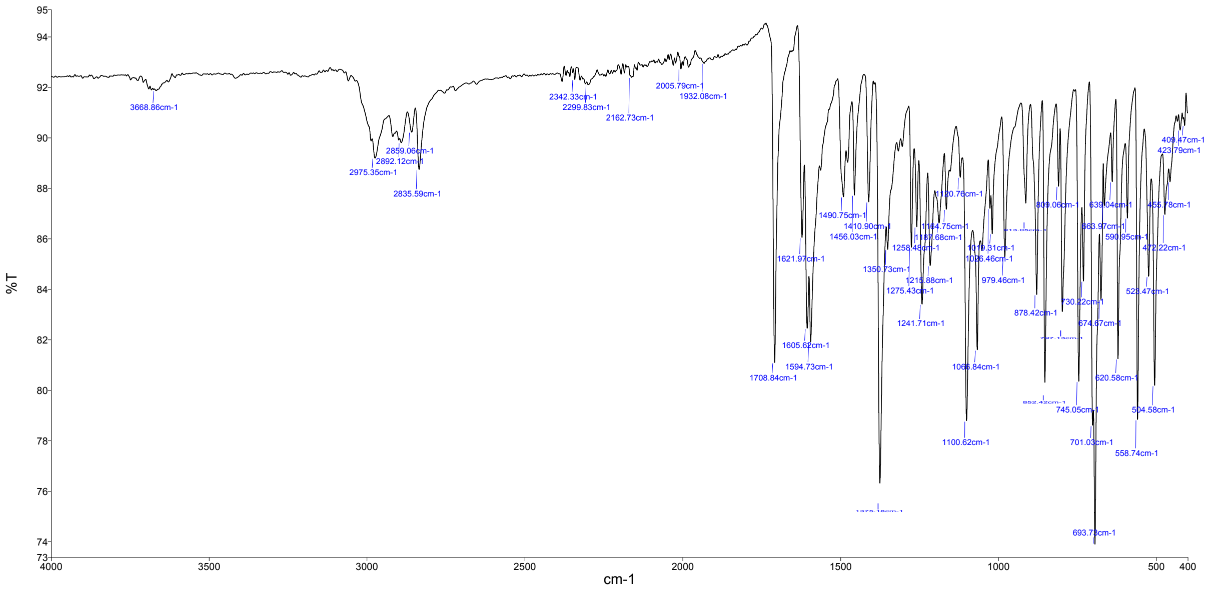

Figure S64. FT-IR spectrum of compound **22**

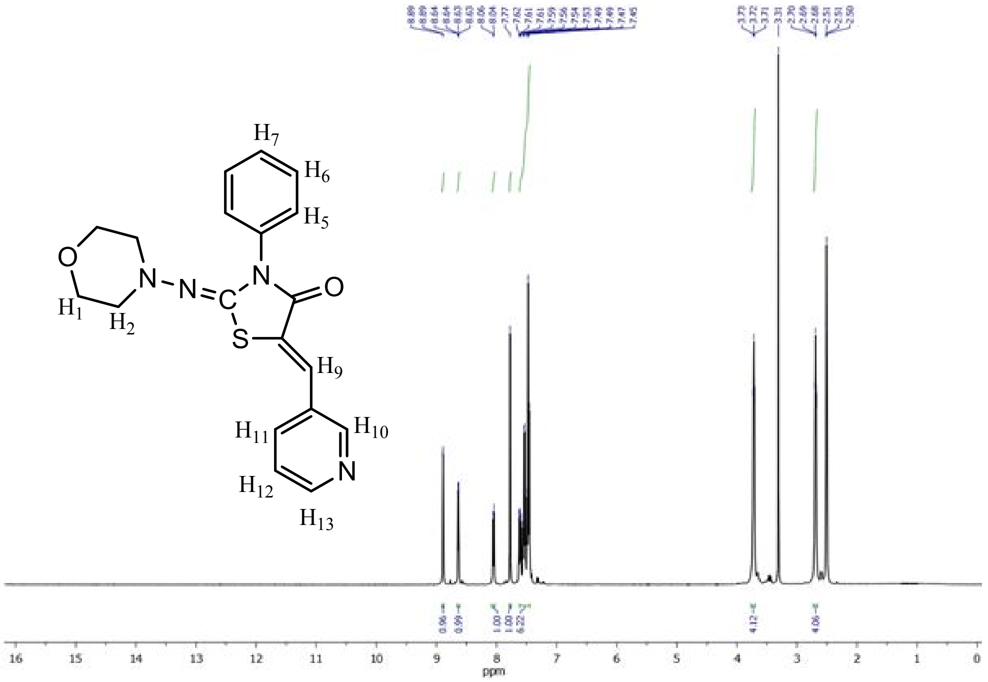

Figure S65. ^{1}H NMR spectrum of compound **22**

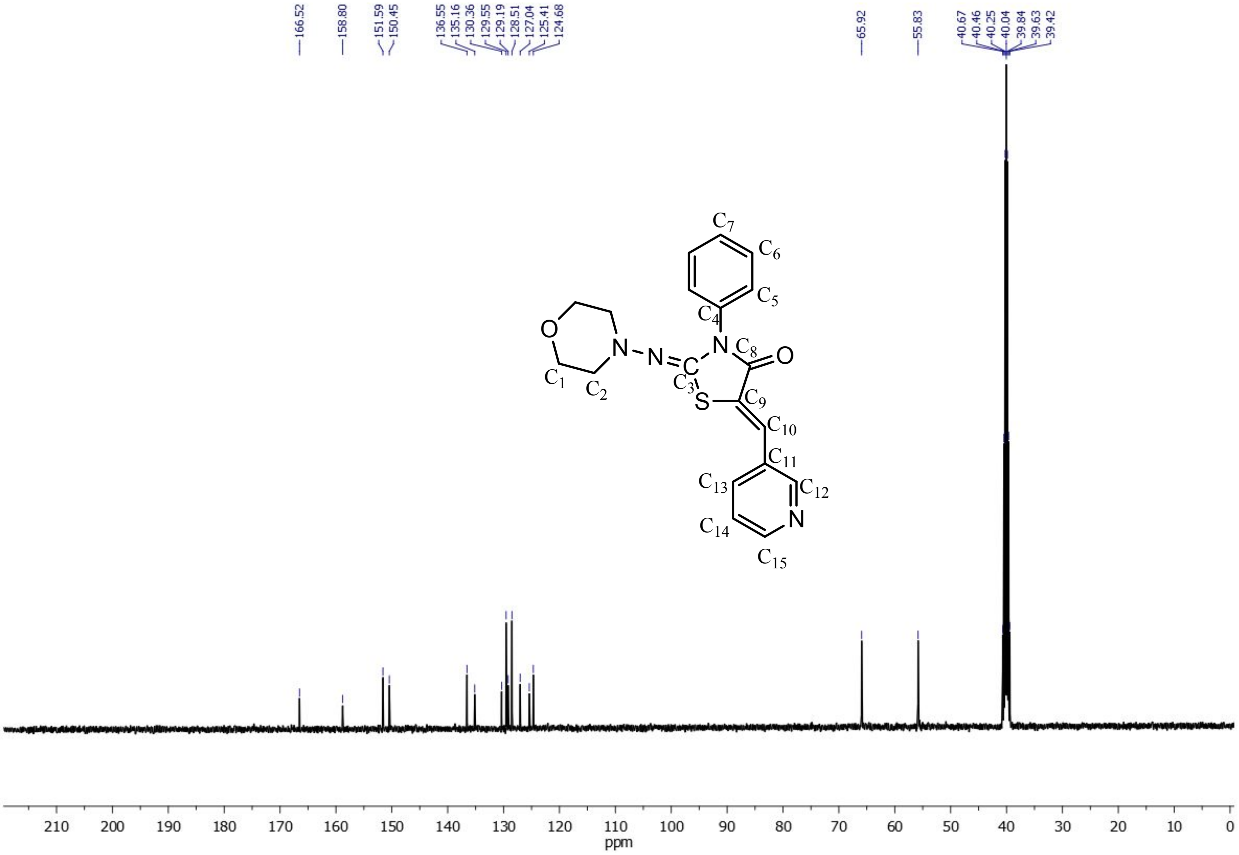

Figure S66. ^{13}C NMR spectrum of compound **22**

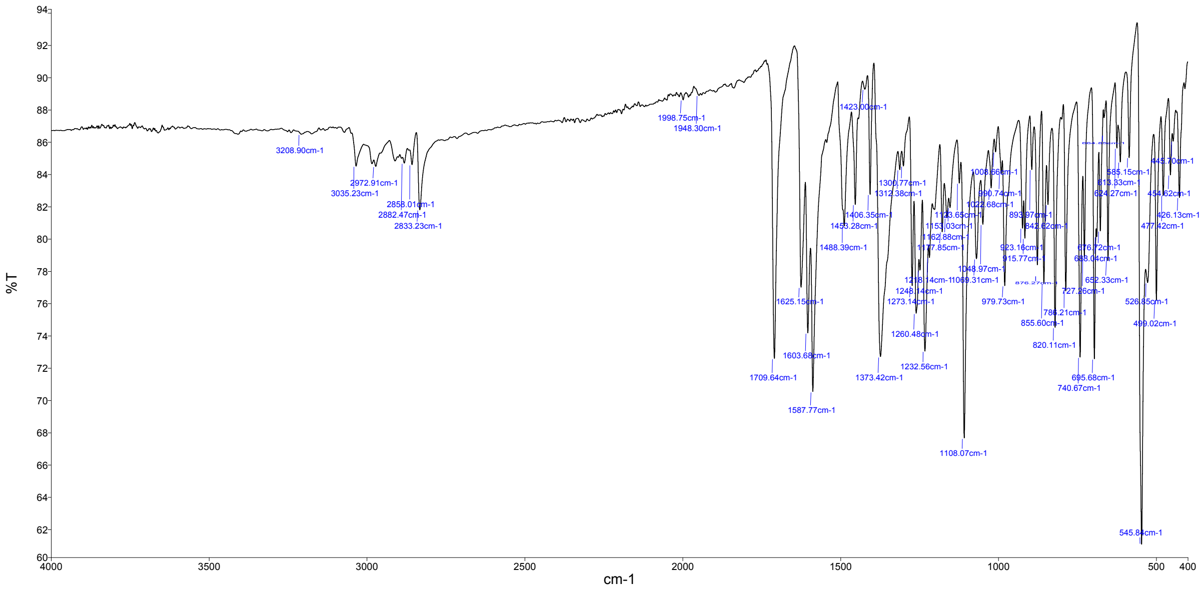

Figure S67. FT-IR spectrum of compound **23**

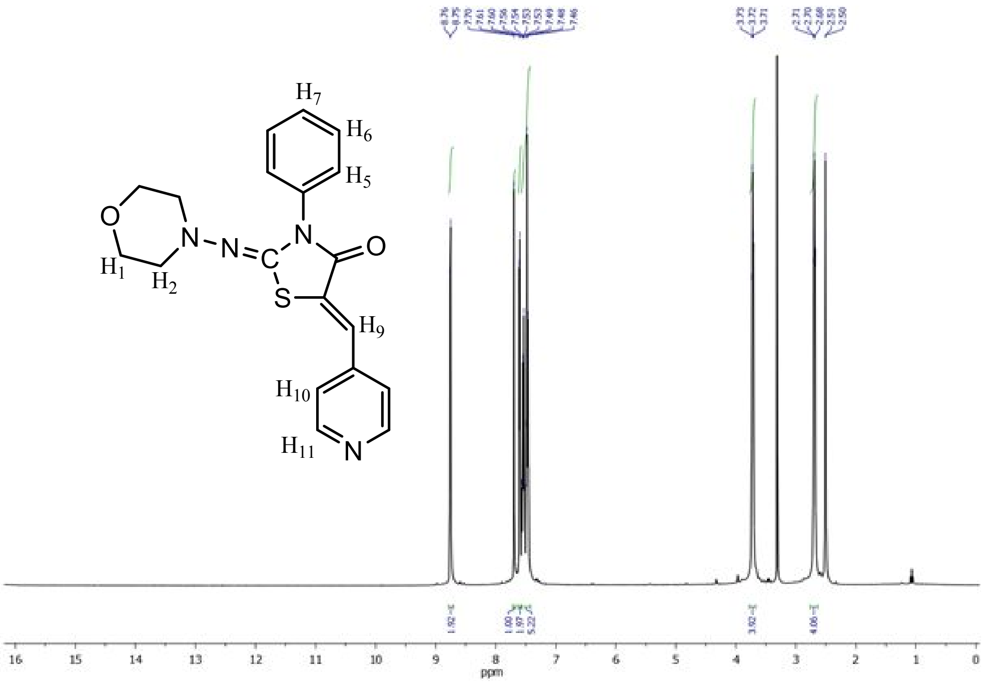

Figure S68. ^{1}H NMR spectrum of compound **23**

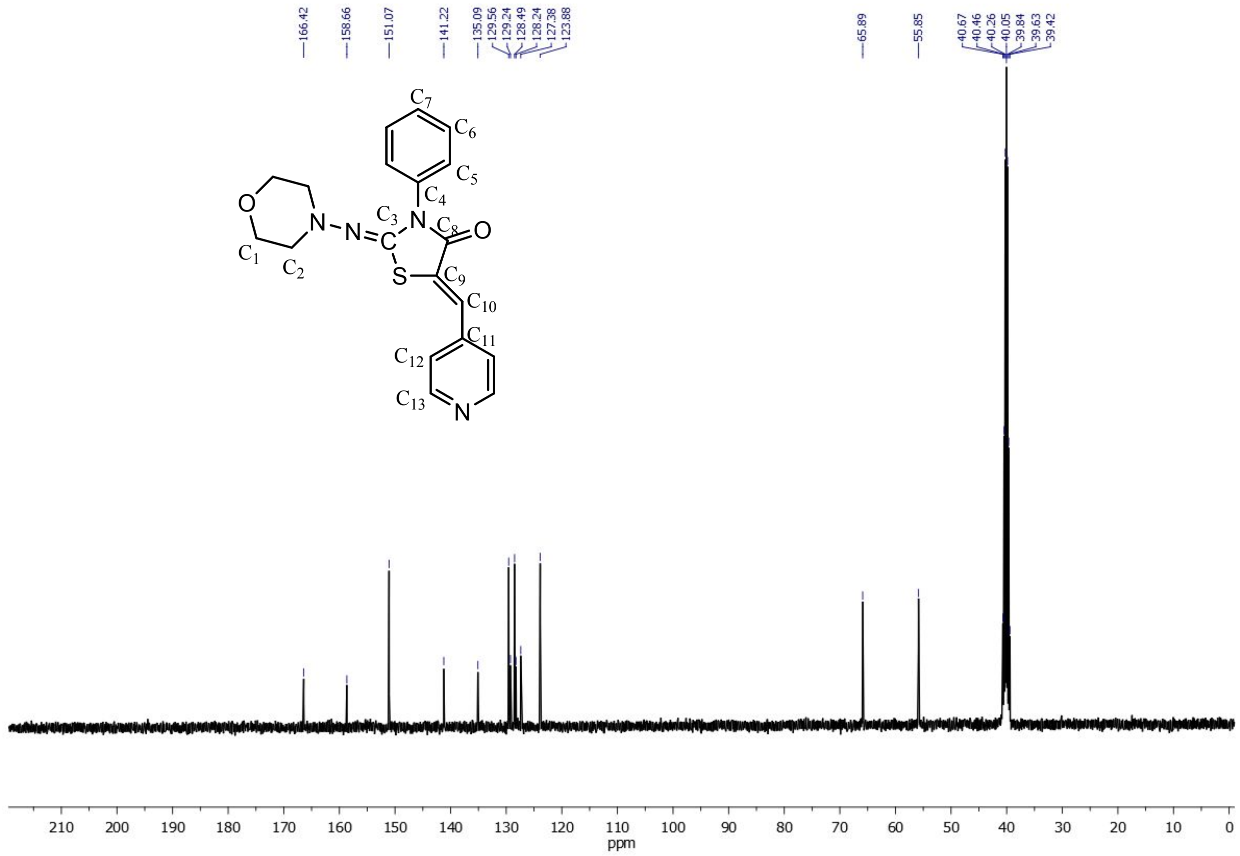

Figure S69. ^{13}C NMR spectrum of compound **23**

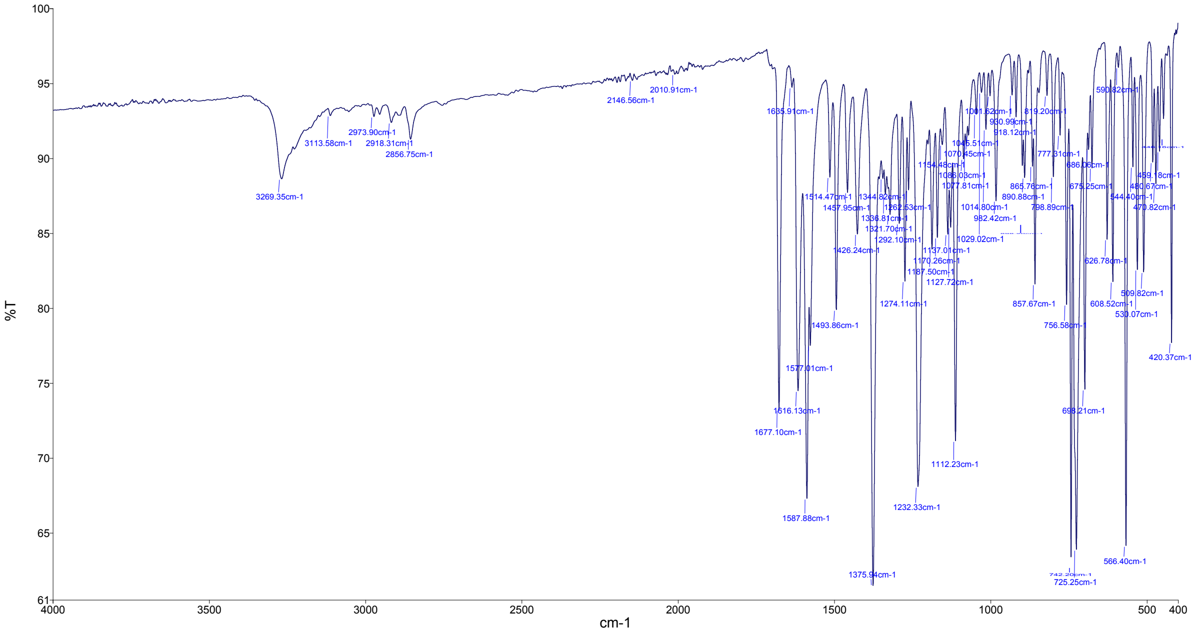

Figure S70. FT-IR spectrum of compound **24**

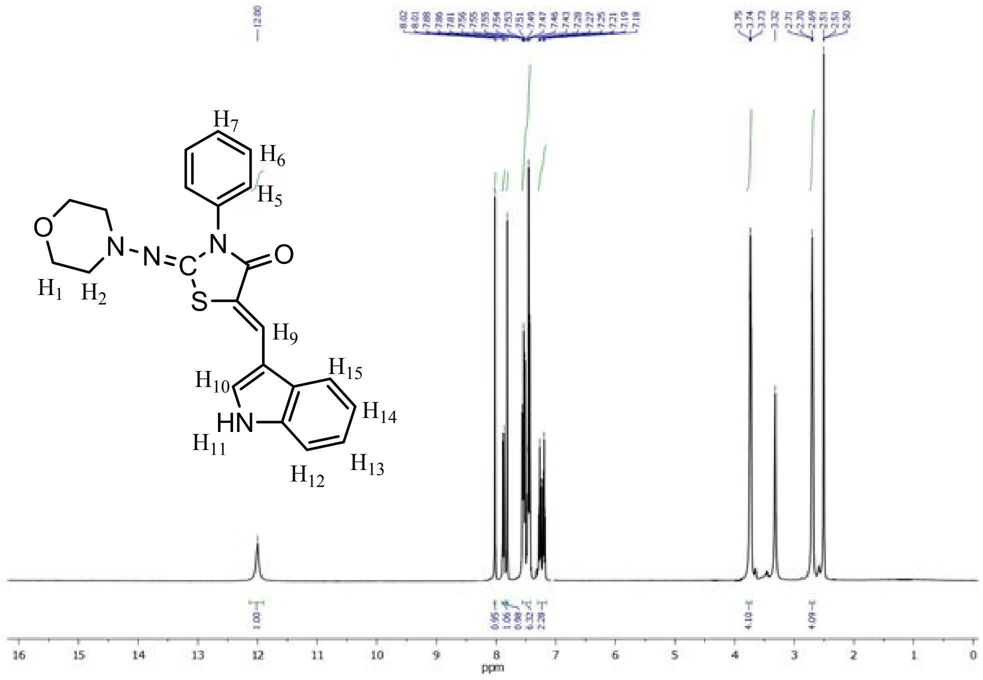

Figure S71. ^{1}H NMR spectrum of compound **24**

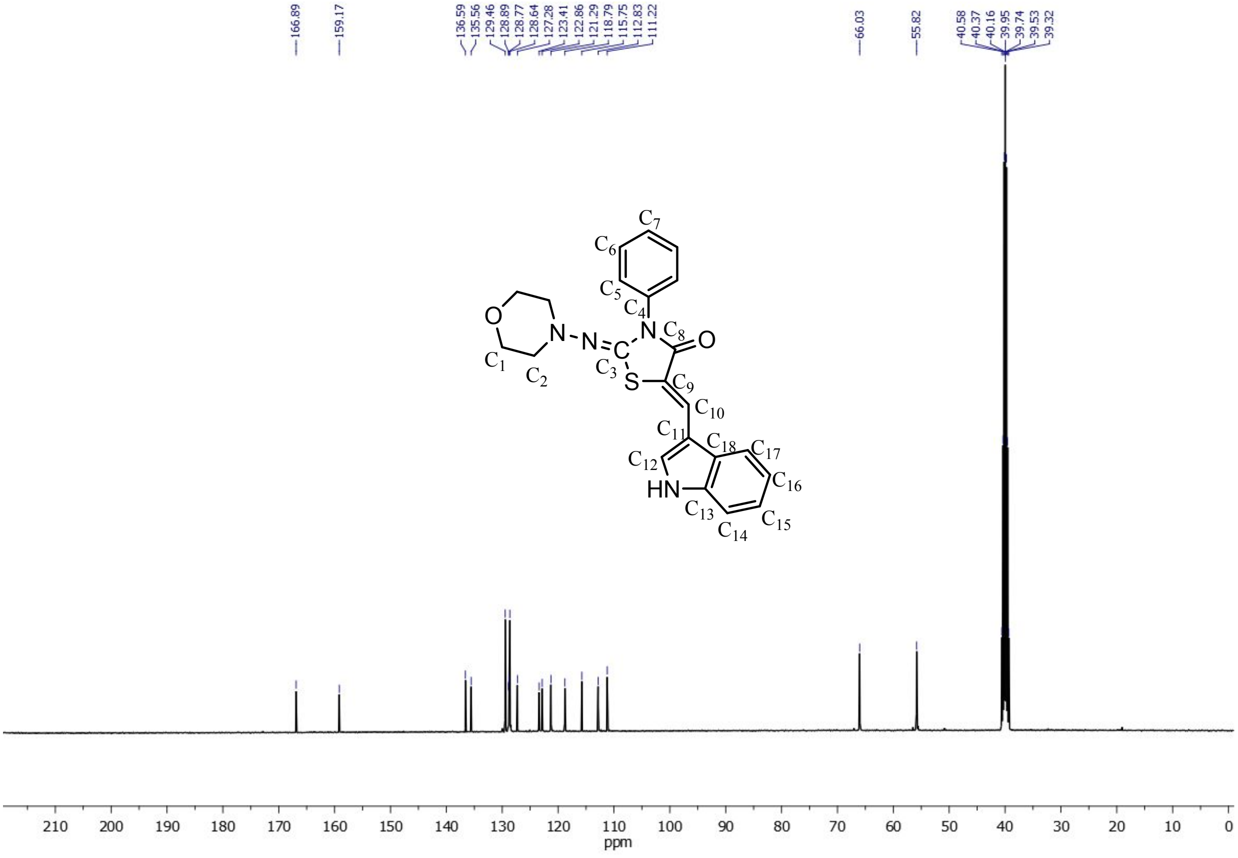

Figure S72. ^{13}C NMR spectrum of compound **24**

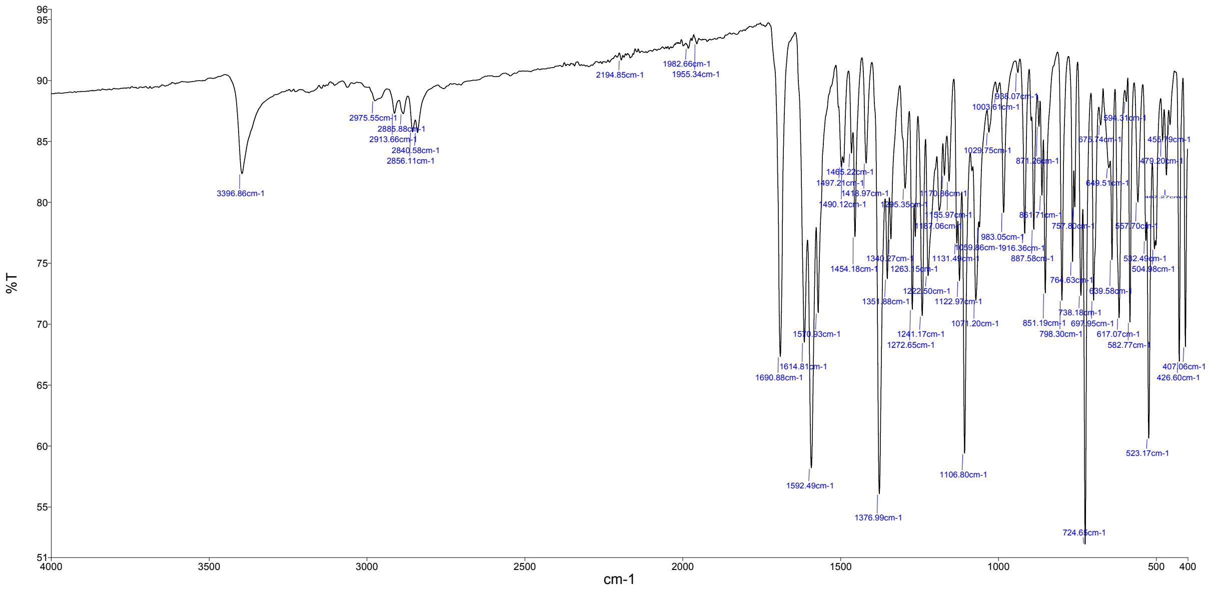

Figure S73. FT-IR spectrum of compound **25**

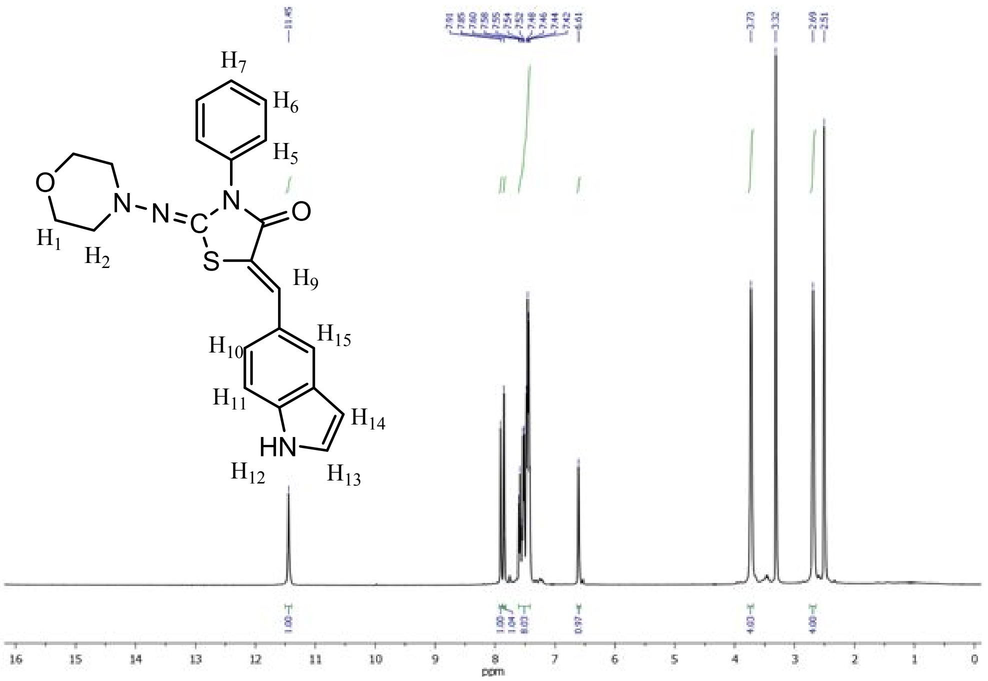

Figure S74. ^{1}H NMR spectrum of compound **25**

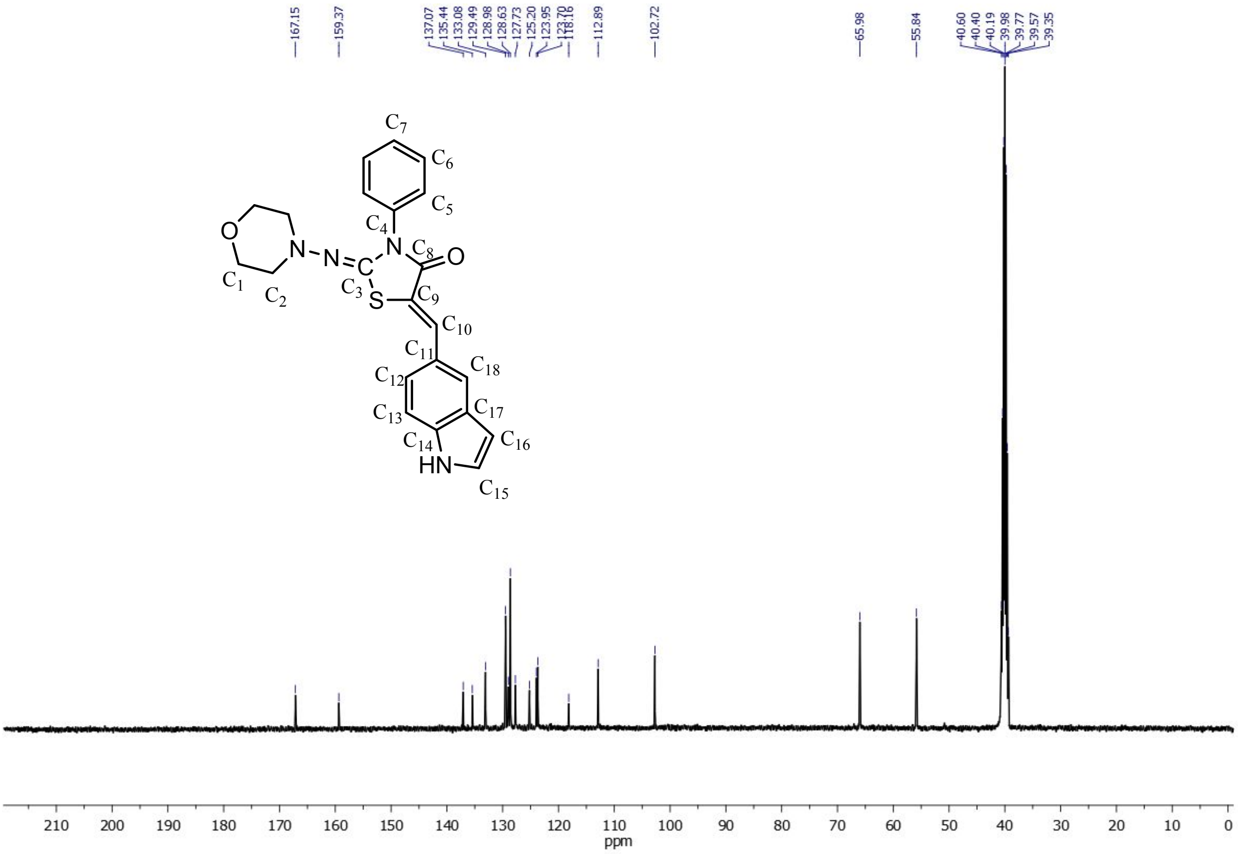

Figure S75. ^{13}C NMR spectrum of compound **25**

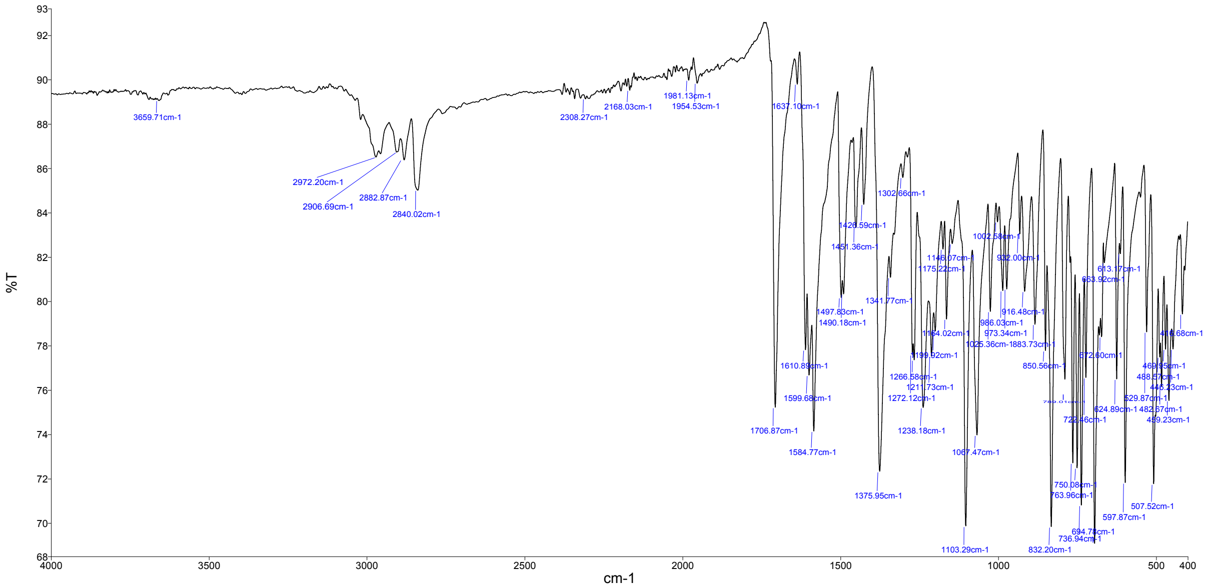

Figure S76. FT-IR spectrum of compound **26**

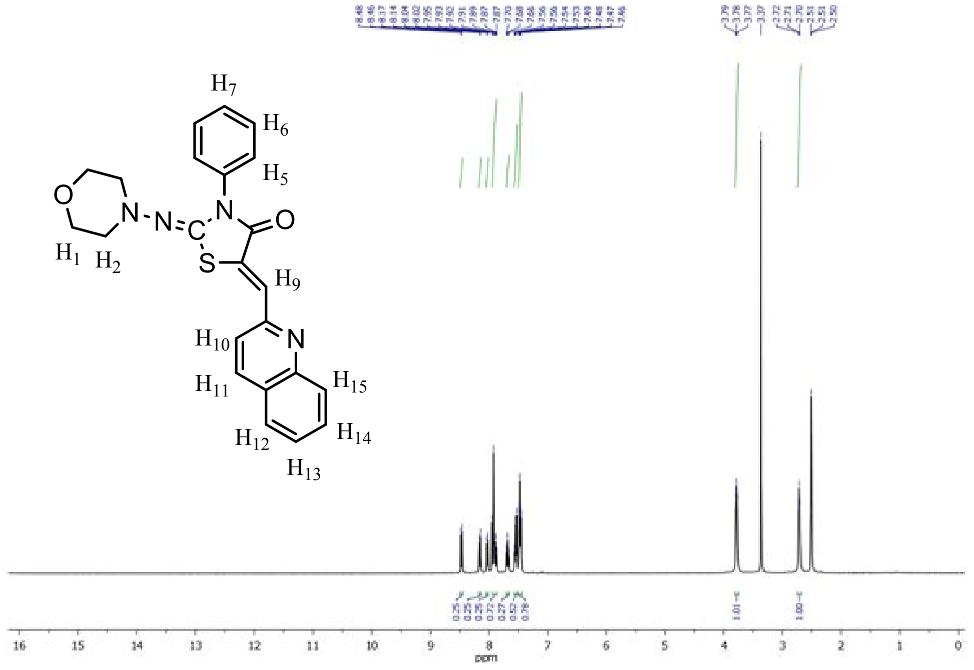

Figure S77. ^{1}H NMR spectrum of compound **26**

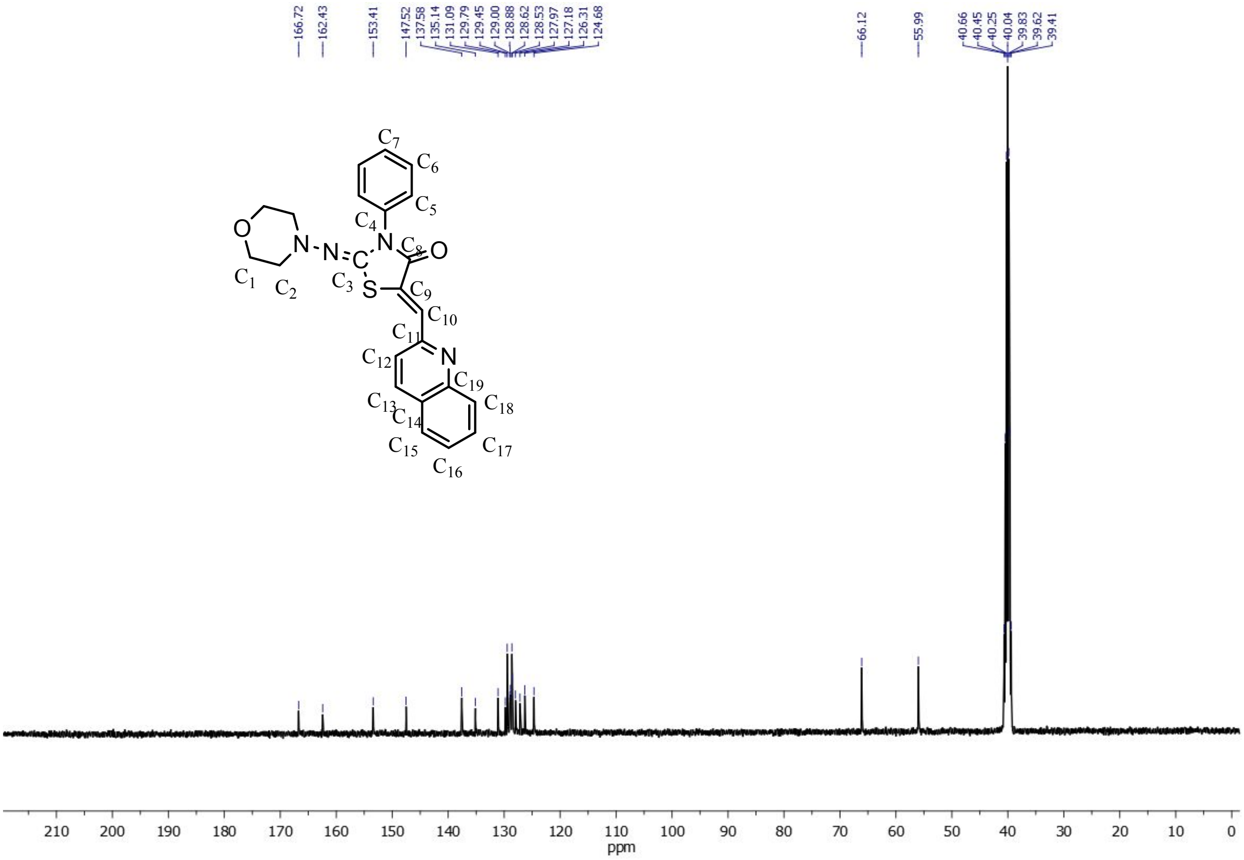

Figure S78. ^{13}C NMR spectrum of compound **26**
